# Supplementary material for: Geospatial patterns, and individual and community levels factors of cesarean section deliveries in Ethiopia: A spatial and multilevel analysis
Source: PLoS One. 2024 Aug 27;19(8):e0306052. doi: 10.1371/journal.pone.0306052 (PMC11349178; doi:10.1371/journal.pone.0306052)
Supplement: Attachment — Submitted filename: PONE-D-23-40362-AF.pdf [file pone.0306052.s001.pdf]

PLOS ONE

Geospatial Patterns, Prevalence, and Individual and Community Levels Factors of Cesarean Section Deliveries in Ethiopia: A Spatial and Multilevel Analysis

--Manuscript Draft--

**Manuscript Number:**	PONE-D-23-40362
**Article Type:**	Research Article
**Full Title:**	Geospatial Patterns, Prevalence, and Individual and Community Levels Factors of Cesarean Section Deliveries in Ethiopia: A Spatial and Multilevel Analysis
**Short Title:**	Geospatial Patterns, and Individual and Community Levels Factors of Cesarean Section Deliveries in Ethiopia:
**Corresponding Author:**	Abel Endawkie, MPH<br>Wollo University<br>Dessie, Amhara ETHIOPIA
**Keywords:**	Cesarean Section, Spatial Pattern, Multilevel, 2019 Mini-EDHS:
**Abstract:**	Abstract<br><br>Background: Cesarean Section (CS) is the most popular ~~surgeries~~ worldwide in obstetric care to save mother's or the child's life. However spatial distribution, prevalence, and multilevel factors of CS in Ethiopia using 2019 Mini EDHS is limited. Therefore, this study aimed to determine geospatial patterns, prevalence, and individual and community levels factors of cesarean section deliveries in Ethiopia<br>Method: The study was analyzed using 2019 mini EDHS with 5527 weighted sample of reproductive age women to determine spatial distribution and multilevel factors of CS in Ethiopia. Significance was declared at p-value <0.05 and AOR with 95% CI was reported.<br>Result: The prevalence of CS Ethiopia was 5.4% (4.6%-6.4%). Age, 15-24 [AOR=1.98, 95% CI=(1.3, 3.1)] and [AOR=3.7, 95% CI=(2.2, 6.1)], female household [AOR=1.9, 95% CI=(1.2, 3.1)], Muslim [AOR=0.4, 95% CI=(0.2, 0.7)], primary [AOR=1.6, 95% CI=(01.07, 2.7)], secondary[AOR=2.4, 95% CI=(1.3, 4.25)], higher education [AOR=2.06, 95% CI (1.03, 4.2)], multiple birth[AOR=8.1, 95% CI=(3.4, 19.74)], Addis Ababa [AOR=3.4 95% CI=(1.1, 11.9)], and Dire Dawa [AOR=7, 95%CI=(1.23, 40.7)] were associated with CS.<br>Conclusion<br>The findings suggest a slight increase in the prevalence of cesarean section (CS) deliveries in Ethiopia, although remaining below the World Health Organization's (WHO) recommended rates. Geospatial analysis highlights significant variations in CS prevalence, with hotspot areas observed in urban regions such as Diredewa, Harerie, and parts of Somali and Oromia. Factors including maternal age, sex of the household head , religion, educational status, and types of birth at the individual level, and region at the community level. Therefore, to align with WHO guidelines, efforts should be directed toward optimizing CS delivery rates and ensuring appropriate distribution across regions in Ethiopia.
**Order of Authors:**	Abel Endawkie, MPH
	Shimels Derso Kebede, MPH
	Natnael Kebede, MPH
	Mengistu Mera Mihiretu, MPH
	Ermias Bekele, MPH
	Kokeb Ayele, MPH
	Lakew Asmare, MPH
	Fekade Demeke Bayou, MPH
	Mastewal Arefaynie, MPH
	Yawkal Tsega, Masters of Health System and Management,

Powered by Editorial Manager® and ProduXion Manager® from Aries Systems Corporation

Opposed Reviewers:	
Additional Information:	
Question	Response
**Financial Disclosure**<br><br>Enter a financial disclosure statement that describes the sources of funding for the work included in this submission. Review the submission guidelines for detailed requirements. View published research articles from *PLOS ONE* for specific examples.<br><br>This statement is required for submission and **will appear in the published article** if the submission is accepted. Please make sure it is accurate.<br><br>**Funded studies**<br>Enter a statement with the following details:<br>• Initials of the authors who received each award<br>• Grant numbers awarded to each author<br>• The full name of each funder<br>• URL of each funder website<br>• Did the sponsors or funders play any role in the study design, data collection and analysis, decision to publish, or preparation of the manuscript?<br><br>Did you receive funding for this work?	The author(s) received no specific funding for this work.
**Competing Interests**<br><br>Use the instructions below to enter a competing interest statement for this submission. On behalf of all authors, disclose any competing interests that could be perceived to bias this work—acknowledging all financial support and any other relevant financial or non-financial competing interests.<br><br>This statement is required for submission and **will appear in the published article** if the submission is accepted. Please make sure it is accurate and that any funding	The authors declare that they have no competing interests.

sources listed in your Funding Information later in the submission form are also declared in your Financial Disclosure statement.<br><br>View published research articles from *PLOS ONE* for specific examples.<br><br>**NO authors have competing interests**<br><br>Enter: *The authors have declared that no competing interests exist.*<br><br>**Authors with competing interests**<br><br>Enter competing interest details beginning with this statement:<br><br>*I have read the journal's policy and the authors of this manuscript have the following competing interests: [insert competing interests here]*<br><br>* typeset	
**Ethics Statement**<br><br>Enter an ethics statement for this submission. This statement is required if the study involved:<br><br>• Human participants<br>• Human specimens or tissue<br>• Vertebrate animals or cephalopods<br>• Vertebrate embryos or tissues<br>• Field research<br><br>Write "N/A" if the submission does not require an ethics statement.<br><br>General guidance is provided below. Consult the submission guidelines for detailed instructions. **Make sure that all information entered here is included in the Methods section of the manuscript.**	N/A

**Format for specific study types**<br><br>**Human Subject Research (involving human participants and/or tissue)**<br>• Give the name of the institutional review board or ethics committee that approved the study<br>• Include the approval number and/or a statement indicating approval of this research<br>• Indicate the form of consent obtained (written/oral) or the reason that consent was not obtained (e.g. the data were analyzed anonymously)<br><br>**Animal Research (involving vertebrate animals, embryos or tissues)**<br>• Provide the name of the Institutional Animal Care and Use Committee (IACUC) or other relevant ethics board that reviewed the study protocol, and indicate whether they approved this research or granted a formal waiver of ethical approval<br>• Include an approval number if one was obtained<br>• If the study involved *non-human primates*, add *additional details* about animal welfare and steps taken to ameliorate suffering<br>• If anesthesia, euthanasia, or any kind of animal sacrifice is part of the study, include briefly which substances and/or methods were applied<br><br>**Field Research**<br><br>Include the following details if this study involves the collection of plant, animal, or other materials from a natural setting:<br>• Field permit number<br>• Name of the institution or relevant body that granted permission	
**Data Availability**<br><br>Authors are required to make all data underlying the findings described fully available, without restriction, and from the time of publication. PLOS allows rare exceptions to address legal and ethical concerns. See the PLOS Data Policy and FAQ for detailed information.	Yes - all data are fully available without restriction

Powered by Editorial Manager® and ProduXion Manager® from Aries Systems Corporation

A Data Availability Statement describing where the data can be found is required at submission. Your answers to this question constitute the Data Availability Statement and **will be published in the article**, if accepted.<br><br>**Important:** Stating 'data available on request from the author' is not sufficient. If your data are only available upon request, select 'No' for the first question and explain your exceptional situation in the text box.<br><br>Do the authors confirm that all data underlying the findings described in their manuscript are fully available without restriction?	
**Describe where the data may be found in full sentences. If you are copying our sample text, replace any instances of XXX with the appropriate details.**<br><br>• If the data are **held or will be held in a public repository**, include URLs, accession numbers or DOIs. If this information will only be available after acceptance, indicate this by ticking the box below. For example: *All XXX files are available from the XXX database (accession number(s) XXX, XXX.*).<br>• If the data are all contained **within the manuscript and/or Supporting Information files**, enter the following: *All relevant data are within the manuscript and its Supporting Information files.*<br>• If neither of these applies but you are able to provide **details of access elsewhere**, with or without limitations, please do so. For example:<br><br>*Data cannot be shared publicly because of [XXX]. Data are available from the XXX Institutional Data Access / Ethics Committee (contact via XXX) for researchers who meet the criteria for access to confidential data.*<br><br>*The data underlying the results presented in the study are available from (include the name of the third party*	Data are available in a public, open access repository. Data for this study were sourced from Demographic and Health surveys (DHS) and available here: https://dhsprogram.com/data/available-datasets.cfm

and contact information or URL).<br>• This text is appropriate if the data are owned by a third party and authors do not have permission to share the data.<br><br>* typeset	
Additional data availability information:	

TO **Plose One**

Date: December 2023

Geospatial Patterns, Prevalence, and Individual and Community Levels Factors of Cesarean Section Deliveries in Ethiopia: A Spatial and Multilevel Analysis

Corresponding author Abel Endawkie

Dear Editor

The new manuscript, entitled as Geospatial Patterns, Prevalence, and Individual and Community Levels Factors of Cesarean Section Deliveries in Ethiopia: A Spatial and Multilevel Analysis, will make a substantial contribution to the science on the geospatial patterns, prevalence, and individual and community levels factors of cesarean section deliveries in Ethiopia: a spatial and multilevel analysis.

We choose, Plose one for this article, because it is a well-indexed, peer-reviewed, open-access journal with a strong contribution to the scientific community. The study bears no resemblance to previously published research. This paper, in its entirety or in parts, has not been published or submitted to elsewhere. It will serve as a resource for researchers interested in dealing with similar issues. As a result, policymakers and program designers will benefit from this research article. There are no conflicts of interest or opposing writers because the manuscript has been reviewed and approved by all authors.

“With Best Regards”

Abel Endawkie **Lecturers and researchers**

Email Adress abelendawkie@gmail.com

Manuscript Click here to access/download;Manuscript;C S Manuscribt.docx

Geospatial Patterns, Prevalence, and Individual and Community Levels Factors of Cesarean Section Deliveries in Ethiopia: A Spatial and Multilevel Analysis

Abel Endawkie[1*] Shimels Derso Kebede[2], Natnael Kebede[3], Mengistu Mera Mihretu[4], Ermias Bekele[2] Kokeb Ayele[3], Lakew Asmare1, Fekade Demeke Bayou[1], Mastewal Arefaynie[5], Yawkal Tsega[4]

Affiliation

[1]Department of Epidemiology and Biostatistics, School of Public Health, College of Medicine and Health Sciences, Wollo University, Dessie, Ethiopia.

2Department of Health Informatics, School of Public Health, College of Medicine and Health Sciences, Wollo University, Dessie, Ethiopia

3Department of Health Promotion, School of Public health College of Medicine Health Sciences, Wollo University, Dessie, Ethiopia 4Department of Medical Nursing.

4Department of Health System and Management, School of Public Health, College of Medicine and Health Sciences, Wollo University, Dessie, Ethiopia

5Department of Reproductive and Family Health, School of Public Health, College of Medicine and Health Sciences, Wollo University, Dessie, Ethiopia

Corresponding author Abel Endawkie

Email Adress **abelendawkie@gmail.com**

Abstract

Background: Cesarean Section (CS) is the most popular surgeries worldwide in obstetric care to save mother's or the child's life. However spatial distribution, prevalence, and multilevel factors of CS in Ethiopia using 2019 Mini EDHS is limited. Therefore, this study aimed to determine geospatial patterns, prevalence, and individual and community levels factors of cesarean section deliveries in Ethiopia

Method: The study was analyzed using 2019 mini EDHS with 5527 weighted sample of reproductive age women to determine spatial distribution and multilevel factors of CS in Ethiopia. Significance was declared at p-value <0.05 and AOR with 95% CI was reported.

Result: The prevalence of CS in Ethiopia was 5.4% (4.6%-6.4%). Age, 15-24 [AOR=1.98, 95% CI=(1.3, 3.1)] and [AOR=3.7, 95% CI=(2.2, 6.1)], female household head [AOR=1.9, 95% CI=(1.2, 3.1)], muslim [AOR=0.4, 95% CI=(0.2, 0.7)], primary [AOR=1.6, 95% CI=(01.07, 2.7)], secondary[AOR=2.4, 95% CI=(1.3, 4.25)], higher education [AOR=2.06, 95% CI (1.03, 4.2)], multiple birth[AOR=8.1, 95% CI=(3.4, 19.74)], Addis Ababa [AOR=3.4 95% CI=(1.1, 11.9)], and Dire Dawa [AOR=7, 95%CI=(1.23, 40.7)] were associated with CS.

Conclusion

The findings suggest a slight increase in the prevalence of cesarean section (CS) deliveries in Ethiopia, although remaining below the World Health Organization's (WHO) recommended rates. Geospatial analysis highlights significant variations in CS prevalence, with hotspot areas observed in urban regions such as Diredewa, Harerie, and parts of Somali and Oromia. Factors including maternal age, sex of the household head, religion, educational status, and types of birth

at the individual level, and region at the community level. Therefore, to align with WHO guidelines, efforts should be directed toward optimizing CS delivery rates and ensuring appropriate distribution across regions in Ethiopia.

Keyword: Cesarean Section, Spatial Pattern, Multilevel, 2019 Mini-EDHS:

Introduction

Cesarean section (CS) is the most usually lifesaving surgical procedure in obstetric care. It is the birth of a fetus through a surgical incision made in the uterine and abdominal walls of a pregnant woman. Preventing maternal and newborn morbidity and mortality rates is the primary purpose for conducting it, either for fetal or maternal reasons(1). Delivery women use CS more frequently than ever before, and this trend is expected to continue in both rich and developing nations over the next ten years(2, 3), even though there is still a significant lack of safe CS in resource-constrained nations. Since 1985, World Health Organization (WHO) states that, the ideal rate for CS should be between 10-15%(4). The average global CS rate, according to recent statistics from 154 nations, is 21.1%; rates range from 8.2% in the least developed countries to 27.2% in the more developed ones Latin America and the Caribbean have the greatest rates (42.8 percent, 23 countries, and 91.2 percent birth coverage), whereas Sub-Saharan Africa has the lowest rates (5.0 percent, 39 nations, and 88.6 percent birth coverage(5). Less than 5% of people have (CS), which indicates an unmet need, according to the WHO recommendation. However, a prevalence of greater than 15% might put the mother and kid at risk(6) This conclusion is further supported by reports from the population-based study, which show that CS rates in the population greater than around 10% are not linked to lower rates of maternal and newborn mortality(7). The number of CS deliveries per 100 births, or the CS rate, has a negative and

statistically significant linear connection with infant death, according to another ecological study (8). Despite all of these facts, the ~~medically non-indicated~~ CS section has seen a large increase in practice in recent years(9). According to data from the WHO survey, women's health and medical expenses may be significantly impacted by the global rise in medically unnecessary CS. These effects may include an increased risk of infection, a longer healing period, significant bleeding, and an increase in iatrogenic injuries, breathing difficulties, and even death(10, 11). The same result was also reached by a multi-country facility-based survey(2). Numerous research demonstrate that a rise in CS has been associated with both medically and non-medically indicated factors, such as the kind of health facility, socio-demographic traits, and maternal health of women(12, 13). Several factors include the mother's age, birth order, area of residence, socioeconomic situation, educational attainment, history of childhood sexual abuse, and obstetric problems, maternal request, and income level (7, 14-17). The prevalence of Cesarean section in Ethiopia is below the WHO's recommended threshold of 10% (6). Furthermore, there has been no notable increase in the country's population-based CS rates in the 2000 and 2016 Ethiopia Demographic and Health Surveys (EDHS), which were found to be very low (0.7 percent and 1.9 percent), respectively(18, 19). This figure is lower than that of several African countries, including Ghana [illegible]% (20) , Nigeria 2.1% (21) , and Mozambique, 4.7 (22) . The relatively low prevalence of CS in the Ethiopian population reflects unmet demands, which may contribute to the country's poor maternal and newborn outcomes(23). Different studies reported different prevalence and associated factors for caesarean section delivery in Ethiopia. Evidence from hospital-based births in Ethiopia indicates that first-parity births, births for which women had more than three Antenatal Care (ANC) visits, urban births, higher education levels, and higher economic statuses are the groups of women most likely to undergo

cesarean delivery ~~by caesarean section~~(3, 5, 14, 15). These studies frequently used small sample sizes and were prone to selection bias(10, 15, 16) and a small sample size and a lack of use of more advanced statistical analysis, like multilevel analysis and spatial analysis were limitations of a few studies that were employed using population-based data. Knowing this evidence spatial distribution, prevalence, and individual and community level factors of caesarean section delivery in Ethiopia using national representative data of 2019 Mini Ethiopian Demographic and Health Survey (MEDHS) is limited, even though it has public health important as evidence of obstetric care .Therefore, this study aimed to determine geospatial patterns, prevalence, and individual and community levels factors of cesarean section deliveries in Ethiopia using spatial and multilevel analysis of 2019 MEDHS national representative data.

Method

Study setting and Period

The study was done in Ethiopia which is located in ~~east Africa which have~~ a diverse population. It is bordered on the west by Sudan, on the east by Somalia and Djibouti, on the north by Eritrea, and the south by Kenya. The country has a total area of 1,112,000 square kilometers Ethiopia is divided into eleven regions and two municipal governments. Tigray, Afar, Amhara, Oromiya, Somali, Benishangul-Gumuz, Southern Nations Nationalities and People (SNNP), Gambela, Sidama, South Western, and Harari are among the regions involved. Addis Ababa and Dire Dawa are administrative cities. Data were accessed from their URL: www.dhsprogram.com by contacting them through personal accounts after justifying the reason for requesting the data(24).

Study design and Source population

The research was conducted using secondary data of cross-sectional study used in 2019MEDHS. This data ~~analyzed~~ from women's fertile and reproductive age (15-49) ~~using surveys conducted on the recent DHS/2019 MEDHS data of Ethiopia using children record.~~

Sample Size and Sampling Methods

A total of 5526.93 ~ 5527 weighted reproductive women were used in this analysis as a sample that was aimed to represent all of the country's regions and administrative cities from 5753 women used in MEDHS 2019. MEDHS 2019 sample was stratified and selected in two stages. In the first stage, stratification was conducted by region, and then each region stratified as urban and rural. The sample size was then allocated using a probability-proportional allocation method. 305 enumeration areas (EAs) were chosen for the 2019MEDHS.

Variable measurement

~~Dependent variables~~ was cesarean section categorized as Yes/No and the individual-level factors including (a[illegible]e, sex of household, religion, wealth index, marital status, educational status, ANC visit, Types of birth, place of delivery,) and community-level factors (region, residence) were considered as independent variable.

Data processing and analysis

Data cleaning was conducted to check for consistency with the MEDHS-2019 descriptive reports. Recoding, variable generation, labeling, and analysis were done by using Stata version 17.0. Descriptive statistics were done to describe the study participants in relation to socio-demographic characteristics which were presented in ta[illegible]s and text. Sample weight was used to

compensate for the unequal probability of selection between the strata that were geographically defined and for non-responses.

Spatial analysis: After spatial autocorrelation was assessed and significant at P-value 0.0001 and Global Mora[illegible]s I =1.0009, we had conducted mapping hotspot analysis using Getis-Ord Gi* hot spot analysis of ArcGIS version 10.7.1 to examine where spatial clusters occur on the landscape by identifying areas with statistically significant clustering of relative high values (hot spots) or relative low values (cold spots). This measure indicates the presence or absence of significant spatial clustering, with the null hypothesis being that there is no difference in characteristic between a unit and its spatial neighbors (25-28).

Mixed effect model: Multilevel analysis was conducted after checking the eligibility of the data to multilevel analysis by using the intra-cluster correction coefficient (ICC). Since ICC is greater than 10% (ICC=44.1%) calculated as ICC = $\frac{\delta 2}{\delta 2+ \pi 2/3}$, where δ2 indicates estimated variance of clusters, multi-level regression was conducted to assess factors related to individual and community-level characteristics of cesarean section. Multilevel analysis was considered appropriate due to the hierarchical nature of the DHS data as well as the estimation of individual and community level effects. The log of the probability of cesarean section was modeled using a two level multilevel model as follows: Log[$\frac{\pi ij}{1-\pi ij}$] $=\beta 0+\beta 1Xij+ B2\ Zij+\mu j+ eij$, Where i and j are individual level and community level(29) ~~unites~~ respectively; X and Z refers to individual and community level variables respectively; πij is the probability of cesarean section for the ith women in the jth community; β's indicates the fixed coefficients. (B0) is the intercept, the effect on the probability of cesarean section in the absence of influencing factors; and μj showed the random effect (the effect of the community on the cesarean section of the jth community) and eij

showed random errors at individual level. By assuming each community had a different intercept (B0) and fixed coefficient (β), the clustered data nature and intra and inter-community variations were taken into account. [illegible]ta organized ~~in to~~, analyses of cesarean section yes/no which has binary response and run mixed effect multi-level logistic regression model by considering two levels (individual and community levels). During analysis first, bi-variable multilevel logistic regression was fitted and variables with p value less than 0.2 at model I and model II were selected to develop the 3rd model (the final model). The analysis was done in four models. The first model was, model-0 (empty model or null model[illegible]he second model was, model-I (analyzing only individual level variable), the 3rd model was, model-II (analyzing only community-level variable), the last model, model-III (analyzing both community level and individual level variables based on the cut of point). Variables with p value less than 0.05 at model-III were significantly associated with cesarean section. The random-effects (variations) were measured by using ICC (model-0), Median Odds Ratio (MOR) in (model-I and II), and Proportional Change in Variance (PCV) was measured to show variation between clusters. ICC shows the variation in cesarean section among women due to community characteristics. MOR is the median value of the odds ratio between the area at highest risk and the area the lowest risk when randomly picking out two areas and it was calculated as MOR=exp.($\sqrt{2 + \delta 2 + 0.6745}$)≈ exp(2.23). In this study, MOR shows the extent to which the individual probability of cesarean section delivery determined by place of residence. PCV measures the total variation attributed by individual-level variables and area-level variables in the final model (model-III). It is calculated as PCV=[δ2 of null model−δ2 of each model)/δ2 of null model]. δ2 of the null model is used as a reference. Multicollinearity was checked among explanatory variables by using standard error at a cut of point±2 and there is no Multicollinearity since, the standard errors were betw[illegible]±2. The

appropriateness of the mixed model (model fitness) is checked using Inter-Class Correlation and model selection was based on Log Likelihood Ratio or AIC/ BIC criteria (**Table 1**).

Table 1 Model fitness and selection

Model	ICC	Variance	PCV	MOR	LLR	AIC	BIC
Model 0	44.1%	2.3	Reference	exp(2.23)	-1202	2408	2421
Model 1		1.67	27.39%	exp(2.08)	-639	1423	1514
Model 2	32.07%	1.7	26.09%	exp(2.09).	-983	1993	2079
Model 3	31.2%	1.63	29.1%	exp(2.07).	-632	1320	1479

Ethical Approval: The Demographic and Health Survey identifies all data before making it public, and the used DHS data sets are openly accessible. There are no variables at the level of human subjects in the WorldPop geographic data. Then an authorization letter was requesting to download EDHS data set was also obtained from CSA after requesting https://dhsprogram.com/. And approvals of dataset and all methods and data of Ethiopian demographic and health survey were performed based on Helsinki principles and Ethiopian research guidelines.

FROM HERE ON, NO MORE COMMENTS WILL BE ADDED. THE ENTIRE PAPER NEEDS TO BE EDITED AND WRITTEN IN STANDARD ENGLISH. PLEASE FIND SOMEBODY WHO CAN DO THAT BEFORE CONTEMPLATING ITS PUBLICATION.

Result

Socio-demographic characters ~~R~~espondents

From a total of 5,753 reproductive age [15–49] women, [illegible] in the analysis. Among this, 51(48%) of women in the age group of 15-24 have ~~deliver~~ via cesarean section, (2.9%) of women who cannot read and write have deliver via cesarean section. Among study participants (11.1%) of women residing in rural areas had deliver via cesarean section (Table 3).

Maternal health service utilization of respondents

In regarding to utilization of maternal health service among study participants ,234 (8.1%) of women who had ANC visit deliver via cesarean section, in regarding to place of delivery 270(10.8) of women who deliver in private hospital deliver via cesarean section(**Table 2**).

Table 2. The maternal health service utilization of respondents who gave birth by cesarean section in Ethiopia Using 2019 MEDHS

Variable	Category	CS delivery			
		No	%	Yes	%
Last delivery by previous CS	Yes	4653	99.6	20	0.4
	No	36	13.3	234	86.7
ANC Visit	No	993	98.9	11	1.1
	Yes	2689	91.99	234	8.01
Types of birth	Single	5109	94.96	271	5.04
	Multiple	118	80.3	29	19.7
Sex of New birth	Male	2692	94.7	150	5.3
	Female	2535	94.4	150	5.6
Place of delivery	Public health facility	2235	89.2	270	10.8
	Private hospital	149	83.2	30	16.8

Spatial distribution of cesarean section delivery

In the 2019 of MEDHS, descriptive spatial analysis showed the areas identified as high clusters(risk for health) of cesarean section delivery include ,Addis Abeba,and diredewa and low cluster (unmet need) were Tigray, Oromia Benshangul gumz, region (Fig1). Spatial autocorrelation showed that there is clustering of CS delivery [MI= 1.009, Zscore =4.99 and

P<0.001] (Fig2). The Hotspot analysis showed that hotspot[high prevalence in Diredewa Harerie, Somali and Oromia region] and cold spot [low prevalence of mixed milk feeding in Tigray, Amahara, Afar, and Benshangul gumz, region](Fig3).

Prevalence of Cesarean Section Delivery in Ethiopia

The prevalence of cesarean section delivery was 5.4% (95% CI (4.6%-6.4%)) in Ethiopia analyzed using 2019 EMDHS

Individual and Community Level Factors Associated with Cesarean Section Delivery

In the final model (model-III) maternal age, sex of the household, religion, educational status, types of birth from individual level variable and, region from community-level variable had a statistical association with cesarean section delivery. The probability of having cesarean section delivery was 1.98 and3.7 times more among participants who are in the age group 25-34 and 35-49 as compared to women in the age group15-24 [AOR=1.98, 95% CI=(1.3, 3.1)] and [AOR=3.7, 95% CI=(2.2, 6.1)] respectively. The odds of cesarean section delivery was 1.9 times more among participants who are female household as compared to male household [AOR=1.9, 95% CI=(1.2, 3.1)]. The odds of cesarean section delivery was 60% less likely among participants who are Muslim religion followers as compared to Christian religion followers [AOR=0.4, 95% CI=(0.2, 0.7)]. Women who were attending primary education, secondary education and higher education were 1.6, 2.4 and 2.06 more likely to have cesarean section delivery when compared with not attend school [AOR=1.6, 95% CI=(01.07, 2.7)], [AOR=2.4, 95% CI=(1.3, 4.25)], [AOR=2.06, 95% CI (1.03, 4.2)] respectively. The odds of cesarean section delivery was 8.1 times more for multiple birth compared with never single birth [AOR=8.1, 95% CI=(3.4, 19.74)]. Women who were living in Addis Ababa and Dire Dawa were

3.6 and7 more likely to have cesarean section delivery when compare to women who live in Tigray region [AOR=3.4 95% CI=(1.1, 11.9)], and [AOR=7, 95%CI=(1.23, 40.7)] respectively(Table 4).

Random effects (measures of variation)

Cesarean section delivery varies significantly across each cluster. ICC indicated 44.1% of the variation in cesarean section delivery among women was attributed to community-level factors. PCV (proportional change of variability) in the final model shows 29.1% of the variation in cesarean section delivery across communities was explained.

Discussion

This study used the 2019 MEDHS data to analyze the spatial distribution, prevalence of CS and determine the factors that associated with CS delivery in Ethiopia. The finding of this study indicates that CS prevalence was 5.44% in Ethiopia. The finding is almost similar study conducted in Mozambique(4.7%)(22), and Uganda(5.22%)(30). However, this prevalence is less than the research conducted in Nepal (9%) (31), Egypt (40.1%)(32), Bangladesh (63%) (33), and China (37%) (34). This is slightly increased from the previous EDHS study result in Ethiopia(18, 19). This indicates that CS availability has slightly improved, however it is still below the WHO recommendation which indicates unmet need as per the WHO statement (4, 6). The unmeet rate of CS and geographical disparities in region of Ethiopia in the current study could be attributed to a number of factors, including inadequate and/or sparse availability of emergence obstetrics services across the nation, inadequate equipment and medication in obstetric emergency centers, a shortage of skilled birth attendants and services, long travel distances, and unsuitable terrain that could provide a substantial geographic barrier to emergency

obstetric treatment(35). Related to factors, maternal age, sex of the household, religion, educational status, types of birth from individual level variable and, region from community-level variable had a statistical association with cesarean section delivery. The probability of having cesarean section delivery was high among participants who are in the age group 25-34 and 35-49 as compared to women in ~~the age group~~. This finding is supported ~~the by~~ study in Vietnam (36), Egypt(32), and Mozambique(22). The reason might be since the risk of delivery problems as well as the chance of premature birth and infant death increased as women got older, excessive bleeding during labor, prolonged labor lasting more than 20 hours, and dysfunctional labor that does not progress to the next stage. Furthermore, diabetes and hypertension during pregnancy were more common in older pregnant women; this may lead to increased delivery by CS (37). The odds of women who live with female household head had more likely delivered by cesarean section than women who live with female household head. ~~Theirs~~ is no evidence support this finding (14-16, 34, 38). Muslim religion follower women were less likely delivered by cesarean section than Orthodox religion follower women. This finding is supported by study conducted in Nigeria(21) . Women who were living in Addis Ababa and Dire Dawa were; more likely to have cesarean section delivery as compared to women who live in Tigray region(39). The odds of cesarean section delivery among women who had completed primary, secondary, or higher education had more likely than women who had not attend education. This finding is supported by study conducted at global, regional and national representative of 150 countries using secondary data from 1990–2014, Ethiopia, Vietnam, Tanzania (2, 23, 34, 36, 40, 41), The possible reason a higher degree of education enhances the possibility of CS delivery might be women who had received education aware of the costs and benefits of using maternity services and also have more confidence and self-reliance for any decision provided by a health care

provider, which in turn, may raise the chance that could get CS service, besides, evidence from health literature demonstrates the fact that health literacy is linked to educational achievement could lead to believe that highly educated women are more likely to seek health services such as CS delivery than their no educated counterparts (42). In this finding, multiple pregnancies were significant factors for CS delivery. Few studies support this finding, a study in Egypt (32) [9], a study in Nigeria(21). The possible reason for this could be multiple pregnancies have been linked to obstetric complications like preterm labor, premature rupture of membranes, malposition, and male presentation of the fetus. It also increased risks of complications to the mother like gestational diabetes, gestational hypertension, preeclampsia, and intrahepatic cholestasis, all of which may increase the likelihood of giving birth via CS. The strength of research was using nationally representative datasets, which gives sufficient statistical power and the study estimates completed after the data had been weighted to allow for probability sampling and nonresponse to make it representative at national and regional levels. The limitation of this study ~~was some factors that affecting~~ CS delivery, like birth weight, maternal weight and height to generate BMI were incomplete in the 2019 mini EDHS data.

Conclusion

The prevalence of CS is slightly increased in Ethiopia, and it is below WHO recommendation. The spatial distribution of cesarean section delivery in Ethiopia reveals notable variations across different regions. Hotspot areas, particularly in urban regions like Diredewa, Harerie, and parts of Somali and Oromia, indicate specific areas with higher prevalence. Maternal age, sex of the household head, religion, educational status, types of birth from individual level factor and, region from community-level factor had a statistical association with cesarean section delivery.

Therefore the implementation of cesarean section delivery should be continuing to ideal rate of CS delivery as per WHO recommendation.

Acknowledgments: The authors are sincerely grateful to the Demographic Health Survey (DHS) program for providing us to use Ethiopian mini DHS dataset through their archives (archive@dhsprogram.com).

Author Contributions: AE contributed soft wear and data analysis. All author contributed in conceptualization, methodology writing, writing the original draft, AE: do formal analysis using software, AE, MA,YT, NK: do writing – review & editing:

List of Abbreviation: AIC: Akakian information Criteria, AOR: Adjusted odds ratio, ANC: Antenatal care, BIC: Bayesian information Criteria, COR :Crud odds ratio, EA: Enumeration Area , ICC: Intra-Class Correlation, MEDHS: Mini Demographic and Health Survey, MOR: Median Odds Ratio, PCV: Proportional Changes of Variance, SNNP: South Nation

Reference

1. Jauniaux E, Grobman W. Textbook of caesarean section: Oxford University Press; 2016.
2. Boerma T, Ronsmans C, Melesse DY, Barros AJ, Barros FC, Juan L, et al. Global epidemiology of use of and disparities in caesarean sections. The Lancet. 2018;392(10155):1341-8.
3. Batieha A, Al-Daradkah S, Khader Y, Basha A, Sabet F, Athamneh T, et al. Cesarean section: incidence, causes, associated factors and outcomes: a national prospective study from Jordan. Gynecol Obstet Case Rep. 2017;3(3):55.
4. WHO. WHO statement on caesarean section rates. Sexual and Reproductive Health and Research. 2015.

5. Betrán AP, Ye J, Moller A-B, Zhang J, Gülmezoglu AM, Torloni MR. The increasing trend in caesarean section rates: global, regional and national estimates: 1990-2014. PloS one. 2016;11(2):e0148343.

6. Betrán AP, Torloni MR, Zhang J-J, Gülmezoglu A, Aleem H, Althabe F, et al. WHO statement on caesarean section rates. Bjog. 2016;123(5):667.

7. Ye J, Zhang J, Mikolajczyk R, Torloni MR, Gülmezoglu A, Betran A. Association between rates of caesarean section and maternal and neonatal mortality in the 21st century: a worldwide population- based ecological study with longitudinal data. BJOG: An International Journal of Obstetrics & Gynaecology. 2016;123(5):745-53.

8. Gjonej R, Poloska A, Keta M, Delija Z, Zyberaj F, Bezhani V, et al. The reasons of rising trend of cesarean section rate year after year. A retrospective study. International Journal of Nursing and midwifery. 2015;7(1):9-15.

9. Harrison MS, Goldenberg RL. Cesarean section in sub-Saharan Africa. Maternal health, neonatology and perinatology. 2016;2(1):1-10.

10. Gebremedhin S. Trend and socio-demographic differentials of Caesarean section rate in Addis Ababa, Ethiopia: analysis based on Ethiopia demographic and health surveys data. Reproductive health. 2014;11(1):1-6.

11. Yisma E, Smithers LG, Lynch JW, Mol BW. Cesarean section in Ethiopia: prevalence and sociodemographic characteristics. The Journal of Maternal-Fetal & Neonatal Medicine. 2019;32(7):1130-5.

12. Ayalew M, Mengistie B, Dheressa M, Demis A. Magnitude of cesarean section delivery and its associated factors among mothers who gave birth at public hospitals in Northern Ethiopia: Institution-based cross-sectional study. Journal of multidisciplinary healthcare. 2020:1563-71.

13. Gutema H, Shimye A. cesarean section and associated factors at mizan aman general hospital, southwest Ethiopia. J Gynecol Obstet. 2014;2(3):37-41.

14. Sakae TM, Freitas PF, d'Orsi E. Factors associated with cesarean section rates in a university hospital. Revista de saude publica. 2009;43:472-80.

15. Taye MG, Nega F, Belay MH, Kibret S, Fentie Y, Addis WD, et al. Prevalence and factors associated with caesarean section in a comprehensive specialized hospital of Ethiopia: a cross-sectional study; 2020. Annals of Medicine and Surgery. 2021;67:102520.

16. Tsega F, Mengistie B, Dessie Y, Mengesha M. Prevalence of cesarean section in urban health facilities and associated factors in Eastern Ethiopia: hospital based cross sectional study. J Preg Child Health. 2015;2(3):169-73.

17. Hernández-Vásquez A, Chacón-Torrico H, Bendezu-Quispe G. Differences in the prevalence of cesarean section and associated factors in private and public healthcare systems in Peru. Sexual & Reproductive Healthcare. 2020;26:100570.

18. E E. demographic and health survey 2000: key indicators report. . The DHS Program ICF:. 2000;206.

19. E E. demographic and health survey 2016: key indicators report. The DHS Program ICF. 2016;363: 364.

20. Rockville M, USA: GSS, GHS, and ICF International. Ghana demographic and health survey 2014. Ghana Statistical Service GHS, ICF International (2015). 2014.

21. Adewuyi EO, Auta A, Khanal V, Tapshak SJ, Zhao Y. Cesarean delivery in Nigeria: prevalence and associated factors—a population-based cross-sectional study. BMJ open. 2019;9(6):e027273.

22. Long Q, Kempas T, Madede T, Klemetti R, Hemminki E. Caesarean section rates in Mozambique. BMC pregnancy and childbirth. 2015;15(1):1-9.

23. Tsegaye H, Desalegne B, Wassihun B, Bante A, Debalkie M, Yeheyis T. Prevalence and associated factors of caesarean section in Addis Ababa hospitals, Ethiopia. Pan African Medical Journal. 2019;34(1).

24. tw b. 1. bank tw (Jul 15, 2021) The world bank Ethiopia. 2021.

25. Kulldorff M. A spatial scan statistic. Communications in Statistics-Theory and methods. 1997;26(6):1481-96.

26. Jung I, Kulldorff M, Klassen AC. A spatial scan statistic for ordinal data. Statistics in medicine. 2007;26(7):1594-607.

27. Pezzulo C, Nilsen K, Carioli A, Tejedor-Garavito N, Hanspal SE, Hilber T, et al. Geographical distribution of fertility rates in 70 low-income, lower-middle-income, and upper-middle-income countries, 2010–16: a subnational analysis of cross-sectional surveys. The Lancet Global Health. 2021;9(6):e802-e12.

28. Pezzulo C, Nilsen K, Carioli A, Tejedor Garavito N, Hanspal SE, Hilber T, et al. The geography of fertility rates in low and middle-income countries: a subnational analysis of cross-sectional surveys from 70 countries, 2010-16. The Lancet Global Health. 2021;9(6):E802-E12.

29. Kakujaha-Matundua O. Towards Identifying and estimating public expenditure on gender-based violence in Namibia. SOCIAL SCIENCES DIVISION (SSD). 2015;110.

30. Statistics UBO, MACRO I. Uganda Demographic and Health Survey. Kampala: Uganda Bureau of Statistics. 2011.

31. Acharya K, Paudel YR. Trend and sociodemographic correlates of cesarean section utilization in Nepal: evidence from demographic and health surveys 2006-2016. BioMed research international. 2021;2021.

32. Al Rifai RH. Trend of caesarean deliveries in Egypt and its associated factors: evidence from national surveys, 2005–2014. BMC pregnancy and childbirth. 2017;17(1):1-14.

33. Haider MR, Rahman MM, Moinuddin M, Rahman AE, Ahmed S, Khan MM. Ever-increasing Caesarean section and its economic burden in Bangladesh. PloS one. 2018;13(12):e0208623.

34. Zhang J, Zhang Y, Ma Y, Ke Y, Huo S, He L, et al. The associated factors of cesarean section during COVID-19 pandemic: a cross-sectional study in nine cities of China. Environmental health and preventive medicine. 2020;25(1):1-7.

35. Fesseha N, Getachew A, Hiluf M, Gebrehiwot Y, Bailey P. A national review of cesarean delivery in Ethiopia. International Journal of Gynecology & Obstetrics. 2011;115(1):106-11.

36. de Loenzien M, Schantz C, Luu BN, Dumont A. Magnitude and correlates of caesarean section in urban and rural areas: A multivariate study in Vietnam. PloS one. 2019;14(7):e0213129.

37. Rydahl E, Declercq E, Juhl M, Maimburg RD. Cesarean section on a rise—Does advanced maternal age explain the increase? A population register-based study. PloS one. 2019;14(1):e0210655.

38. Yaya S, Uthman OA, Amouzou A, Bishwajit G. Disparities in caesarean section prevalence and determinants across sub-Saharan Africa countries. Global health research and policy. 2018;3:1-9.

39. Mekonnen ZA, Lerebo WT, Gebrehiwot TG, Abadura SA. Multilevel analysis of individual and community level factors associated with institutional delivery in Ethiopia. BMC research notes. 2015;8(1):1-9.

40. Beyene MG, Zemedu TG, Gebregiorgis AH, Ruano AL, Bailey PE. Cesarean delivery rates, hospital readiness and quality of clinical management in Ethiopia: national results from two cross-sectional emergency obstetric and newborn care assessments. BMC pregnancy and childbirth. 2021;21:1-11.

41. Shibre G, Zegeye B, Ahinkorah BO, Keetile M, Yaya S. Magnitude and trends in socio-economic and geographic inequality in access to birth by cesarean section in Tanzania: evidence from five rounds of Tanzania demographic and health surveys (1996–2015). Archives of Public Health. 2020;78:1-10.

42. Logan RA, Wong WF, Villaire M, Daus G, Parnell TA, Willis E, et al. Health literacy: A necessary element for achieving health equity. NAM perspectives. 2015.

Table 3 **Socio-demographic character's of mother's who gave birth by cesarean section in Ethiopia Using 2019 MEDHS.**

Variable	Category	Cesarean section delivery			
		No	%	Yes	%
Age category	15-24	124	52	51	48
	25-34	2512	93.8	165	6.2
	35-49	1199	6.6	85	93.4
Sex of household	Male	4538	95	237	5
	Female	688	91.6	63	8.4

Place of residence	Urban	1229	89.9	138	11.1
	Rural	3998	96.1	162	3.9
Religion	Christian	3106	93.1	232	6.9
	Muslim	2120	96.8	69	3.2
Region	Tigray	346	93.3	25	6.7
	Afar	84	96.5	3	3.5
	Amhara	972	92.57	78	7.43
	Oromia	2120	95.9	90	4.1
	Somali	404	99.1	4	0.98
	Benshngul gumz	63	94.03	4	5.97
	SNNP	1054	95.38	51	4.62
	Gambella	24	96	1	4
	Hareri	15	88.24	2	11.76
	Addis Abeba	118	75.64	38	24.36
	Dredewa	25	83.3	5	16.7
Martital status	Union	2951	91.5	274	8.5
	Not union	276	91.1	27	8.9
Educational status	Cannot read and write	2875	97.1	86	2.9
	Primary education	1875	93.75	125	6.25
	secondary education	1832	97.23	52	2.77
	Higher education	362	90.5	38	9.5
Wealth index	Poorest	1300	98.4	21	1.6
	Poorer	1154	96.33	44	3.67
	Middle	997	95.5	47	4.5
	Richer	915	95.3	45	4.7
	Richest	861	85.8	144	14.3

Table 4 The multilevel analysis of associated factors of cesarean section in Ethiopia Using 2019 MEDHS.

Variable	Category	CS delivery		Bivariable		Model(null) 0	Model 1	Model 2	Model 3
		No	Yes	P-value	95%COR	ICC=4 4.1%	95%AOR	95%AOR	95%AOR
Age category	15-24	124	51		Reference		Reference		Reference
	25-34	2512	165	0.022	1.4(1.05-2.00)		1.9(1.3-3.07)		**1.98(1.3-3.1)**
	35-49	1199	85	0.003	1.73(1.21-2.47)		3.6(2.2-6.0)		**3.7(2.2-6.1)**
Sex of household	Male	4538	237		Reference		Reference		Reference
	Female	688	63	0.00	1.73(1.5-2.3)		1.8(1.1-2.9)		**1.9(1.2-3.1)**
Place of residence	Urban	1229	138		Reference			Reference	Reference
	Rural	3998	162	0.000	0.36(0.3-0.5)			0.3(0.14-0.5)	0.6(0.3-1.4)
Religion	Christian	3106	232		Reference				Reference
	Muslim	2120	69	0.000	0.4(0.3-0.57)		0.4(0.23-0.69)		**0.4(0.2-0.7)**
Region	Tigray	346	25		Reference		Reference	Reference	Reference
	Afar	84	3	0.153	0.36(0.1-1.5)			0.4(0.07-2.3)	2.4(0.3-23.7)
	Amhara	972	78	0.74	1.07(0.8-1.7)			1.4(0.5-3.7)	2.4(0.8-6.6)
	Oromia	2120	90	0.016	0.6(0.36-0.9)			0.5(0.2-1.4)	1.8(0.6-5.2)
	Somali	404	4	0.00	0.14(0.1-0.4)			0.13(0.03-0.6	0.6(0.05-4.33)
	Benshngul gumz	63	4	0.838	0.9(0.3-2.5)			0.9(0.2-4.2)	2.0(0.4-10.09)
	SNNP	1054	51	0.087	0.7(0.4-1.0)			1.0(0.4-2.7)	1.9(0.7-5.5)
	Gambella	24	1	0.556	0.5(0.05-4.3)			0.4(0.03-5.03)	0.3(0.02-6.6)

	Hareri	15	2	0.83	1.2(0.2-7.36)			1.0(0.1-9.6)	2.9(0.2-38.4)
	Addis Abeba	118	38	0.000	4.3(2.4-7.36)			2.9(0.9-8.9)	**3.6(1.1-11.9)**
	Dredewa	25	5	0.093	2.4(0.9-7.2)			2.5(0.6-11.02	**7.0(1.23-40.7)**
Marital status	Union	2951	274		Reference		Reference		Reference
	Not union	276	27	0.01	1.7(1.1-2.6)		1.37(0.72-2.6)		1.3(0.7-2.6)
Educational status	Cannot read and write	2875	86		Reference		Reference		Reference
	Primary education	1875	125	0.000	2.27(1.7-3.0)		1.68(1.07-2.6)		**1.6(1.07-2.7)**
	secondary education	1832	52	0.000	4.8(3.3-6.9)		2.3(1.3-4.12)		**2.4(1.3-4.25)**
	Higher education	362	38	0.000	7.9(5.2-12.6		2.2(1.07-4.4)		**2.06(1.03-4.2)**
Wealth index	Poorest	1300	21		Reference		Reference		Reference
	Poorer	1154	44	0.002	2.33(1.4-3.9)		1.4(0.68-3.05)		1.3(0.6-2.9)
	Middle	997	47	0.000	2.89(1.7-4.8)		0.79(0.4-1.7)		0.7(0.3-1.6)
	Richer	915	45	0.000	3.05(1.8-5.2)		0.9(0.4-1.9)		0.8(0.4-1.7)
	Richest	861	144	0.000	10.4(6.5-16.5)		1.3(0.5-2.6)		0.8(0.3-1.9)
Last birth by CS	Yes	43	25	0.000	1528.6(870-2684)				
	No	4842	281		Reference				
ANC Visit	No	11	1033		Reference		Reference		Reference
	Yes	272	2663	0.000	8.17(4.4-14.9		1.94(0.8-4.3)		1.9(0.8-4.2)
Types of birth	Single	325	5261		Reference		Reference		Reference

	Multiple	24	143	0.000	4.6(3.1-7.1)		8.01(3.35-19.6)		**8.1(3.4-19.74)**
Sex of New birth	Male	178	2791		Reference				Reference
	Female	171	2613	0.6	1.06(0.84-1.34)				
Place of delivery	Public health facility	283	2280		Reference		Reference		Reference
	Private hospital	66	243	0.013	1.68(1.2-2.5)		1.4(0.8-2.5)		1.3(0.6-2.4)

Key AOR= Adjusted odds ratio, COR crud odds ratio, ICC= Intra-Class Correlation, CS= Cesarean Section, ANC Antenatal care, SNNP South Nation Nationalities and Peoples,

 Click here to access/download;Figure;Fig1.tif

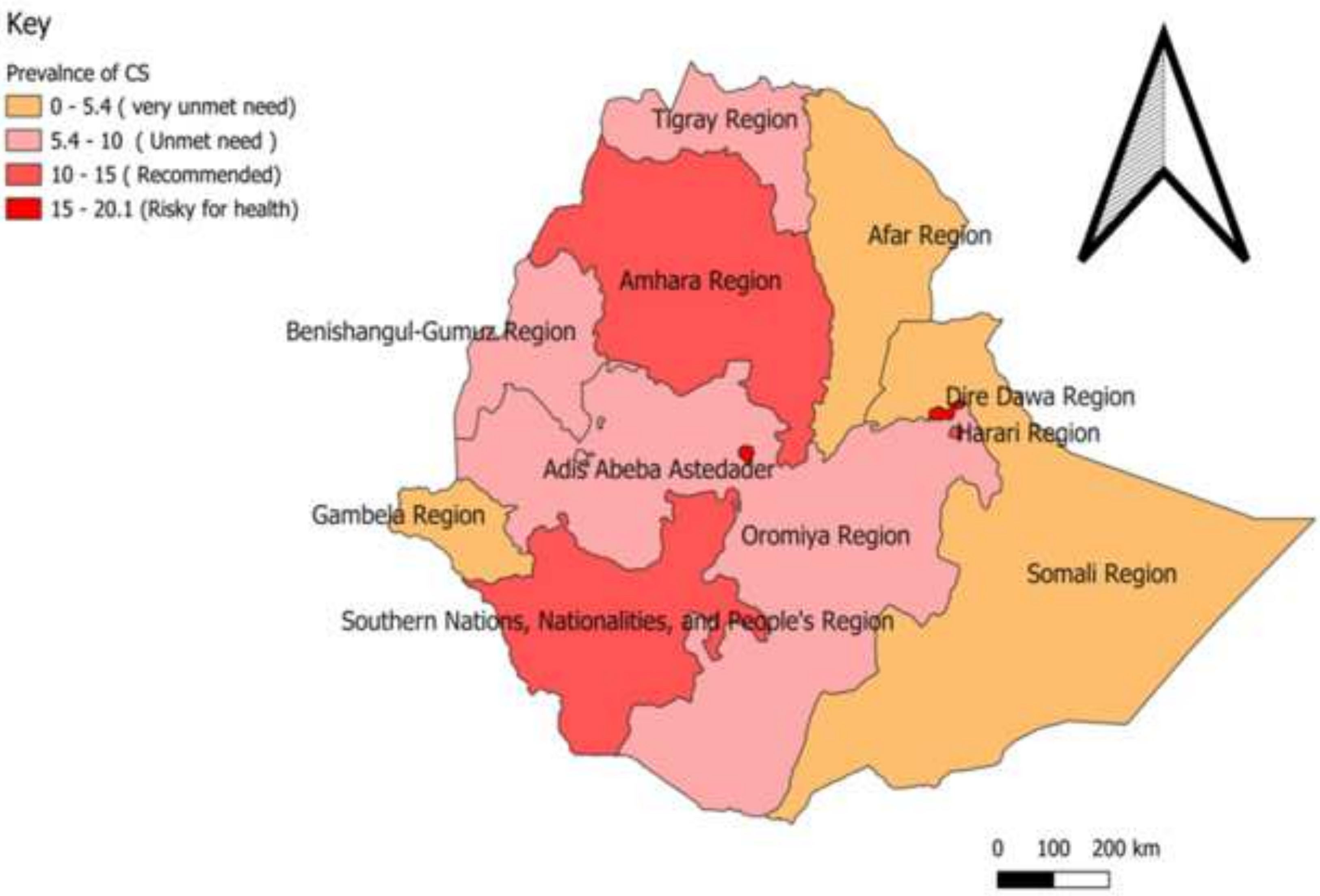

Fig.1. Spatial distribution of Cesceral section delivery in Ethiopia in 2019

Figure

Click here to access/download;Figure;fig2.tif

Spatial Autocorrelation Report

Moran's Index:	1.009090
z-score:	4.966507
p-value:	0.000001

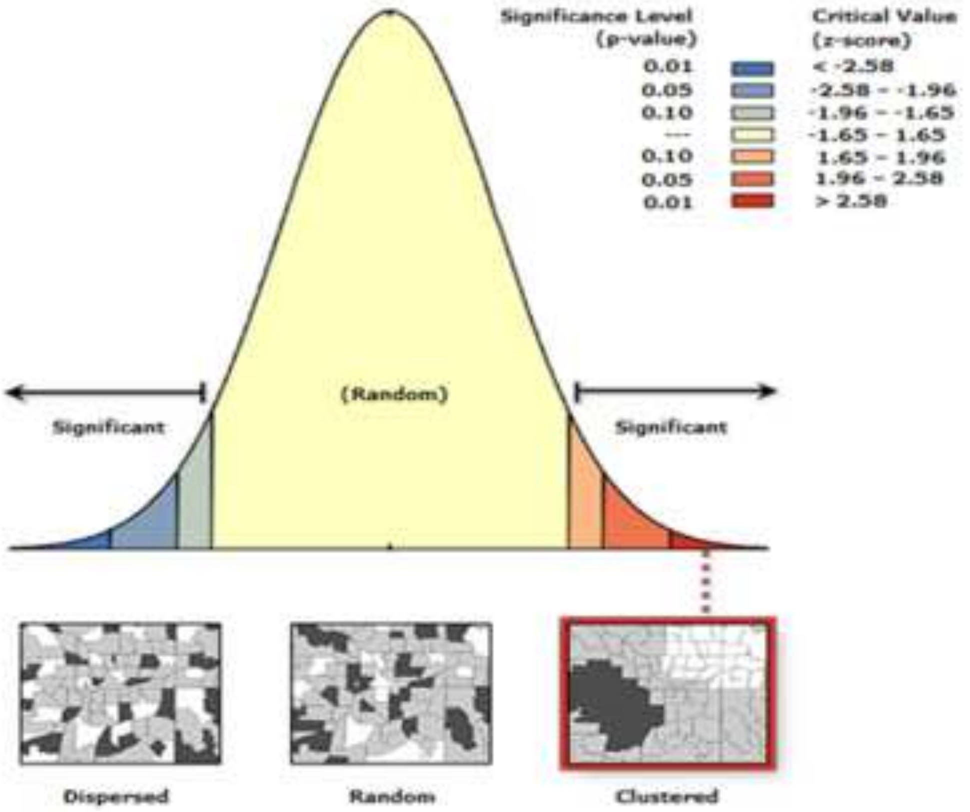

Given the z-score of 4.9665068324, there is a less than 1% likelihood that this clustered pattern could be the result of random chance.

Click here to access/download;Figure;Fig3.tif

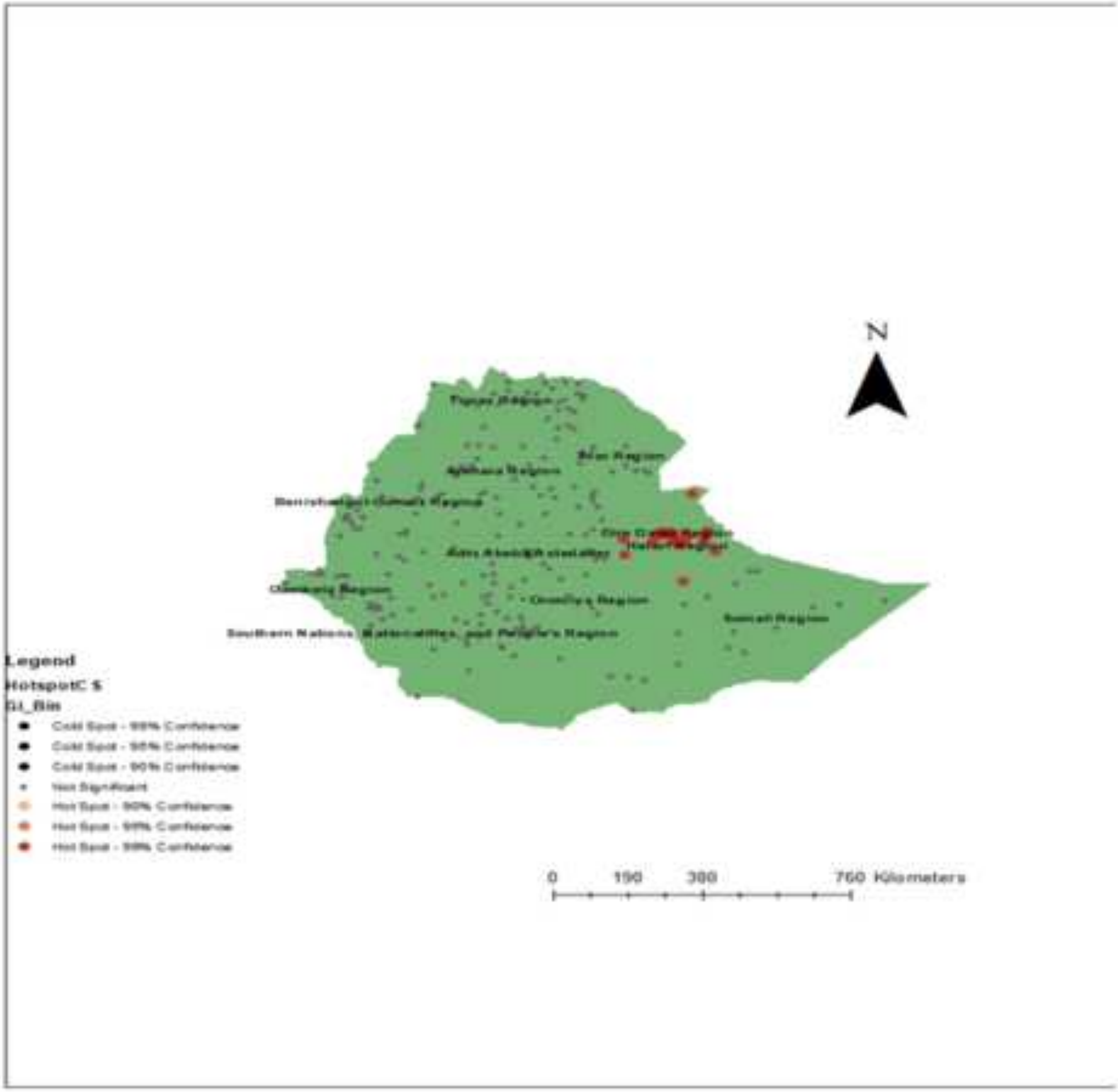

Click here to access/download
Supporting Information
Amendment CS.docx
