# Supplementary material for: Geospatial patterns, and individual and community levels factors of cesarean section deliveries in Ethiopia: A spatial and multilevel analysis
Source: PLoS One. 2024 Aug 27;19(8):e0306052. doi: 10.1371/journal.pone.0306052 (PMC11349178; doi:10.1371/journal.pone.0306052)
Supplement: Attachment — Submitted filename: PONE-D-23-40362_R1-AF.pdf [file pone.0306052.s003.pdf]

PLOS ONE

Geospatial Patterns, and Individual and Community Levels Factors of Cesarean Section Deliveries in Ethiopia: A Spatial and Multilevel Analysis

--Manuscript Draft--

**Manuscript Number:**	PONE-D-23-40362R1
**Article Type:**	Research Article
**Full Title:**	Geospatial Patterns, and Individual and Community Levels Factors of Cesarean Section Deliveries in Ethiopia: A Spatial and Multilevel Analysis
**Short Title:**	Geospatial Patterns, and Individual and Community Levels Factors of Cesarean Section Deliveries in Ethiopia:
**Corresponding Author:**	Abel Endawkie, MPH<br>Wollo University<br>Dessie, Amhara ETHIOPIA
**Keywords:**	Cesarean Section, Spatial Pattern, Multilevel, Ethiopia
**Abstract:**	Abstract<br><br>Background: Cesarean Section (CS) is the most popular surgery worldwide in obstetric care to save a mother's or the child's life. However spatial patterns and multilevel factors of CS delivery in Ethiopia are limited. Therefore, this study aimed to determine geospatial patterns and individual and community-level factors of CS deliveries in Ethiopia.<br>Method: A cross-sectional study using secondary data was conducted among 5,527 weighted samples of reproductive-age mothers using the 2019 Ethiopian mini demographic and health survey. Spatial and multilevel analyses were employed to determine spatial hotspots and colds pots, and multilevel factors of CS delivery in Ethiopia. Significance was declared at p-value <0.05 and adjusted odds ratio (AOR) with 95% confidence interval (CI) was reported.<br>Result: The prevalence of CS delivery in Ethiopia was 5.4% with 95%CI (4.6%, 6.4%). The spatial autocorrelation shows CS was clustered in Ethiopia (global Moran's index = 1.009, and p-value<0.001). Spatial hotspot indicates CS prevalent in Addis Ababa, Diredewa, Oromo, and Somali regions. The odds of CS delivery were higher among mothers aged 24-35: AOR = 1.98, 95% CI (1.3, 3.1), and 35-49: AOR=3.7, 95% CI(2.2, 6.1), live with female household heads: AOR =1.9, 95% CI (1.2, 3.1), mothers with primary AOR = 1.6, 95% CI (1.07, 2.7), secondary: AOR = 2.4, 95% CI (1.3, 4.25) and higher education: AOR = 2.06, 95% CI (1.03, 4.2), multiple births: AOR = 8.1, 95% CI (3.4, 19.74), mothers in Addis Ababa: AOR = 3.4, 95% CI (1.1, 11.9) and Diredewa: AOR = 7, 95% CI (1.23, 40.7) as compared to their counterparts.<br>Conclusion: In Ethiopia, CS deliveries remain below the recommended World Health Organization rates with distinct geospatial variation. Geographically, CS delivery is concentrated in urban areas like Addis Ababa and Diredewa, as well as in parts of the Somali and Oromia regions. Maternal age, female household head, Muslim religion, higher educational status, and multiple births at the individual level, and region at the community level were factors of CS delivery in Ethiopia. Therefore, efforts should be geared towards strategies and interventions to ensure fair access to CS delivery in line with WHO recommendations, especially in the regions where the CS delivery rate is low in Tigray, Amhara, Afar, and Benishangul Gumze regions.
**Order of Authors:**	Abel Endawkie, MPH
	Shimels Derso Kebede, MPH
	Natnael Kebede, MPH
	Mengistu Mera Mihiretu, MPH
	Ermias Bekele, MPH
	Kokeb Ayele, MPH
	Lakew Asmare, MPH

	Fekade Demeke Bayou, MPH
	Mastewal Arefaynie, MPH
	Yawkal Tsega, Masters of Health System and Management,
**Opposed Reviewers:**	
**Response to Reviewers:**	Authors' Responses to Editor and Reviewers Comments<br>"Geospatial Patterns, Prevalence, and Individual and Community Levels Factors of Cesarean Section Deliveries in Ethiopia: A Spatial and Multilevel Analysis"<br>Dear PlOS One Editors and Reviewers;<br>We are thankful for your constructive comments. We have looked at the comments and have revised our paper accordingly. We hope our paper improved as a result of incorporating the reviewer's and academic editor's comments and suggestions. Here are the authors' responses to the comments.<br>Please find for your kind consideration the following:<br>1.A revised manuscript with track changes.<br>2.A revised paper without tracked changes<br>3.A rebuttal letter that responds to each point raised by the academic editor and reviewer.<br>The point by point responses of authors are written by hoping these changes would meet with your favorable consideration, we are happy to hear if there are more comments and suggestions. Please do not hesitate to let us know if you have any questions.<br>Yours Sincerely<br>Mr. Abel Endawkie Correspondence Author<br>Department of Epidemiology and Biostatistics School of Public Health College of Medicine and Health Science Wollo University Dessie Ethiopia<br>Tel. 251935459310 Email address abelendawkie@gmail.com<br>We have tried our best to improve it accordingly:<br>Please revise the manuscript.<br>Journal requirements:<br>When submitting your revision, we need you to address these additional requirements.<br>1. Please ensure that your manuscript meets PLOS ONE's style requirements, including those for file naming. The PLOS ONE style templates can be found at https://journals.plos.org/plosone/s/file?id=wjVg/PLOSOne_formatting_sample_main_body.pdf and https://journals.plos.org/plosone/s/file?id=ba62/PLOSOne_formatting_sample_title_authors_affiliations.pdf<br>Response 1: We revised our manuscript based on Plos one submission guidelines (manuscript, title, table, figure and reference format).<br>2. We noticed you have some minor occurrence of overlapping text with the following previous publication(s), which needs to be addressed:<br>- https://www.researchsquare.com/article/rs-1386764/v1<br>- https://doi.org/10.1371/journal.pone.0282951<br>- https://bmcwomenshealth.biomedcentral.com/articles/10.1186/s12905-021-01484-1<br>Response 2: We would like to thank you kindly. After careful review, we made the necessary revisions and corrections.<br>3. We suggest you thoroughly copyedit your manuscript for language usage, spelling, and grammar. If you do not know anyone who can help you do this, you may wish to consider employing a professional scientific editing service.<br>Response 3: We are grateful to thank you for your kindness and response. We have tried to correct your the languge thoroughly.<br>3. Your ethics statement should only appear in the Methods section of your manuscript. If your ethics statement is written in any section besides the Methods, please delete it from any other section.<br>Response 2: We have prepared it.<br>Point-by-point response of Authors for editors' and reviewers' comment<br>Editor's Comments to the Authors<br>Additional Editor Comments:<br>The authors are requested to address the reviewers' comments carefully and in detail<br>Response: We are grateful to thank you for your kindness and response. We tried to correct it thoroughly.<br>Dear Editors: We would like to notify you that, for more appropriate understanding of the paper we have revised the title to "Geospatial Patterns, and Individual and

Community Levels Factors of Cesarean Section Deliveries in Ethiopia: A Spatial and Multilevel Analysis ”
Response: We are grateful to thank you for your kindness and response. We have tried to correct your concern and suggestion thoroughly.
Reviewer Comment to the Authors
Abstract section
Comment 1 Abstract result section: this is presented differently, better to use commen words.
Response 1: We sincerely appreciate your thoughtful feedback. After careful review, we made the necessary revisions and corrections.
Comment 2 Abstract conclusion section: The previous result no include the prevalence over time? So increase aspect is not clear please review.
Response 2: We extend our sincere gratitude for your insightful comment. We found it particularly valuable during the writing process of our work. We would like to sorry for making confuse in understanding. After careful review, we made the necessary revisions and corrections.
Comment 3: Abstract conclusion section: This sentence is not complete
Response 3: We sincerely appreciate your thoughtful comment. In response to your concern, since we found it particularly valuable during the writing process of our work and we would like to sorry for making confuse in understanding. After careful review, we made the necessary revisions and corrections.
Introduction section
Comment 4. Lesthan 5% people . It is not clear (people or pregnant women)
Response 4: We would like to thank you kindly for your comment. We had revised and corrected it thoroughly. Our interest is to say, according to the WHO statement, CS prevalence less than 5% among reproductive age mothers in the country or specific community indicates unmet need, whereas a prevalence of CS greater than 15% among reproductive age mothers might put the mother and new birth at risk.
Comment 5: population studies it is not clear?
Response 5: We would like to thank you kindly. We found it particularly valuable during the writing process of our work and we would like to sorry for making confuse in understanding. After careful review, we made the necessary revisions and corrections.
Comment 6: what is negative if CS rate lower infant death? It is not clear
Response 6: We express our gratitude for your insightful comment. We found it particularly valuable scientific plausibility of the paper and we would like to sorry for making confuse in understanding. After careful review, we made the necessary revisions and corrections.
Comment 7: Which WHO survey?
Response 7: First and foremost, we express our sincere gratitude for your intriguing idea and thoughtful questions. We have gained valuable insights from it. After careful review, we made the necessary revisions and corrections.
Comment 8: line 70 Explaine more.
Response 8: We sincerely appreciate your thoughtful comment. After careful review, we made the necessary revisions and corrections.
Comment 9: line 75 Start this in new paragraph given that is different topic.
Response 9: We express our sincere gratitude for your insightful comment. We have gained valuable insights from it. After careful review, we made the necessary revisions and corrections.
Comment 10. Line 77 Does it mean the rate of CS decreasing?
Response 10: We express our sincere appreciation for your insightful comment. We would like to sorry for making confuse in understanding. The rate of CS delivery in Ethiopia is increasing from 0.7 to 1.9 in 2000 to 2016 EDHS.
Commentm 11: line 80 write the numper in parenthesis after country name.
Response 11: We extend our sincere appreciation for your thoughtful comment. In response to your query, we made the necessary revisions and corrections..
Comment 12: introduce the mini EDHS data.
Response 12: We express our sincere appreciation for your insightful comment. Following your feedback, we have made revisions and incorporated your comment in manuscript.
Method section
Comment13: you have to explain it first MEDHS?
Response 13: First and foremost, we express our sincere gratitude for your intriguing idea and thoughtful questions. In response to your query, we have made revisions and incorporated your comment in manuscript.

Powered by Editorial Manager® and ProduXion Manager® from Aries Systems Corporation

	Comment 14: please explain more your sampling and sample size determination.<br>Response 14: We would like to thank you kindly for your deep comment. In response to your query, we have made revisions and incorporated your comment in manuscript. Please look the sampling procedure depiceted in figure 1 in detail<br>Comment 15. Line 120: no need of parenthesis<br>Response 15: We sincerely appreciate your thoughtful comment. The issue you raised is indeed intriguing. we have made revisions and incorporated your comment in manuscript.<br>Comment 16. Line 61: In which table please spacify<br>Response 16: We sincerely appreciate your thoughtful comment. We provided in table 2 and 3 Please look at table a 2 and 3<br>Comment 17: Line 131 please explaine more types of spatial statistics<br>Response 17: We sincerely appreciate your thoughtful comment. We have carefully reviewed and made the necessary corrections.<br>Comment 18. Please write the equation in new line<br>Response 18: We sincerely appreciate your thoughtful comment. We have carefully reviewed and made the necessary corrections.<br>Comment 19. Standar error never more than ±2<br>Response 19: We sincerely appreciate your thoughtful comment. Multicolinarity in logistic analysis is not estimated using variance inflaction factor (VIF). In this we consider the standard error of the variable. If the standard is below -2 and above 2 it indicates multiconiarity but it is does not mean that standard error never less than -2 or greater 2.<br>Result Section<br>Comment 20. Please edit more and please specify the delivery year before the DHS survey.<br>Response 20: We sincerely appreciate your thoughtful comment. We have carefully reviewed and made the necessary corrections.<br>Discusion Section<br>Comment 21. What was the previous CS delivery in Ethiopia please write<br>Response 21: We would like to thank you kindly for your deep comment. We have carefully reviewed and made the necessary corrections.<br>We feel all of you may understand our feelings and interests.<br>We are thankful for all!!
**Additional Information:**	
**Question**	**Response**
**Financial Disclosure**<br><br>Enter a financial disclosure statement that describes the sources of funding for the work included in this submission. Review the submission guidelines for detailed requirements. View published research articles from *PLOS ONE* for specific examples.<br><br>This statement is required for submission and **will appear in the published article** if the submission is accepted. Please make sure it is accurate.	The author(s) received no specific funding for this work.

**Funded studies**<br>Enter a statement with the following details:<br>• Initials of the authors who received each award<br>• Grant numbers awarded to each author<br>• The full name of each funder<br>• URL of each funder website<br>• Did the sponsors or funders play any role in the study design, data collection and analysis, decision to publish, or preparation of the manuscript?<br><br>Did you receive funding for this work?	
**Competing Interests**<br><br>Use the instructions below to enter a competing interest statement for this submission. On behalf of all authors, disclose any competing interests that could be perceived to bias this work—acknowledging all financial support and any other relevant financial or non-financial competing interests.<br><br>This statement is required for submission and **will appear in the published article** if the submission is accepted. Please make sure it is accurate and that any funding sources listed in your Funding Information later in the submission form are also declared in your Financial Disclosure statement.<br><br>View published research articles from *PLOS ONE* for specific examples.	The authors declare that they have no competing interests.

Powered by Editorial Manager® and ProduXion Manager® from Aries Systems Corporation

**NO authors have competing interests**<br><br>Enter: *The authors have declared that no competing interests exist.*<br><br>**Authors with competing interests**<br><br>Enter competing interest details beginning with this statement:<br><br>*I have read the journal's policy and the authors of this manuscript have the following competing interests: [insert competing interests here]*<br><br>* typeset	
**Ethics Statement**<br><br>Enter an ethics statement for this submission. This statement is required if the study involved:<br><br>• Human participants<br>• Human specimens or tissue<br>• Vertebrate animals or cephalopods<br>• Vertebrate embryos or tissues<br>• Field research<br><br>Write "N/A" if the submission does not require an ethics statement.<br><br>General guidance is provided below. Consult the submission guidelines for detailed instructions. **Make sure that all information entered here is included in the Methods section of the manuscript.**	No ethical approval was needed because we had used the demographic and health survey, which de-identifies all data before making it public, and the used mini DHS data sets are openly accessible. An authorization letter was requested to download the DHS data set and this was obtained from the Central Statistical Agency (CSA) after being requested at https://dhsprogram.com/. The dataset and all methods of this study were conducted according to the guidelines laid down in the Declaration of Helsinki principle and based on DHS research guidelines.

Powered by Editorial Manager® and ProduXion Manager® from Aries Systems Corporation

**Format for specific study types**<br><br>**Human Subject Research (involving human participants and/or tissue)**<br>• Give the name of the institutional review board or ethics committee that approved the study<br>• Include the approval number and/or a statement indicating approval of this research<br>• Indicate the form of consent obtained (written/oral) or the reason that consent was not obtained (e.g. the data were analyzed anonymously)<br><br>**Animal Research (involving vertebrate animals, embryos or tissues)**<br>• Provide the name of the Institutional Animal Care and Use Committee (IACUC) or other relevant ethics board that reviewed the study protocol, and indicate whether they approved this research or granted a formal waiver of ethical approval<br>• Include an approval number if one was obtained<br>• If the study involved *non-human primates*, add *additional details* about animal welfare and steps taken to ameliorate suffering<br>• If anesthesia, euthanasia, or any kind of animal sacrifice is part of the study, include briefly which substances and/or methods were applied<br><br>**Field Research**<br><br>Include the following details if this study involves the collection of plant, animal, or other materials from a natural setting:<br>• Field permit number<br>• Name of the institution or relevant body that granted permission	
**Data Availability**<br><br>Authors are required to make all data underlying the findings described fully available, without restriction, and from the time of publication. PLOS allows rare exceptions to address legal and ethical concerns. See the PLOS Data Policy and FAQ for detailed information.	Yes - all data are fully available without restriction

A Data Availability Statement describing where the data can be found is required at submission. Your answers to this question constitute the Data Availability Statement and **will be published in the article**, if accepted.<br><br>**Important:** Stating 'data available on request from the author' is not sufficient. If your data are only available upon request, select 'No' for the first question and explain your exceptional situation in the text box.<br><br>Do the authors confirm that all data underlying the findings described in their manuscript are fully available without restriction?	
**Describe where the data may be found in full sentences. If you are copying our sample text, replace any instances of XXX with the appropriate details.**<br><br>• If the data are **held or will be held in a public repository**, include URLs, accession numbers or DOIs. If this information will only be available after acceptance, indicate this by ticking the box below. For example: *All XXX files are available from the XXX database (accession number(s) XXX, XXX.).*<br>• If the data are all contained **within the manuscript and/or Supporting Information files**, enter the following: *All relevant data are within the manuscript and its Supporting Information files.*<br>• If neither of these applies but you are able to provide **details of access elsewhere**, with or without limitations, please do so. For example:<br><br>*Data cannot be shared publicly because of [XXX]. Data are available from the XXX Institutional Data Access / Ethics Committee (contact via XXX) for researchers who meet the criteria for access to confidential data.*<br><br>*The data underlying the results presented in the study are available from (include the name of the third party*	Data are available in a public, open access repository. Data for this study were sourced from Demographic and Health surveys (DHS) and available here: https://dhsprogram.com/data/available-datasets.cfm

and contact information or URL).<br>• This text is appropriate if the data are owned by a third party and authors do not have permission to share the data.<br><br>* typeset	
Additional data availability information:	Tick here if the URLs/accession numbers/DOIs will be available only after acceptance of the manuscript for publication so that we can ensure their inclusion before publication.

Powered by Editorial Manager® and ProduXion Manager® from Aries Systems Corporation

TO **Plose One**

Date: December 2023

Geospatial Patterns, Prevalence, and Individual and Community Levels Factors of Cesarean Section Deliveries in Ethiopia: A Spatial and Multilevel Analysis

Corresponding author Abel Endawkie

Dear Editor

The new manuscript, entitled as Geospatial Patterns, Prevalence, and Individual and Community Levels Factors of Cesarean Section Deliveries in Ethiopia: A Spatial and Multilevel Analysis, will make a substantial contribution to the science on the geospatial patterns, prevalence, and individual and community levels factors of cesarean section deliveries in Ethiopia: a spatial and multilevel analysis.

We choose, Plose one for this article, because it is a well-indexed, peer-reviewed, open-access journal with a strong contribution to the scientific community. The study bears no resemblance to previously published research. This paper, in its entirety or in parts, has not been published or submitted to elsewhere. It will serve as a resource for researchers interested in dealing with similar issues. As a result, policymakers and program designers will benefit from this research article. There are no conflicts of interest or opposing writers because the manuscript has been reviewed and approved by all authors.

"With Best Regards"

Abel Endawkie **Lecturers and researchers**

Email Adress abelendawkie@gmail.com

Manuscript Click here to access/download;Manuscript;Revised Manuscribt.docx

Geospatial Patterns, and Individual and Community Levels Factors of Cesarean Section Deliveries in Ethiopia: A Spatial and Multilevel Analysis

Abel Endawkie[1*] Shimels Derso Kebede[2], Natnael Kebede[3], Mengistu Mera Mihretu[4], Ermias Bekele[2], Kokeb Ayele[3], Lakew Asmare[1], Fekade Demeke Bayou[1], Mastewal Arefaynie[5], Yawkal Tsega[4]

Affiliation

[1]Department of Epidemiology and Biostatistics, School of Public Health, College of Medicine and Health Sciences, Wollo University, Dessie, Ethiopia.

2Department of Health Informatics, School of Public Health, College of Medicine and Health Sciences, Wollo University, Dessie, Ethiopia

3Department of Health Promotion, School of Public Health College of Medicine Health Sciences, Wollo University, Dessie, Ethiopia 4Department of Medical Nursing.

4Department of Health System and Management, School of Public Health, College of Medicine and Health Sciences, Wollo University, Dessie, Ethiopia

5Department of Reproductive and Family Health, School of Public Health, College of Medicine and Health Sciences, Wollo University, Dessie, Ethiopia

Corresponding author Abel Endawkie

Email Adress **abelendawkie@gmail.com**

Abstract

Background: Cesarean Section (CS) is the most popular surgery worldwide in obstetric care to save a mother's or the child's life. However spatial patterns and multilevel factors of CS delivery in Ethiopia are limited. Therefore, this study aimed to determine geospatial patterns and individual and community-level factors of CS deliveries in Ethiopia.

Method: A cross-sectional study using secondary data was conducted among 5,527 weighted samples of reproductive-age mothers using the 2019 Ethiopian mini demographic and health survey. Spatial and multilevel analyses were employed to determine spatial hotspots and colds pots, and multilevel factors of CS delivery in Ethiopia. Significance was declared at p-value <0.05 and adjusted odds ratio (AOR) with 95% confidence interval (CI) was reported.

Result: The prevalence of CS delivery in Ethiopia was 5.4% with 95%CI (4.6%, 6.4%). The spatial autocorrelation shows CS was clustered in Ethiopia (global Moran's index = 1.009, and p-value<0.001). Spatial hotspot indicates CS prevalent in Addis Ababa, Diredewa, Oromo, and Somali regions. The odds of CS delivery were higher among mothers aged 24-35: AOR = 1.98, 95% CI (1.3, 3.1), and 35-49: AOR=3.7, 95% CI(2.2, 6.1), live with female household heads: AOR =1.9, 95% CI (1.2, 3.1), mothers with primary AOR = 1.6, 95% CI (1.07, 2.7), secondary: AOR = 2.4, 95% CI (1.3, 4.25) and higher education: AOR = 2.06, 95% CI (1.03, 4.2), multiple births: AOR = 8.1, 95% CI (3.4, 19.74), mothers in Addis Ababa: AOR = 3.4, 95% CI (1.1, 11.9) and Diredewa: AOR = 7, 95% CI (1.23, 40.7) as compared to their counterparts.

Conclusion: In Ethiopia, CS deliveries remain below the recommended World Health Organization rates with distinct geospatial variation. Geographically, CS delivery is concentrated in urban areas like Addis Ababa and Diredewa, as well as in parts of the Somali and Oromia regions**.** Maternal age, female household head, Muslim religion, higher educational status, and multiple births at the individual level, and region at the community level were factors of CS delivery in Ethiopia. Therefore, efforts should be geared towards strategies and interventions to ensure fair access to CS delivery in line with WHO recommendations, especially in the regions where the CS delivery rate is low in Tigray, Amhara, Afar, and Benishangul Gumze regions.

Keywords: Cesarean Section, Spatial Pattern, Multilevel, Ethiopia

Introduction

Cesarean section (CS) is the most usually lifesaving surgical procedure in obstetric care. It is the birth of a fetus through a surgical incision made in the uterine and abdominal walls of a pregnant woman. Preventing maternal and newborn morbidity and mortality rates is the primary purpose of conducting it, either for fetal or maternal reasons [1]. CS more frequently than ever before, and this trend is expected to continue in the next ten years [2, 3], even though there is still a significant lack of safe CS in resource-constrained nations.

Since 1985, the World Health Organization (WHO) recommends that CS prevalence should be between 10-15% of all births among reproductive age mothers in the country or specific community [4]. According to the WHO statement, CS prevalence less than 5% among reproductive age mothers in the country or specific community indicates unmet need, whereas a prevalence of CS greater than 15% among reproductive age mothers might put the mother and new birth at risk [5].

The average global CS prevalence according to recent statistics from 154 nations is 21.1% and it ranges from 8.2% in the least developed to 27.2% in the more developed countries [6]. The prevalence of CS delivery in African countries Ghana (13%) [7], Nigeria (2.1%) [8], and Mozambique (4.7%) [9]. The prevalence of CS delivery in the 2000 and 2016 Ethiopia Demographic and Health Surveys (EDHS) were 0.7 percent and 1.9 percent respectively [10, 11]. A population-based study, showed that CS rates greater than 10% are not linked to lower ~~the~~ rates of maternal and newborn mortality [12]. The number of CS deliveries per 100 births, or the CS rate, has a significant linear connection with infant death, according to another ecological study [13]. Despite all of these facts, the ~~medically non-indicated~~ CS section has seen a large increase in practice in recent years [14]. A research conducted in Ethiopia showed that a rise in CS has been associated with both medically and non-medically indicated factors, such as the kind of health facility, socio-demographic traits, and maternal health of mothers [15, 16]. Study conducted in Ethiopia indicates that CS delivery increased risk of infection, a longer healing period, significant bleeding, and an increase in iatrogenic injuries, breathing difficulties, and even death [17, 18]. Different studies reported different prevalence and associated factors for cesarean section delivery in Ethiopia [3, 6, 19, 20].

Mother's age, birth order, first-parity births, Antenatal Care (ANC) visits, urban residence, higher education levels, and higher economic statuses were factors of CS delivery among mothers in

Ethiopia [3, 6, 19-21]. These studies used small sample sizes which was not representative and were prone to selection bias [3, 6, 19, 20]. The 2019 mini EDHS is sufficient to provide nationwide data to conduct analysis of individual and community level factors of CS delivery in Ethiopia Moreover, in Ethiopia spatial pattern of caesarean section delivery using national representative data of 2019 mini Ethiopian Demographic and Health Survey mini (EDHS) is limited. Therefore, this study aimed to determine geospatial patterns, and individual and community levels factors of cesarean section deliveries in Ethiopia using nationwide 2019 mini EDHS data.

Method

Study Setting

The study was done in Ethiopia which has a diverse population. It is bordered on the west by Sudan, on the east by Somalia and Djibouti, on the north by Eritrea, and on the south by Kenya. The country has a total area of 1,112,000 square kilometers Ethiopia is divided into eleven regions and two municipal governments. Tigray, Afar, Amhara, Oromiya, Somali, Benishangul-Gumuz, Southern Nations Nationalities and People (SNNP), Gambela, Sidama, South Western, and Harari are among the regions involved. Addis Ababa and Dire Dawa are administrative cities. Data were accessed from their URL: www.dhsprogram.com by contacting them through personal accounts after justifying the reason for requesting the data [22].

Study Design and Source Population

The research was conducted using secondary data from a cross-sectional study used in 2019 mini EDHS using children's records data. The source population was reproductive age mothers (15-49) who gave birth 5 years before the survey.

Sample Size and Sampling Methods

A total of 5,526.93 ~ 5,527 weighted reproductive mothers were used in this analysis as a sample that was aimed to represent all of the country's regions and administrative cities from 5,753 mothers used in mini EDHS 2019. The sample was stratified and selected in two stages. In the first stage, stratification was conducted by region, and then each region was stratified as urban and rural. The sample size was then allocated using a probability-proportional allocation method. There were 305 enumeration areas (EAs) chosen for the 2019 mini EDHS (Fig1).

Figure 1. Sampling and exclusion procedures to identify the final sample size of the study

Variable Measurement

Cesarean section delivery categorized as Yes"1" and No "0" was the dependent variable and the individual-level factors included: age, sex of household, religion, wealth index, marital status, educational status, ANC visit, types of birth, place of delivery, and community-level factors: region, and residence were considered as the independent variable.

Data Processing and Analysis

Data used were extracted, cleaned, coded, and analyzed using STATA version 17 statistical software. Sample weights were done before further analysis to compensate for the unequal probability of selection between the strata that were geographically defined and for non-responses. Descriptive statistics were calculated and presented using tables, figures, and narratives.

Spatial Analysis:

To execute the spatial analysis, the weighted proportions of outcome variables (CS delivery) with cluster number were computed in STATA version 17 and the spatial analysis was computed using QGIS version 3.28.15 and ArcGIS version 10.7.1 statistical software.

Spatial Distribution: The descriptive spatial analysis was conducted to determine distribution of CS delivery in Ethiopia using QGIS version 3.28.15.

Spatial Autocorrelation Analysis

The spatial autocorrelation (Global Moran's I) statistic measure is used to assess whether CS delivery is dispersed, clustered, or randomly distributed in Ethiopia. Moran's I value close to − 1 shows dispersed CS delivery, close to + 1 shows clustered, and if Moran's I value zero shows randomly distributed [23]. In this study global spatial autocorrelation indicate CS delivery is clustered at p-value 0.0000 and Global Moran's I (MI) =1.0009.

Hotspot Analysis: The spatial hotspot analysis was conducted using Getis-Ord Gi* hot spot analysis of ArcGIS version 10.7.1 to examine where spatial clusters of CS delivery occur on the landscape by identifying areas with statistically significant clustering of relative high values (hot spots) or relative low values (cold spots). This measure indicates the presence or absence of significant spatial clustering, with the null hypothesis being that there is no difference in characteristics between a unit and its spatial neighbors [24-27].

Spatial Interpolation: Spatial interpolation was used to estimate the proportion of CS delivery distribution in un-sampled areas, based on observed measurements in the sampled enumeration

areas. Although there are different deterministic and geostatistical interpolation methods available, we opted to use the Ordinary Kriging spatial interpolation method since it incorporates spatial autocorrelation statistically optimizes the weight, and has low residual and mean square error [28].

Multilevel Analysis: Multilevel analysis was conducted after checking the eligibility of the data for multilevel analysis by using the intra-cluster correction coefficient (ICC). Since ICC is greater than 10% (ICC=44.1%) calculated as below [29].

$$ICC = \frac{\delta 2}{\delta 2 + \pi 2/3} \text{ --- } (1)$$

Where δ2 indicates the estimated variance of clusters, multi-level regression was conducted to assess factors related to individual and community-level characteristics of cesarean section. Multilevel analysis was considered appropriate due to the hierarchical nature of the DHS data as well as the estimation of individual and community-level effects. The log of the probability of a cesarean section was modeled using a two-level multilevel model as follows. $Log[\frac{\pi ij}{1-\pi ij}]$ =β0+β1Xij+ B2 Zij+μj+ eij, Where i and j are individual level and community level[30] units respectively; X and Z refer to individual and community level variables respectively; πij is the probability of cesarean section for the ith mothers in the jth community; β's indicates the fixed coefficients. (B0) is the intercept, the effect on the probability of cesarean section in the absence of influencing factors; and μj showed the random effect (the effect of the community on the cesarean section of the jth community) and eij showed random errors at the individual level. By assuming each community had a different intercept (B0) and fixed coefficient (β), the clustered data nature and intra and inter-community variations were taken into account. Data organized into, analyses of cesarean section yes/no which has the binary response and runs mixed effect multi-level logistic regression model by considering two levels (individual and community levels). During analysis first, bi-variable multilevel logistic regression was fitted, and variables with p value less than 0.2 at model I and model II were selected to develop the 3rd model (the final model). The analysis was done in four models. The first model was, model-0 (empty model or null model), the second model was, model-I (analyzing only individual-level variables), the 3rd model was, model-II (analyzing only community-level variables), the last model, model-III (analyzing both community-level and individual-level variables based on the cut of the point). Variables with p value less than 0.05 at model-III were significantly associated with cesarean section. The random

effects (variations) were measured by using ICC (model-0), Median Odds Ratio (MOR) in (model-I and II), and Proportional Change in Variance (PCV) was measured to show variation between clusters. ICC shows the variation in cesarean section among mothers due to community characteristics. MOR is the median value of the odds ratio between the area at the highest risk and the area at the lowest risk when randomly picking out two areas and it was calculated as follows MOR=exp ($\sqrt{2 + \delta 2 + 0.6745}$. PCV measures the total variation attributed to individual-level variables and area-level variables in the final model (model-III). It is calculated as below.

PCV= [δ2 of null model−δ2 of each model)/δ2 of null model], where δ2 of the null model is used as a reference. Multicollinearity was checked among explanatory variables by using standard error at a cut of point±2 and there is no Multicollinearity since the standard errors were between±2. The appropriateness of the mixed model (model fitness) is checked using Inter-Class Correlation and model selection was based on Log Likelihood Ratio (LLR) or Akakian information criteria AIC and Bayesian information criteria (BIC).

Ethical Approval: No ethical approval was needed because we had used the demographic and health survey, which de-identifies all data before making it public, and the used mini DHS data sets are openly accessible. An authorization letter was requested to download the DHS data set and this was obtained from the Central Statistical Agency (CSA) after being requested at https://dhsprogram.com/. The dataset and all methods of this study were conducted according to the guidelines laid down in the Declaration of Helsinki principle and based on DHS research guidelines.

Result

Socio-demographic characters of reproductive age mothers who gave birth 5 years before the survey

A total of, 5,527 weighted reproductive-age mothers [15–49] were included in the analysis. Among 205 reproductive-age mothers, 48% of mothers in the age group of 15-24 have delivered via cesarean section, from 2, 961 reproductive-age mothers, 2.9% of mothers who cannot read and write have delivered via cesarean section. Among 4160 reproductive-age mothers, 11.1% of mothers residing in rural areas had delivered via cesarean section (Table 1).

Table 1: Socio-demographic characters of reproductive age mothers who gave birth 5 years before the survey in Ethiopia, 2019 mini EDHS (weighted).

Variable	Category	Cesarean section delivery			
		No	%	Yes	%
Age category	15-24	124	52	51	48
	25-34	2512	93.8	165	6.2
	35-49	1199	6.6	85	93.4
Sex of household	Male	4538	95	237	5
	Female	688	91.6	63	8.4
Place of residence	Urban	1229	89.9	138	11.1
	Rural	3998	96.1	162	3.9
Religion	Christian	3106	93.1	232	6.9
	Muslim	2120	96.8	69	3.2
Region	Tigray	346	93.3	25	6.7
	Afar	84	96.5	3	3.5
	Amhara	972	92.57	78	7.43
	Oromia	2120	95.9	90	4.1
	Somali	404	99.1	4	0.98
	Benishngul Gumz	63	94.03	4	5.97
	SNNP	1054	95.38	51	4.62
	Gambella	24	96	1	4
	Hareri	15	88.24	2	11.76
	Addis Abeba	118	75.64	38	24.36
	Diredewa	25	83.3	5	16.7
Marital status	Union	2951	91.5	274	8.5
	Not union	276	91.1	27	8.9
Educational status	Cannot read and write	2875	97.1	86	2.9
	Primary education	1875	93.75	125	6.25
	secondary education	1832	97.23	52	2.77
	Higher education	362	90.5	38	9.5
Wealth index	Poorest	1300	98.4	21	1.6
	Poorer	1154	96.33	44	3.67

	Middle	997	95.5	47	4.5
	Richer	915	95.3	45	4.7
	Richest	861	85.8	144	14.3

Maternal health service utilization of reproductive age mothers who gave birth 5 years before the survey

Regarding utilization of maternal health services utilization, 234 (8[illegible]%) of mothers who had ANC visits delivered via cesarean section, and 270 (10.8) of mothers who delivered in private hospitals delivered via cesarean section (Table 2).

Table **2**: The maternal health service utilization of reproductive age mothers who gave birth 5 years before the survey in Ethiopia Using 2019 mini EDHS (weighted).

Variable	Category	cesarean section delivery			
		No	%	Yes	%
Last delivery by previous CS	Yes	4653	99.6	20	0.4
	No	36	13.3	234	86.7
ANC Visit	No	993	98.9	11	1.1
	Yes	2689	91.99	234	8.01
Types of birth	Single	5109	94.96	271	5.04
	Multiple	118	80.3	29	19.7
Sex of New Birth	Male	2692	94.7	150	5.3
	Female	2535	94.4	150	5.6
Place of delivery	Public health facility	2235	89.2	270	10.8
	Private hospital	149	83.2	30	16.8

Spatial distribution, cluster (hotspot/cold spot) and interpolation of CS delivery in Ethiopia

The descriptive spatial analysis showed that the prevalence of CS delivery in Ethiopia was highest in Addis Abeba, and Diredewa, and the low cluster (unmet need) in Afar, Tigray, and Benishangul Gumz regions (Fig 2).

Figure 2: Spatial distribution of CS delivery in Ethiopia

Spatial autocorrelation showed that there is a clustering of CS delivery [MI= 1.009, Z score =4.99, and p-value <0.001] (Fig 3).

Figure 3: Spatial autocorrelation analysis of CS delivery in Ethiopia

The Hotspot analysis showed that CS delivery was hotspots [high prevalence] in Diredewa, Harerie, Somali, and Oromia regions, and cold spots [low prevalence] of CS delivery in Tigray, Amhara, Afar, and Benishangul Gumz region (Fig 4).

Figure 4: Spatial hotspot analysis of CS delivery in Ethiopia

The spatial interpolation of CS delivery for locations where data were not obtained has been estimated using the standard Kriging technique. The highest estimated prevalence of CS delivery (red shaded) was observed in Addis Ababa, Diredewa and parts of Somalia regions (Fig 5).

Figure 5: Spatial interpolation analysis of CS delivery in Ethiopia

Prevalence of Cesarean Section Delivery in Ethiopia

The prevalence of cesarean section delivery was 5.4% (95% CI (4.6%-6.4%)) in Ethiopia.

Individual and Community-Level Factors of Cesarean Section Delivery in Ethiopia

In the final model (model-III) maternal age, sex of the household, religion, educational status, types of birth from individual-level variables, and the region from community-level variables were associated with cesarean section delivery (Table 3). The odds of CS delivery were 1.98 and 3.7 times more among mothers whose ages are in the age group 25-34 and 35-49 as compared to mothers in the age group15-24 [AOR=1.98, 95% CI=(1.3, 3.1)] and [AOR=3.7, 95% CI=(2.2, 6.1)] respectively. The odds of CS delivery were 1.9 times higher among participants who lived in female head households as compared to male head households [AOR=1.9, 95% CI=(1.2, 3.1)]. The odds of CS delivery were 60% less likely among participants who are Muslim religion followers as compared to Christian religion followers [AOR=0.4, 95% CI= (0.2, 0.7)]. Mothers who attended primary education, secondary education, and higher education were 1.6, 2.4, and 2.06 times more likely to have CS delivery as compared with those ~~not attended~~ school [AOR=1.6, 95% CI=(01.07, 2.7)], [AOR=2.4, 95% CI=(1.3, 4.25)], and [AOR=2.06, 95% CI (1.03, 4.2)] respectively. The odds of CS delivery were 8.1 times higher for multiple births as compared with single births [AOR=8.1, 95% CI=(3.4, 19.74)]. Mothers who were living in Addis Ababa and Dire Dewa were 3.6 and 7 times more likely to have cesarean section delivery as compared to mothers who live in the Tigray region [AOR=3.4 95% CI=(1.1, 11.9)], and [AOR=7, 95%CI=(1.23, 40.7)] respectively (Table 3).

Table 3: The multilevel analysis of associated factors of cesarean section delivery among reproductive age mothers who gave birth 5 years before the survey in Ethiopia, 2019 mini EDHS (weighted).

Variable	Category	CS delivery		Bivariable		Model 0	Model 1	Model 2	Model 3
		No	Yes	P-value	95%COR	ICC=44.1%	95%AOR	95%AOR	95%AOR
Age category	15-24	124	51		Reference		Reference		Reference
	25-34	2512	165	0.022	1.4(1.05-2.00)		1.9(1.3-3.07)		1.98(1.3-3.1)*
	35-49	1199	85	0.003	1.73(1.21-2.47)		3.6(2.2-6.0)		3.7(2.2-6.1)*
Sex of household	Male	4538	237		Reference		Reference		Reference
	Female	688	63	0.00	1.73(1.5-2.3)		1.8(1.1-2.9)		1.9(1.2-3.1)*
Place of residence	Urban	1229	138		Reference			Reference	Reference
	Rural	3998	162	0.000	0.36(0.3-0.5)			0.3(0.14-0.5)	0.6(0.3-1.4)
Religion	Christian	3106	232		Reference				Reference
	Muslim	2120	69	0.000	0.4(0.3-0.57)		0.4(0.23-0.69)		0.4(0.2-0.7)
Region	Tigray	346	25		Reference		Reference	Reference	Reference
	Afar	84	3	0.153	0.36(0.1-1.5)			0.4(0.07-2.3)	2.4(0.3-23.7)
	Amhara	972	78	0.74	1.07(0.8-1.7)			1.4(0.5-3.7)	2.4(0.8-6.6)
	Oromia	2120	90	0.016	0.6(0.36-0.9)			0.5(0.2-1.4)	1.8(0.6-5.2)
	Somali	404	4	0.00	0.14(0.1-0.4)			0.13(0.03-0.6	0.6(0.05-4.3)
	Benshngul	63	4	0.838	0.9(0.3-2.5)			0.9(0.2-4.2)	2.0(0.4-10.1)
	SNNP	1054	51	0.087	0.7(0.4-1.0)			1.0(0.4-2.7)	1.9(0.7-5.5)
	Gambella	24	1	0.556	0.5(0.05-4.3)			0.4(0.03-5)	0.3(0.02-6.6)
	Hareri	15	2	0.83	1.2(0.2-7.36)			1(0.1-9.6)	2.9(0.2-38.4)
	Addis Abeba	118	38	0.000	4.3(2.4-7.36)			2.9(0.9-8.9)	3.6(1.1-11.9)*
	Dredewa	25	5	0.093	2.4(0.9-7.2)			2.5(0.6-11.02	7.0(1.2-40.7)*
Marital status	Union	2951	274		Reference		Reference		Reference
	Not union	276	27	0.01	1.7(1.1-2.6)		1.37(0.72-2.6)		1.3(0.7-2.6)
Educational status	Cannot read and write	2875	86		Reference		Reference		Reference

	Primary education	1875	125	0.000	2.27(1.7-3.0)		1.68(1.07-2.6)		1.6(1.07-2.7)*
	secondary education	1832	52	0.000	4.8(3.3-6.9)		2.3(1.3-4.12)		2.4(1.3-4.25)*
	Higher education	362	38	0.000	7.9(5.2-12.6		2.2(1.07-4.4)		2.06(1.1-4.2)*
Wealth index	Poorest	1300	21		Reference		Reference		Reference
	Poorer	1154	44	0.002	2.33(1.4-3.9)		1.4(0.68-3.05)		1.3(0.6-2.9)
	Middle	997	47	0.000	2.89(1.7-4.8)		0.79(0.4-1.7)		0.7(0.3-1.6)
	Richer	915	45	0.000	3.05(1.8-5.2)		0.9(0.4-1.9)		0.8(0.4-1.7)
	Richest	861	144	0.000	10.4(6.5-16.5)		1.3(0.5-2.6)		0.8(0.3-1.9)
Last birth by CS	Yes	43	25	0.000	1528.6(870-2684)				
	No	4842	281		Reference				
ANC Visit	No	11	1033		Reference		Reference		Reference
	Yes	272	2663	0.000	8.17(4.4-14.9)		1.94(0.8-4.3)		1.9(0.8-4.2)
Types of birth	Single	325	5261		Reference		Reference		Reference
	Multiple	24	143	0.000	4.6(3.1-7.1)		8.01(3.4-19.6)		8.1(3.4-19.7)*
Sex of New Birth	Male	178	2791		Reference				Reference
	Female	171	2613	0.6	1.06(0.84-1.34)				
Place of delivery	Public health facility	283	2280		Reference		Reference		Reference
	Private hospital	66	243	0.013	1.68(1.2-2.5)		1.4(0.8-2.5)		1.3(0.6-2.4)

Key *= significant at 95% confidence interval, AOR= Adjusted odds ratio, COR= crude odds ratio, ICC= Intra-Class Correlation, CS= Cesarean Section, ANC= Antenatal care, SNNP= South Nation Nationalities and Peoples

Random effects (measures of variation)

Cesarean section delivery varies significantly across each cluster. ICC indicated that 44.1% of the variation in CS delivery among mothers was attributed to community-level factors. PCV in the final model showed that 29.1% of the variation in CS delivery across communities was explained. The median odds ratio confirmed that the variation in CS delivery was influenced by community-level factors. In the initial model (model zero), the median odds ratio for CS delivery was 9.2, indicating significant variation between communities (9.2 times higher than the reference or MOR=1). However, when all factors were added to the final model, the unexplained community variation decreased to an MOR of 7.9. This suggests that even after considering all factors, the effects of clustering remain statistically significant in the full models (Table 4).

Table 4 Model fitness and selection

Model	ICC	Variance	PCV	MOR	LLR	AIC	BIC
Model 0	44.1%	2.3	Reference	9.2	-1202	2408	2421
Model 1		1.67	27.39%	8.01	-639	1423	1514
Model 2	32.07%	1.7	26.09%	8.08	-983	1993	2079
Model 3	31.2%	1.63	29.1%	7.9	-632	1320	1479

Discussion

This study used the 2019 mini EDHS data to analyze the geospatial pattern and individual and community-level factors of CS delivery in Ethiopia. The finding of this study indicates that CS prevalence was 5.44% in Ethiopia. The finding is similar to study conducted in Mozambique (4.7%) [9], and Uganda (5.22%) [31]. However, this prevalence is less than the research conducted in Nepal (9%) [32], Egypt (40.1%) [33], Bangladesh (6[illegible]3%) [34], and China (37%) [35]. This is slightly increased from the previous EDHS report in 2011 (0.9%) [36], and 2016 (1.9%). This indicates that CS availability has slightly improved, however, it is still below the WHO recommendation which indicates unmet need as per the WHO statement [4, 5]. The unmeet rate of CS delivery and geographical disparities in the region of Ethiopia in the current study could be attributed to many factors, including inadequate and/or sparse availability of emergency obstetrics services across the nation, inadequate equipment and medication in obstetric emergency centers, a shortage of skilled birth attendants and services, long travel distances, and unsuitable terrain that

could provide a substantial geographic barrier to emergency obstetric treatment [37]. Related to factors, maternal age, sex of the household, religion, educational status, types of birth from individual level variable and, region from community-level variable had a statistical association with CS delivery. The probability of having cesarean section delivery was high among participants who are in the age group 25-34 and 35-49 as compared to mothers in the age group. This finding is supported the by study in Vietnam [38], Egypt [33], and Mozambique [9]. The reason might be that the risk of delivery problems as well as the chance of premature birth and infant death increased as mothers got older, excessive bleeding during labor, prolonged labor lasting more than 20 hours, and dysfunctional labor that does not progress to the next stage. Furthermore, diabetes and hypertension during pregnancy were more common in older pregnant mothers; this may lead to increased delivery by CS [39]. The odds of mothers who live with a female household head were more likely to deliver by CS than mothers who live with a male household head. There is no evidence to support this finding [19-21, 35, 40]. Muslim religion-follower mothers were less likely to deliver by CS delivery than Orthodox religion-follower mothers. This finding is supported by the study conducted in Nigeria [8]. Mothers who were living in Addis Ababa and Dire Dewa were; more likely to have CS delivery as compared to mothers who lived in the Tigray region [41]. The odds of CS delivery among mothers who had completed primary, secondary, or higher education were more likely than mothers who had not attended education. This finding is supported by the study conducted by global, and national representatives of 150 countries using secondary data [2, 35, 38, 42-44]. The possible reason for a higher degree of education enhances the possibility of CS delivery might be mothers who have received education aware of the costs and benefits of using maternity services and also have more confidence and self-reliance for any decision provided by a healthcare provider, which in turn, may raise the chance that could get CS service, besides, evidence from health literature demonstrate[illegible]e fact that health literacy is linked to educational achievement could lead to believe that highly educated mothers are more likely to seek health services such as CS delivery than their no educated counterparts [45]. In this finding, multiple births (twin pregnancies) were significant factors for CS delivery. This study is supported by a study conducted in Egypt [33] and Nigeria [8]. The possible reason for this could be multiple births (twin pregnancies) have been linked to obstetric complications like preterm labor, premature rupture of membranes, malposition, and male presentation of the fetus. It also increased risks of complications to the mother like gestational diabetes, gestational hypertension, preeclampsia, and

intrahepatic cholestasis, all of which may increase the likelihood of giving birth via CS delivery. The strength of research was using nationally representative datasets, which gives sufficient statistical power and the study estimates completed after the data had been weighted to allow for probability sampling and nonresponse to make it representative at national and regional levels. The limitation of this study was some factors that affecting CS delivery, like birth weight, maternal weight and height to generate BMI were incomplete in the 2019 mini EDHS data.

Conclusion

In Ethiopia, CS deliveries remain below the recommended World Health Organization rates with distinct geospatial variation. Geographically, CS prevalence is concentrated in urban areas like Addis Ababa and Diredewa, as well as in parts of the Somali and Oromia regions**.** Maternal age, female household head, Muslim religion, higher educational status, and multiple births at the individual level, and region at the community level were factors of CS delivery in Ethiopia. Therefore, efforts should be geared towards tailoring strategies and interventions to ensure fair access to CS delivery lines with WHO recommendations, especially in the region where CS delivery rates are low in Tigray, Amhara, Afar, and Benishangul Gumze regions.

Acknowledgments: The authors are sincerely grateful to the Demographic Health Survey (DHS) program for allowing us to use the Ethiopian mini DHS dataset through their archives (archive@dhsprogram.com).

List of Abbreviation: AIC: Akakian information Criteria, AOR: Adjusted odds ratio, ANC: Antenatal care, BIC: Bayesian information Criteria, COR: Crud odds ratio, EA: Enumeration Area, ICC: Intra-Class Correlation, MEDHS: Mini Demographic and Health Survey, MOR: Median Odds Ratio, PCV: Proportional Changes of Variance, SNNP: South Nation

Reference

1. Jauniaux, E. and W. Grobman, *Textbook of caesarean section*. 2016: Oxford University Press.
2. Boerma, T., et al., *Global epidemiology of use of and disparities in caesarean sections.* The Lancet, 2018. **392**(10155): p. 1341-1348.

3. Batieha, A., et al., *Cesarean section: incidence, causes, associated factors and outcomes: a national prospective study from Jordan.* Gynecol Obstet Case Rep, 2017. **3**(3): p. 55.

4. WHO, *WHO statement on caesarean section rates.* Sexual and Reproductive Health and Research, 2015.

5. Betrán, A.P., et al., *WHO statement on caesarean section rates.* Bjog, 2016. **123**(5): p. 667.

6. Betrán, A.P., et al., *The increasing trend in caesarean section rates: global, regional and national estimates: 1990-2014.* PloS one, 2016. **11**(2): p. e0148343.

7. Rockville, M., USA: GSS, GHS, and ICF International, *Ghana demographic and health survey 2014.* Ghana Statistical Service GHS, ICF International (2015), 2014.

8. Adewuyi, E.O., et al., *Cesarean delivery in Nigeria: prevalence and associated factors—a population-based cross-sectional study.* BMJ open, 2019. **9**(6): p. e027273.

9. Long, Q., et al., *Caesarean section rates in Mozambique.* BMC pregnancy and childbirth, 2015. **15**(1): p. 1-9.

10. E, E., *demographic and health survey 2000: key indicators report. .* The DHS Program ICF:, 2000. **206.**

11. E, E., *demographic and health survey 2016: key indicators report.* The DHS Program ICF, 2016. **363: 364.**

12. Ye, J., et al., *Association between rates of caesarean section and maternal and neonatal mortality in the 21st century: a worldwide population-based ecological study with longitudinal data.* BJOG: An International Journal of Obstetrics & Gynaecology, 2016. **123**(5): p. 745-753.

13. Gjonej, R., et al., *The reasons of rising trend of cesarean section rate year after year. A retrospective study.* International Journal of Nursing and midwifery, 2015. **7**(1): p. 9-15.

14. Harrison, M.S. and R.L. Goldenberg, *Cesarean section in sub-Saharan Africa.* Maternal health, neonatology and perinatology, 2016. **2**(1): p. 1-10.

15. Ayalew, M., et al., *Magnitude of cesarean section delivery and its associated factors among mothers who gave birth at public hospitals in Northern Ethiopia: Institution-based cross-sectional study.* Journal of multidisciplinary healthcare, 2020: p. 1563-1571.

16. Gutema, H. and A. Shimye, *cesarean section and associated factors at mizan aman general hospital, southwest Ethiopia.* J Gynecol Obstet, 2014. **2**(3): p. 37-41.
17. Gebremedhin, S., *Trend and socio-demographic differentials of Caesarean section rate in Addis Ababa, Ethiopia: analysis based on Ethiopia demographic and health surveys data.* Reproductive health, 2014. **11**(1): p. 1-6.
18. Yisma, E., et al., *Cesarean section in Ethiopia: prevalence and sociodemographic characteristics.* The Journal of Maternal-Fetal & Neonatal Medicine, 2019. **32**(7): p. 1130-1135.
19. Sakae, T.M., P.F. Freitas, and E. d'Orsi, *Factors associated with cesarean section rates in a university hospital.* Revista de saude publica, 2009. **43**: p. 472-480.
20. Taye, M.G., et al., *Prevalence and factors associated with caesarean section in a comprehensive specialized hospital of Ethiopia: a cross-sectional study; 2020.* Annals of Medicine and Surgery, 2021. **67**: p. 102520.
21. Tsega, F., et al., *Prevalence of cesarean section in urban health facilities and associated factors in Eastern Ethiopia: hospital based cross sectional study.* J Preg Child Health, 2015. **2**(3): p. 169-73.
22. tw, b., *1. bank tw (Jul 15, 2021) The world bank Ethiopia.* 2021.
23. Tsai, P.-J., et al., *Spatial autocorrelation analysis of health care hotspots in Taiwan in 2006.* BMC Public Health, 2009. **9**: p. 1-13.
24. Kulldorff, M., *A spatial scan statistic.* Communications in Statistics-Theory and methods, 1997. **26**(6): p. 1481-1496.
25. Jung, I., M. Kulldorff, and A.C. Klassen, *A spatial scan statistic for ordinal data.* Statistics in medicine, 2007. **26**(7): p. 1594-1607.
26. Pezzulo, C., et al., *Geographical distribution of fertility rates in 70 low-income, lower-middle-income, and upper-middle-income countries, 2010–16: a subnational analysis of cross-sectional surveys.* The Lancet Global Health, 2021. **9**(6): p. e802-e812.
27. Pezzulo, C., et al., *The geography of fertility rates in low and middle-income countries: a subnational analysis of cross-sectional surveys from 70 countries, 2010-16.* The Lancet Global Health, 2021. **9**(6): p. E802-E812.

28. Gribov, A. and K. Krivoruchko, *Empirical Bayesian kriging implementation and usage.* Science of the Total Environment, 2020. **722**: p. 137290.

29. Austin, P.C. and J. Merlo, *Intermediate and advanced topics in multilevel logistic regression analysis.* Statistics in medicine, 2017. **36**(20): p. 3257-3277.

30. Kakujaha-Matundua, O., *Towards Identifying and estimating public expenditure on gender-based violence in Namibia.* SOCIAL SCIENCES DIVISION (SSD), 2015. **110**.

31. Statistics, U.B.O. and I. MACRO, *Uganda Demographic and Health Survey.* Kampala: Uganda Bureau of Statistics, 2011.

32. Acharya, K. and Y.R. Paudel, *Trend and sociodemographic correlates of cesarean section utilization in Nepal: evidence from demographic and health surveys 2006-2016.* BioMed research international, 2021. **2021**.

33. Al Rifai, R.H., *Trend of caesarean deliveries in Egypt and its associated factors: evidence from national surveys, 2005–2014.* BMC pregnancy and childbirth, 2017. **17**(1): p. 1-14.

34. Haider, M.R., et al., *Ever-increasing Caesarean section and its economic burden in Bangladesh.* PloS one, 2018. **13**(12): p. e0208623.

35. Zhang, J., et al., *The associated factors of cesarean section during COVID-19 pandemic: a cross-sectional study in nine cities of China.* Environmental health and preventive medicine, 2020. **25**(1): p. 1-7.

36. *Demographic EE (2011) Health Survey 2011. Central Statistical Agency. Adis Abab, Ethiopia.*

37. Fesseha, N., et al., *A national review of cesarean delivery in Ethiopia.* International Journal of Gynecology & Obstetrics, 2011. **115**(1): p. 106-111.

38. de Loenzien, M., et al., *Magnitude and correlates of caesarean section in urban and rural areas: A multivariate study in Vietnam.* PloS one, 2019. **14**(7): p. e0213129.

39. Rydahl, E., et al., *Cesarean section on a rise—Does advanced maternal age explain the increase? A population register-based study.* PloS one, 2019. **14**(1): p. e0210655.

40. Yaya, S., et al., *Disparities in caesarean section prevalence and determinants across sub-Saharan Africa countries.* Global health research and policy, 2018. **3**: p. 1-9.

41. Mekonnen, Z.A., et al., *Multilevel analysis of individual and community level factors associated with institutional delivery in Ethiopia.* BMC research notes, 2015. **8**(1): p. 1-9.

42. Beyene, M.G., et al., *Cesarean delivery rates, hospital readiness and quality of clinical management in Ethiopia: national results from two cross-sectional emergency obstetric and newborn care assessments.* BMC pregnancy and childbirth, 2021. **21**: p. 1-11.

43. Tsegaye, H., et al., *Prevalence and associated factors of caesarean section in Addis Ababa hospitals, Ethiopia.* Pan African Medical Journal, 2019. **34**(1).

44. Shibre, G., et al., *Magnitude and trends in socio-economic and geographic inequality in access to birth by cesarean section in Tanzania: evidence from five rounds of Tanzania demographic and health surveys (1996–2015).* Archives of Public Health, 2020. **78**: p. 1-10.

45. Logan, R.A., et al., *Health literacy: A necessary element for achieving health equity.* NAM perspectives, 2015.

 Click here to access/download;Figure;Fig 3.tif

Spatial Autocorrelation Report

Moran's Index:	1.009090
z-score:	4.966507
p-value:	0.000001

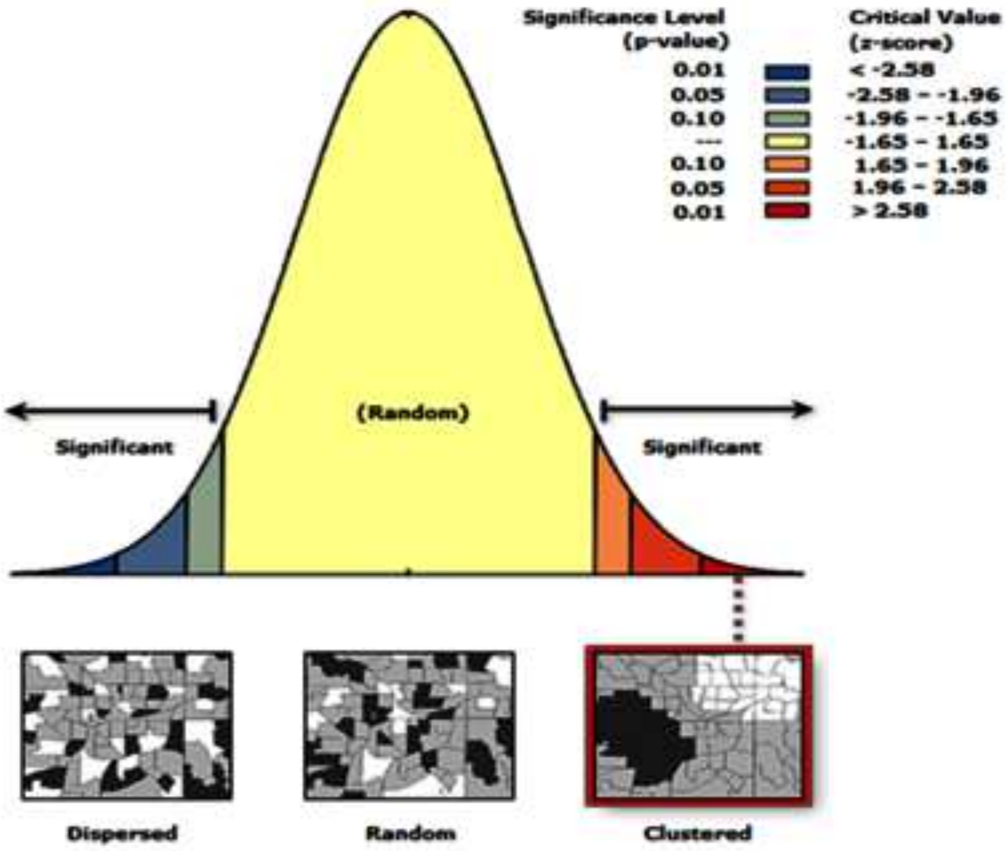

Given the z-score of 4.9665068324, there is a less than 1% likelihood that this clustered pattern could be the result of random chance.

Click here to access/download;Figure;Fig 1.tif

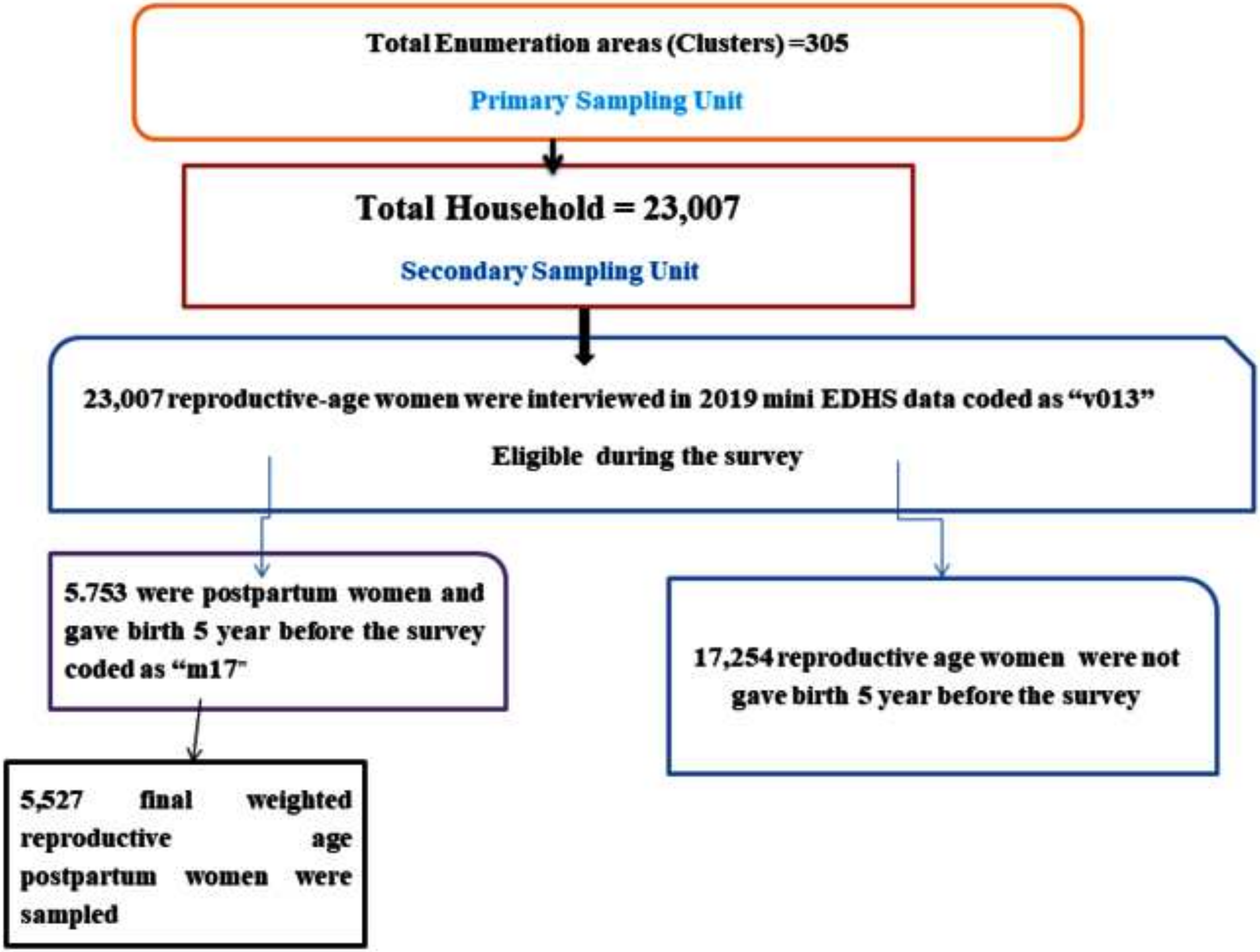

Figure

Click here to access/download;Figure;Fig 2.tif

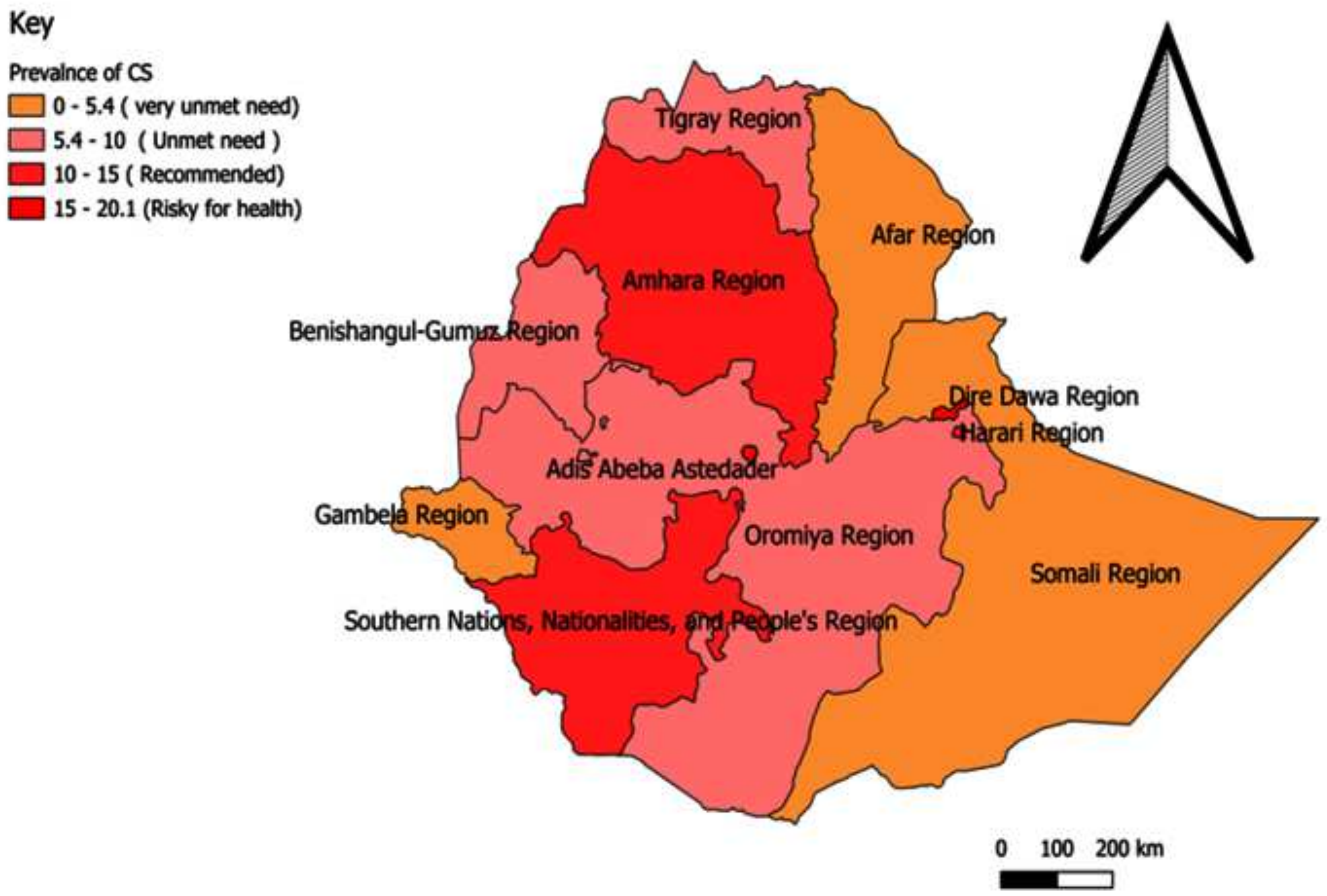

Figure

Click here to access/download;Figure;Fig 4.jpg

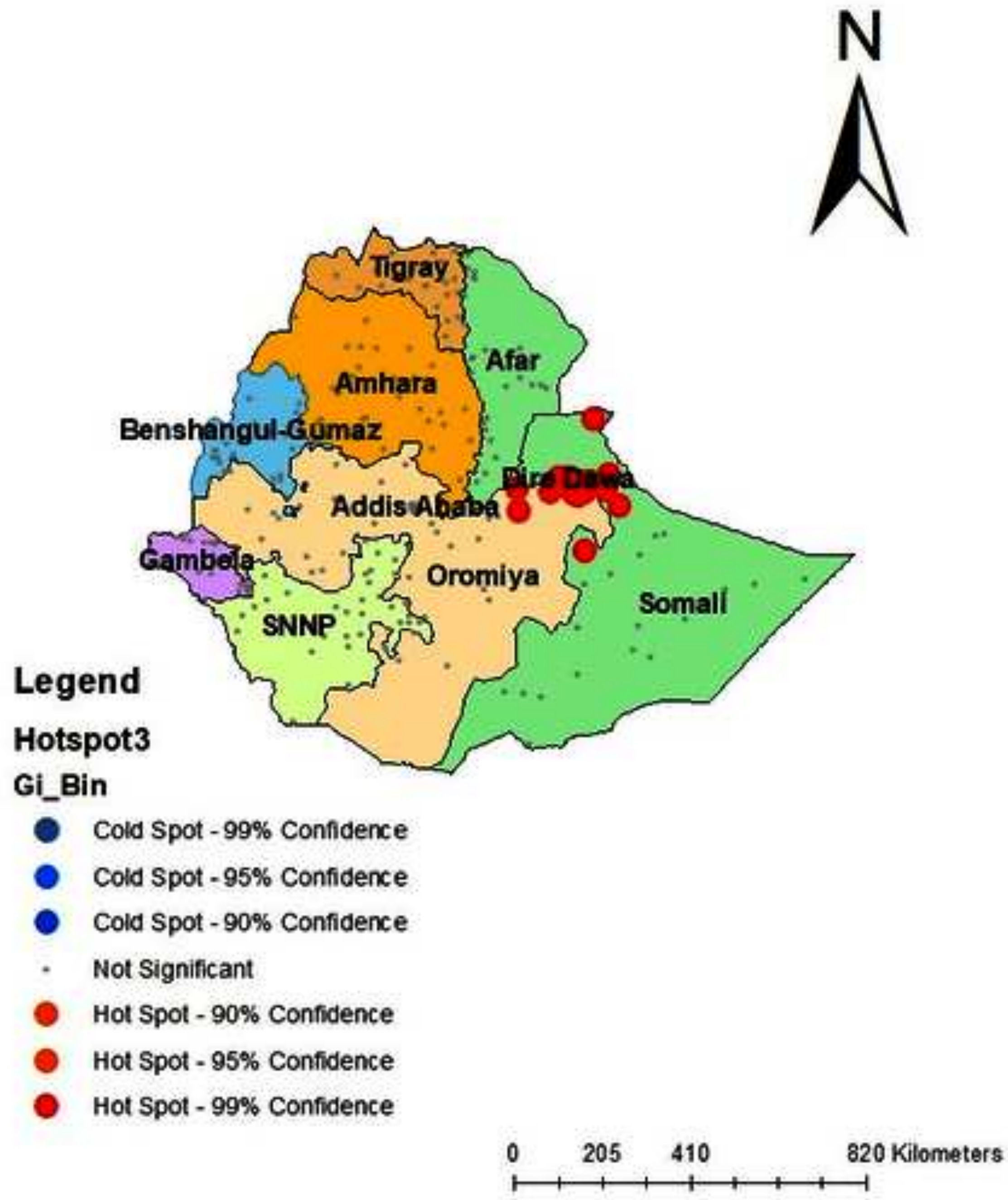

Figure

Click here to access/download;Figure;Fig 5.tif

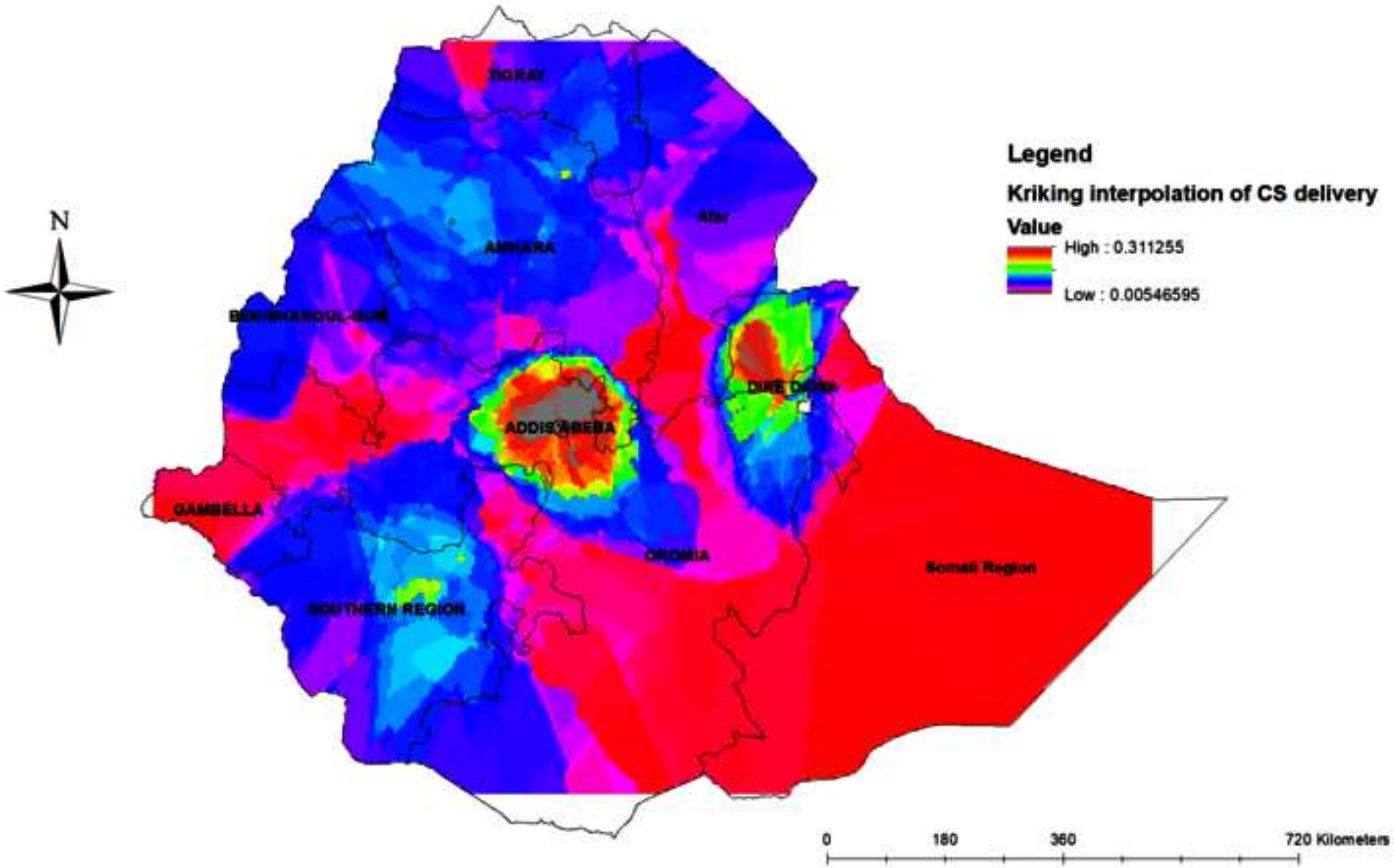

Click here to access/download
Other
PLOSOne_Human_Subjects_Research_Checklist.docx

Revised Manuscript with Track Changes

Geospatial Patterns~~, Prevalence~~, and Individual and Community Levels Factors of Cesarean Section Deliveries in Ethiopia: A Spatial and Multilevel Analysis

Abel Endawkie[1*] Shimels Derso Kebede[2], Natnael Kebede[3], Mengistu Mera Mihretu[4], Ermias Bekele[2], Kokeb Ayele[3], Lakew Asmare[1], Fekade Demeke Bayou[1], Mastewal Arefaynie[5], Yawkal Tsega[4]

Affiliation

[1]Department of Epidemiology and Biostatistics, School of Public Health, College of Medicine and Health Sciences, Wollo University, Dessie, Ethiopia.

2Department of Health Informatics, School of Public Health, College of Medicine and Health Sciences, Wollo University, Dessie, Ethiopia

3Department of Health Promotion, School of Public ~~health~~Health College of Medicine Health Sciences, Wollo University, Dessie, Ethiopia 4Department of Medical Nursing.

4Department of Health System and Management, School of Public Health, College of Medicine and Health Sciences, Wollo University, Dessie, Ethiopia

5Department of Reproductive and Family Health, School of Public Health, College of Medicine and Health Sciences, Wollo University, Dessie, Ethiopia

Corresponding author Abel Endawkie

Email Adress **abelendawkie@gmail.com**

Formatted: Header
Formatted: Space After: 0 pt, Line spacing: 1.5 lines
Formatted: Justified, Line spacing: 1.5 lines
Formatted: Superscript
Formatted: Line spacing: 1.5 lines
Formatted: Footer

Abstract

Background: Cesarean Section (CS) is the most popular ~~surgeries~~ surgery worldwide in obstetric care to save a mother's or the child's life. However spatial ~~distribution, prevalence,~~ patterns and multilevel factors of CS delivery in Ethiopia ~~using 2019 Mini EDHS is~~ are limited. Therefore, this study aimed to determine geospatial patterns~~, prevalence,~~ and individual and community~~ levels~~-level factors of ~~cesarean section~~ CS deliveries in Ethiopia.

Method: ~~The~~ A cross-sectional study using secondary data was ~~analyzed using 2019 mini EDHS with 5527~~conducted among 5,527 weighted ~~sample~~ samples of reproductive~~ age women ~~-age mothers using the 2019 Ethiopian mini demographic and health survey. Spatial and multilevel analyses were employed to determine spatial ~~distribution~~ hotspots and colds pots, and multilevel factors of CS delivery in Ethiopia. Significance was declared at p-value <0.05 and adjusted odds ratio (AOR) with 95% confidence interval (CI) was reported.

Result: The prevalence of CS delivery in Ethiopia was 5.4% with 95%CI (4.6~~%~~ %, 6.4%). ~~Age, 15~~ The spatial autocorrelation shows CS was clustered in Ethiopia (global Moran's index = 1.009, and p-value<0.001). Spatial hotspot indicates CS prevalent in Addis Ababa, Diredewa, Oromo, and Somali regions. The odds of CS delivery were higher among mothers aged 24~~ [~~-35: AOR~~=~~ = 1.98, 95% CI~~=(~~ (1.3, 3.1~~)]~~), and ~~[~~35-49: AOR=3.7, 95% CI~~=(~~(2.2, 6.1~~)],~~), live with female household heads: AOR =1.9, 95% CI (1.2, 3.1), mothers with primary AOR = 1.6, 95% CI (1.07, 2.7), secondary: AOR = 2.4, 95% CI (1.3, 4.25) and higher education: AOR = 2.06, 95% CI (1.03, 4.2), multiple births: AOR = 8.1, 95% CI (3.4, 19.74), mothers in Addis Ababa: AOR = 3.4, 95% CI (1.1, 11.9) and Diredewa: AOR = 7, 95% CI (1.23, 40.7) as compared to their counterparts.

Conclusion: In Ethiopia, CS deliveries remain below the recommended World Health Organization rates with distinct geospatial variation. Geographically, CS delivery is concentrated in urban areas like Addis Ababa and Diredewa, as well as in parts of the Somali and Oromia regions. Maternal age, female household head~~ [AOR=1.9, 95% CI=(1.2, 3.1)], muslim [AOR=0.4, 95% CI=(0.2, 0.7)], primary [AOR=1.6, 95% CI=(01.07, 2.7)], secondary[AOR=2.4, 95% CI=(1.3, 4.25)], higher education [AOR=2.06, 95% CI (1.03, 4.2)], multiple birth[AOR=8.1, 95%~~

Formatted: Header

Formatted: Font: 12 pt

Formatted: Font: 12 pt, Bold

Formatted: Font: 12 pt

Formatted: Footer

~~CI=(3.4, 19.74)], Addis Ababa [AOR=3.4 95% CI=(1.1, 11.9)], and Dire Dawa [AOR=7, 95%CI=(1.23, 40.7)] were associated with CS.~~

~~Conclusion~~

~~The findings suggest a slight increase in the prevalence of cesarean section (CS) deliveries in Ethiopia, although remaining below the World Health Organization's (WHO) recommended rates. Geospatial analysis highlights significant variations in CS prevalence, with hotspot areas observed in urban regions such as Diredewa, Harerie, and parts of Somali and Oromia. Factors including maternal age, sex of the household head~~, Muslim religion, higher educational status, and ~~types of birth~~multiple births at the individual level, and region at the community level~~.~~ were factors of CS delivery in Ethiopia. Therefore, ~~to align with WHO guidelines,~~ efforts should be ~~directed toward optimizing CS delivery rates and ensuring appropriate distribution across regions in Ethiopia.~~ geared towards strategies and interventions to ensure fair access to CS delivery in line with WHO recommendations, especially in the regions where the CS delivery rate is low in Tigray, Amhara, Afar, and Benishangul Gumze regions.

~~Keyword~~**Keywords**: Cesarean Section, Spatial Pattern, Multilevel, ~~2019 Mini-EDHS:~~Ethiopia

~~Introduction~~

~~Cesarean section (CS) is the most usually lifesaving surgical procedure in obstetric care. It is the birth of a fetus through a surgical incision made in the uterine and abdominal walls of a pregnant woman. Preventing maternal and newborn morbidity and mortality rates is the primary purpose for conducting it, either for fetal or maternal reasons(1). Delivery women use CS more frequently than ever before, and this trend is expected to continue in both rich and developing nations over the next ten years(2, 3), even though there is still a significant lack of safe CS in resource-constrained nations. Since 1985, World Health Organization (WHO) states that, the ideal rate for CS should be between 10-15%(4). The average global CS rate, according to recent statistics from~~

Formatted: Header

Formatted: Line spacing: 1.5 lines

Formatted: Font: 12 pt, Bold

Formatted: Font: 12 pt

Formatted: Footer

Formatted: Header

154 nations, is 21.1%; rates range from 8.2% in the least developed countries to 27.2% in the more developed ones Latin America and the Caribbean have the greatest rates (42.8 percent, 23 countries, and 91.2 percent birth coverage), whereas Sub-Saharan Africa has the lowest rates (5.0 percent, 39 nations, and 88.6 percent birth coverage(5). Less than 5% of people have (CS), which indicates an unmet need, according to the WHO recommendation. However, a prevalence of greater than 15% might put the mother and kid at risk(6) This conclusion is further supported by reports from the population-based study, which show that CS rates in the population greater than around 10% are not linked to lower rates of maternal and newborn mortality(7). The number of CS deliveries per 100 births, or the CS rate, has a negative and statistically significant linear connection with infant death, according to another ecological study (8). Despite all of these facts, the medically non-indicated CS section has seen a large increase in practice in recent years(9). According to data from the WHO survey, women's health and medical expenses may be significantly impacted by the global rise in medically unnecessary CS. These effects may include an increased risk of infection, a longer healing period, significant bleeding, and an increase in iatrogenic injuries, breathing difficulties, and even death(10, 11). The same result was also reached by a multi-country facility-based survey(2). Numerous research demonstrate that a rise in CS has been associated with both medically and non-medically indicated factors, such as the kind of health facility, socio-demographic traits, and maternal health of women(12, 13). Several factors include the mother's age, birth order, area of residence, socioeconomic situation, educational attainment, history of childhood sexual abuse, and obstetric problems, maternal request, and income level (7, 14-17). The prevalence of Cesarean section in Ethiopia is below the WHO's recommended threshold of 10% (6). Furthermore, there has been no notable increase in the country's population-based CS rates in the 2000 and 2016 Ethiopia Demographic and Health Surveys (EDHS), which

Formatted: Footer

Formatted: Header

were found to be very low (0.7 percent and 1.9 percent), respectively(18, 19). This figure is lower than that of several African countries, including Ghana 13% (20) , Nigeria 2.1% (21) , and Mozambique, 4.7 (22) . The relatively low prevalence of CS in the Ethiopian population reflects unmet demands, which may contribute to the country's poor maternal and newborn outcomes(23).Different studies reported different prevalence and associated factors for caesarean section delivery in Ethiopia. Evidence from hospital-based births in Ethiopia indicates that first-parity births, births for which women had more than three Antenatal Care (ANC) visits, urban births, higher education levels, and higher economic statuses are the groups of women most likely to undergo cesarean delivery by caesarean section(3, 5, 14, 15). These studies frequently used small sample sizes and were prone to selection bias(10, 15, 16) and a small sample size and a lack of use of more advanced statistical analysis, like multilevel analysis and spatial analysis were limitations of a few studies that were employed using population-based data. Knowing this evidence spatial distribution, prevalence, and individual and community level factors of caesarean section delivery in Ethiopia using national representative data of 2019 Mini Ethiopian Demographic and Health Survey (MEDHS) is limited, even though it has public health important as evidence of obstetric care .Therefore, this study aimed to determine geospatial patterns, prevalence, and individual and community levels factors of cesarean section deliveries in Ethiopia using spatial and multilevel analysis of 2019 MEDHS national representative data.

Introduction

Cesarean section (CS) is the most usually lifesaving surgical procedure in obstetric care. It is the birth of a fetus through a surgical incision made in the uterine and abdominal walls of a pregnant woman. Preventing maternal and newborn morbidity and mortality rates is the primary purpose of conducting it, either for fetal or maternal reasons [1]. CS more frequently than ever before, and

Formatted: Footer

this trend is expected to continue in the next ten years [2, 3], even though there is still a significant lack of safe CS in resource-constrained nations.

Since 1985, the World Health Organization (WHO) recommends that CS prevalence should be between 10-15% of all births among reproductive age mothers in the country or specific community [4]. According to the WHO statement, CS prevalence less than 5% among reproductive age mothers in the country or specific community indicates unmet need, whereas a prevalence of CS greater than 15% among reproductive age mothers might put the mother and new birth at risk [5].

The average global CS prevalence according to recent statistics from 154 nations is 21.1% and it ranges from 8.2% in the least developed to 27.2% in the more developed countries [6]. The prevalence of CS delivery in African countries Ghana (13%) [7], Nigeria (2.1%) [8], and Mozambique (4.7%) [9]. The prevalence of CS delivery in the 2000 and 2016 Ethiopia Demographic and Health Surveys (EDHS) were 0.7 percent and 1.9 percent respectively [10, 11]. A population-based study, showed that CS rates greater than 10% are not linked to lower the rates of maternal and newborn mortality [12]. The number of CS deliveries per 100 births, or the CS rate, has a significant linear connection with infant death, according to another ecological study [13]. Despite all of these facts, the medically non-indicated CS section has seen a large increase in practice in recent years [14]. A research conducted in Ethiopia showed that a rise in CS has been associated with both medically and non-medically indicated factors, such as the kind of health facility, socio-demographic traits, and maternal health of mothers [15, 16]. Study conducted in Ethiopia indicates that CS delivery increased risk of infection, a longer healing period, significant bleeding, and an increase in iatrogenic injuries, breathing difficulties, and even death [17, 18]. Different studies reported different prevalence and associated factors for cesarean section delivery in Ethiopia [3, 6, 19, 20].

Mother's age, birth order, first-parity births, Antenatal Care (ANC) visits, urban residence, higher education levels, and higher economic statuses were factors of CS delivery among mothers in Ethiopia [3, 6, 19-21]. These studies used small sample sizes which was not representative and were prone to selection bias [3, 6, 19, 20]. The 2019 mini EDHS is sufficient to provide nationwide data to conduct analysis of individual and community level factors of CS delivery in Ethiopia Moreover, in Ethiopia spatial pattern of caesarean section delivery using national representative data of 2019 mini Ethiopian Demographic and Health Survey mini (EDHS) is limited. Therefore,

Formatted: Header

Formatted: Footer

this study aimed to determine geospatial patterns, and individual and community levels factors of cesarean section deliveries in Ethiopia using nationwide 2019 mini EDHS data.

Method

Study ~~setting and Period~~Setting

The study was done in Ethiopia which ~~is located in east Africa which have~~has a diverse population. It is bordered on the west by Sudan, on the east by Somalia and Djibouti, on the north by Eritrea, and on the south by Kenya. The country has a total area of 1,112,000 square kilometers Ethiopia is divided into eleven regions and two municipal governments. Tigray, Afar, Amhara, Oromiya, Somali, Benishangul-Gumuz, Southern Nations Nationalities and People (SNNP), Gambela, Sidama, South Western, and Harari are among the regions involved. Addis Ababa and Dire Dawa are administrative cities. Data were accessed from their URL: ~~www.dhsprogram.com~~www.dhsprogram.com by contacting them through personal accounts after justifying the reason for requesting the data~~(24).~~ [22].

Study ~~design~~Design and Source ~~population~~Population

The research was conducted using secondary data ~~of~~from a cross-sectional study used in ~~**2019MEDHS. This data analyzed from women's fertile and**~~2019 mini EDHS using children's records data. The source population was reproductive age mothers (15-49) ~~**using surveys conducted on**~~who gave birth 5 years before the ~~**recent DHS/2019 MEDHS data of Ethiopia using children record**~~survey.

Sample Size and Sampling Methods

A total of ~~5526~~5,526.93 ~ ~~5527~~5,527 weighted reproductive ~~women~~mothers were used in this analysis as a sample that was aimed to represent all of the country's regions and administrative cities from ~~5753 women~~5,753 mothers used in ~~MEDHS~~mini EDHS 2019. ~~MEDHS 2019~~The sample was stratified and selected in two stages. In the first stage, stratification was conducted by region, and then each region was stratified as urban and rural. The sample size was then allocated using a probability-proportional allocation method. There were 305 enumeration areas (EAs) ~~were~~ chosen for the ~~2019MEDHS.~~ 2019 mini EDHS (Fig1).

Figure 1. Sampling and exclusion procedures to identify the final sample size of the study

Formatted: Header
Formatted: Line spacing: 1.5 lines
Formatted: Font: +Headings (Cambria), 13 pt
Formatted: Heading 2, Left, Line spacing: 1.5 lines
Formatted: Font: +Headings (Cambria), 13 pt
Formatted: Font: +Headings (Cambria), 13 pt, Not Bold
Formatted: Font: Not Bold, Font color: Auto
Formatted: Font: Not Bold, Font color: Auto
Formatted: Font: Not Bold, Font color: Auto
Formatted: Font: Not Bold, Font color: Auto
Formatted: Font: Not Bold, Font color: Auto
Formatted: Font: Not Bold, Font color: Auto
Formatted: Font: 13 pt
Formatted: Font: +Headings (Cambria), 13 pt
Formatted: Font: +Headings (Cambria), 13 pt, Not Bold
Formatted: Space After: 0 pt, Line spacing: 1.5 lines
Formatted: Footer

Variable ~~measurement~~Measurement

~~Dependent variables was cesarean~~Cesarean section delivery categorized as Yes~~/No~~"1" and No "0" was the dependent variable and the individual-level factors ~~including (~~included: age, sex of household, religion, wealth index, marital status, educational status, ANC visit, ~~Types~~types of birth, place of delivery~~,)~~, and community-level factors~~ (~~: region, and residence~~)~~ were considered as the independent variable.

Data ~~processing~~Processing and ~~analysis~~Analysis

Data ~~cleaning was conducted to check for consistency with the MEDHS 2019 descriptive reports. Recoding, variable generation, labeling, and analysis were done by using Stata version 17.0. Descriptive statistics were done to describe the study participants in relation to socio-demographic characteristics which were presented in tables and text. Sample weight was used~~used were extracted, cleaned, coded, and analyzed using STATA version 17 statistical software. Sample weights were done before further analysis to compensate for the unequal probability of selection between the strata that were geographically defined and for non-responses. Descriptive statistics were calculated and presented using tables, figures, and narratives.

Spatial Analysis:

To execute the spatial analysis~~: After~~, the weighted proportions of outcome variables (CS delivery) with cluster number were computed in STATA version 17 and the spatial analysis was computed using QGIS version 3.28.15 and ArcGIS version 10.7.1 statistical software.

Spatial Distribution: The descriptive spatial analysis was conducted to determine distribution of CS delivery in Ethiopia using QGIS version 3.28.15.

Spatial Autocorrelation Analysis

The spatial autocorrelation ~~was assessed and significant at P-value 0.0001 and~~ (Global Moran's I) statistic measure is used to assess whether CS delivery is dispersed, clustered, or randomly distributed in Ethiopia. Moran's I value close to − 1 shows dispersed CS delivery, close to + 1 shows clustered, and if Moran's I value zero shows randomly distributed [23]. In this study global spatial autocorrelation indicate CS delivery is clustered at p-value 0.0000 and Global Moran's I (MI) =1.0009~~, we had conducted mapping.~~

Hotspot Analysis: The spatial hotspot analysis was conducted using Getis-Ord Gi* hot spot analysis of ArcGIS version 10.7.1 to examine where spatial clusters of CS delivery occur on the

Formatted: Header

Formatted: Font: +Headings (Cambria), 13 pt

Formatted: Heading 2, Left, Line spacing: 1.5 lines

Formatted: Font: +Headings (Cambria), 13 pt

Formatted: Font: +Headings (Cambria), 13 pt, Not Bold

Formatted: Space After: 0 pt, Line spacing: 1.5 lines

Formatted: Font: +Headings (Cambria), 13 pt

Formatted: Heading 2, Left, Line spacing: 1.5 lines

Formatted: Font: +Headings (Cambria), 13 pt

Formatted: Font: +Headings (Cambria), 11 pt

Formatted: Font: +Headings (Cambria), 11 pt, Not Bold

Formatted: Space After: 0 pt, Line spacing: 1.5 lines

Formatted: Font: Not Bold, Font color: Custom Color(RGB(13,13,13))

Formatted: Font color: Auto

Formatted: Font color: Auto

Formatted: Space After: 0 pt, Line spacing: 1.5 lines

Formatted: Font: 12 pt

Formatted: Font: 12 pt

Formatted: Font: 11 pt

Formatted: Footer

Formatted: Header

landscape by identifying areas with statistically significant clustering of relative high values (hot spots) or relative low values (cold spots). This measure indicates the presence or absence of significant spatial clustering, with the null hypothesis being that there is no difference in characteristiccharacteristics between a unit and its spatial neighbors (25-28)[24-27].

Formatted: Font: 12 pt, Not Bold, Font color: Custom Color(RGB(13,13,13))

Mixed effect model**Spatial Interpolation:** Spatial interpolation was used to estimate the proportion of CS delivery distribution in un-sampled areas, based on observed measurements in the sampled enumeration areas. Although there are different deterministic and geostatistical interpolation methods available, we opted to use the Ordinary Kriging spatial interpolation method since it incorporates spatial autocorrelation statistically optimizes the weight, and has low residual and mean square error [28].

Multilevel Analysis: Multilevel analysis was conducted after checking the eligibility of the data tofor multilevel analysis by using the intra-cluster correction coefficient (ICC). Since ICC is greater than 10% (ICC=44.1%) calculated as below [29].

$$ICC = \frac{\delta 2}{\delta 2 + \pi 2/3} \text{, where} \text{ -- } (1)$$

Where δ2 indicates the estimated variance of clusters, multi-level regression was conducted to assess factors related to individual and community-level characteristics of cesarean section. Multilevel analysis was considered appropriate due to the hierarchical nature of the DHS data as well as the estimation of individual and community--level effects. The log of the probability of a cesarean section was modeled using a two--level multilevel model as follows: $Log[\frac{\pi ij}{1-\pi ij}] = \beta 0+\beta 1Xij + B2\ Zij+\mu j+ eij$, Where i and j are individual level and community level(29) unites respectively; X and Z refers to individual and community level variables respectively; πij is the probability of cesarean section for the ith women[30] units respectively; X and Z refer to individual and community level variables respectively; πij is the probability of cesarean section for the ith mothers in the jth community; β's indicates the fixed coefficients. (B0) is the intercept, the effect on the probability of cesarean section in the absence of influencing factors; and μj showed the random effect (the effect of the community on the cesarean section of the jth community) and eij showed random errors at the individual level. By assuming each community had a different intercept (B0) and fixed coefficient (β), the clustered data nature and intra and inter-community variations were taken into account. Data organized in tointo, analyses of cesarean

Formatted: Footer

section yes/no which has the binary response and ~~run~~runs mixed effect multi-level logistic regression model by considering two levels (individual and community levels). During analysis first, bi-variable multilevel logistic regression was fitted, and variables with p value less than 0.2 at model I and model II were selected to develop the 3rd model (the final model). The analysis was done in four models. The first model was, model-0 (empty model or null model), the second model was, model-I (analyzing only individual~~-~~-level ~~variable~~variables), the 3rd model was, model-II (analyzing only community-level ~~variable~~variables), the last model, model-III (analyzing both community~~-~~-level and individual~~-~~-level variables based on the cut of the point). Variables with p value less than 0.05 at model-III were significantly associated with cesarean section. The random~~-~~effects (variations) were measured by using ICC (model-0), Median Odds Ratio (MOR) in (model-I and II), and Proportional Change in Variance (PCV) was measured to show variation between clusters. ICC shows the variation in cesarean section among ~~women~~mothers due to community characteristics. MOR is the median value of the odds ratio between the area at the highest risk and the area at the lowest risk when randomly picking out two areas and it was calculated as follows

MOR=exp~~.($\sqrt{2 + \delta 2 + 0.6745}$)≈ exp(2.23). In this study, MOR shows the extent to which the individual probability of cesarean section delivery determined by place of residence.~~ ($\sqrt{2 + \delta 2 + 0.6745}$. PCV measures the total variation attributed ~~by~~to individual-level variables and area-level variables in the final model (model-III). It is calculated as below.

PCV~~=[~~= [δ2 of null model−δ2 of each model)/δ2 of null model~~].~~], where δ2 of the null model is used as a reference. Multicollinearity was checked among explanatory variables by using standard error at a cut of point±2 and there is no Multicollinearity since~~,~~ the standard errors were between±2. The appropriateness of the mixed model (model fitness) is checked using Inter-Class Correlation and model selection was based on Log Likelihood Ratio (LLR) or ~~AIC/ BIC~~Akakian information criteria ~~(**Table 1**).~~ AIC and Bayesian information criteria (BIC).

~~Table 1 Model fitness and selection~~

~~Model~~	~~ICC~~	~~Variance~~	~~PCV~~	~~MOR~~	~~LLR~~	~~AIC~~	~~BIC~~
~~Model 0~~	~~44.1%~~	~~2.3~~	~~Reference~~	~~exp(2.23)~~	~~-1202~~	~~2408~~	~~2421~~
~~Model 1~~		~~1.67~~	~~27.39%~~	~~exp(2.08)~~	~~-639~~	~~1423~~	~~1514~~

Formatted: Header

Formatted: Line spacing: 1.5 lines

Formatted: Line spacing: 1.5 lines

Formatted Table

Formatted: Footer

~~Model 2~~	~~32.07%~~	~~1.7~~	~~26.09%~~	~~exp(2.09).~~	~~-983~~	~~1993~~	~~2079~~
~~Model 3~~	~~31.2%~~	~~1.63~~	~~29.1%~~	~~exp(2.07).~~	~~-632~~	~~1320~~	~~1479~~

Ethical Approval: ~~The Demographic and Health Survey~~ No ethical approval was needed because we had used the demographic and health survey, which de-~~-~~identifies all data before making it public, and the used mini DHS data sets are openly accessible. ~~There are no variables at the level of human subjects in the WorldPop geographic data. Then an~~An authorization letter was ~~requesting~~requested to download ~~EDHS~~the DHS data set and this was ~~also~~ obtained from the Central Statistical Agency (CSA) after ~~requesting~~being requested at https://dhsprogram.com/. ~~And approvals of~~ The dataset and all methods ~~and data of Ethiopian demographic and health survey~~of this study were ~~performed~~ conducted according to the guidelines laid down in the Declaration of Helsinki principle and based on ~~Helsinki principles and Ethiopian~~DHS research guidelines.

Result

Socio-demographic characters ~~Respondents~~

~~From a total~~ of ~~5,753~~ reproductive age ~~[15–49] women, 5526.93~~mothers who gave birth 5 years before the survey

A total of, 5,527 weighted ~~sample~~ reproductive-age mothers [15–49] were included in the analysis. Among ~~this, 51(~~205 reproductive-age mothers, 48~~%)~~% of ~~women~~mothers in the age group of 15-24 have ~~deliver~~delivered via cesarean section, ~~(~~from 2, 961 reproductive-age mothers, 2.9~~%)~~% of ~~women~~mothers who ~~-~~cannot read and write have ~~deliver~~delivered via cesarean section. Among ~~study participants (~~4160 reproductive-age mothers, 11.1~~%)~~% of ~~women~~mothers residing in rural areas had ~~deliver~~delivered via cesarean section ~~(Table 2). —~~(Table 1).

~~Maternal health service utilization~~Table 1: Socio-demographic characters of ~~respondents~~

~~In regarding to utilization of maternal health service among study participants ,234 (8.1%) of women who had ANC visit deliver via cesarean section, in regarding to place of delivery 270(10.8) of women who deliver in private hospital deliver via cesarean section(**Table 3**).~~

Formatted: Header

Formatted: Space Before: 0 pt, After: 10 pt, Line spacing: 1.5 lines

Formatted: Font color: Auto

Formatted: Font: +Body (Calibri), Font color: Auto

Formatted: Font color: Auto

Formatted: Font color: Auto

Formatted: Font: Not Bold

Formatted: Font: Not Bold

Formatted: Default Paragraph Font, Font: +Body (Calibri), 11 pt, Font color: Custom Color(RGB(5,99,193))

Formatted: Font color: Auto

Formatted: Font color: Auto

Formatted: Font: 16 pt

Formatted: Space Before: 0 pt, After: 12 pt, Line spacing: 1.5 lines

Formatted: Font: 12 pt

Formatted: Font: 18 pt

Formatted: Space After: 0 pt, Line spacing: 1.5 lines

Formatted: Font: 12 pt

Formatted: Footer

Table 3. The maternal health service utilization of respondentsreproductive age mothers who gave birth by cesarean section5 years before the survey in Ethiopia Using, 2019 MEDHSmini EDHS (weighted).

Variable ___	Category	-CSCesarean section delivery			
		No	%	Yes	%
Age category	15-24	124	52	51	48
	25-34	2512	93.8	165	6.2
	35-49	1199	6.6	85	93.4
Sex of household	Male	4538	95	237	5
	Female	688	91.6	63	8.4
Place of residence	Urban	1229	89.9	138	11.1
	Rural	3998	96.1	162	3.9
Religion	Christian	3106	93.1	232	6.9
	Muslim	2120	96.8	69	3.2
Region	Tigray	346	93.3	25	6.7
	Afar	84	96.5	3	3.5
	Amhara	972	92.57	78	7.43
	Oromia	2120	95.9	90	4.1
	Somali	404	99.1	4	0.98
	Benishngul Gumz	63	94.03	4	5.97
	SNNP	1054	95.38	51	4.62
	Gambella	24	96	1	4
	Hareri	15	88.24	2	11.76
	Addis Abeba	118	75.64	38	24.36
	Diredewa	25	83.3	5	16.7
Marital status	Union	2951	91.5	274	8.5
	Not union	276	91.1	27	8.9
Educational status	Cannot read and write	2875	97.1	86	2.9
	Primary education	1875	93.75	125	6.25
	secondary education	1832	97.23	52	2.77
	Higher education	362	90.5	38	9.5
Wealth index	Poorest	1300	98.4	21	1.6

Formatted: Header

Formatted: Normal, Line spacing: 1.5 lines

Formatted: Line spacing: 1.5 lines, Tab stops: 0.8", Left

Formatted Table

Formatted: Line spacing: 1.5 lines

Formatted: Footer

	Poorer	1154	96.33	44	3.67
	Middle	997	95.5	47	4.5
	Richer	915	95.3	45	4.7
	Richest	861	85.8	144	14.3

Maternal health service utilization of reproductive age mothers who gave birth 5 years before the survey

Regarding utilization of maternal health services utilization, 234 (8.1%) of mothers who had ANC visits delivered via cesarean section, and 270 (10.8) of mothers who delivered in private hospitals delivered via cesarean section (Table 2).

Table **2**: The maternal health service utilization of reproductive age mothers who gave birth 5 years before the survey in Ethiopia Using 2019 mini EDHS (weighted).

Variable	Category	cesarean section delivery			
		No	%	Yes	%
Last delivery by previous CS	Yes	4653	99.6	20	0.4
	No	36	13.3	234	86.7
ANC Visit	No	993	98.9	11	1.1
	Yes	2689	91.99	234	8.01
Types of birth	Single	5109	94.96	271	5.04
	Multiple	118	80.3	29	19.7
Sex of New birthBirth	Male	2692	94.7	150	5.3
	Female	2535	94.4	150	5.6
Place of delivery	Public health facility	2235	89.2	270	10.8
	Private hospital	149	83.2	30	16.8

Spatial distribution, cluster (hotspot/cold spot) and interpolation of cesarean sectionCS delivery in Ethiopia

In the 2019 of MEDHS, The descriptive spatial analysis showed the areas identified as high clusters(risk for health) of cesarean sectionthat the prevalence of CS delivery include ,in Ethiopia was highest in Addis Abeba,and diredewa and Diredewa, and the low cluster (unmet need) werein Afar, Tigray, Oromia Benshangul gumz, region (Fig1).and Benishangul Gumz regions (Fig 2).

Figure 2: Spatial distribution of CS delivery in Ethiopia

Formatted: Header

Formatted: Line spacing: 1.5 lines

Formatted: Line spacing: 1.5 lines

Formatted: Line spacing: 1.5 lines

Formatted: Line spacing: 1.5 lines

Formatted: Line spacing: 1.5 lines

Formatted: Line spacing: 1.5 lines

Formatted: Font: 11 pt

Formatted: Footer

Spatial autocorrelation showed that there is a clustering of CS delivery [MI= 1.009, ~~Zscore~~Z score =4.99, and ~~P~~p-value <0.001] (~~Fig2).~~Fig 3).

Figure 3: Spatial autocorrelation analysis of CS delivery in Ethiopia

The Hotspot analysis showed that ~~hotspot[~~ CS delivery was hotspots [high prevalence] in Diredewa~~-~~, Harerie, Somali, and Oromia ~~region]~~regions, and cold ~~spot~~spots [low prevalence~~-~~] of ~~mixed milk feeding~~CS delivery in Tigray, ~~Amahara~~Amhara, Afar, and ~~Benshangul gumz,~~Benishangul Gumz region~~](Fig3).~~ (Fig 4).

Figure 4: Spatial hotspot analysis of CS delivery in Ethiopia

The spatial interpolation of CS delivery for locations where data were not obtained has been estimated using the standard Kriging technique. The highest estimated prevalence of CS delivery (red shaded) was observed in Addis Ababa, Diredewa and parts of Somalia regions (Fig 5).

Figure 5: Spatial interpolation analysis of CS delivery in Ethiopia

Prevalence of Cesarean Section Delivery in Ethiopia

The prevalence of cesarean section delivery was 5.4% (95% CI (4.6%-6.4%)) in Ethiopia ~~analyzed using 2019 EMDHS~~ .

Individual and Community~~-~~-Level Factors ~~Associated with~~ of Cesarean Section Delivery in Ethiopia

In the final model (model-III) maternal age, sex of the household, ~~-~~religion, educational status, types of birth from individual~~-~~-level ~~variable~~ variables, and~~,~~ the region from community-level ~~variable had a statistical association~~variables were associated with cesarean section delivery~~.~~ (Table 3). The ~~probability~~ odds of ~~having cesarean section~~CS delivery ~~was~~were 1.98 ~~and3~~and 3.7 times more among ~~participants who~~mothers whose ages are in the age group 25-34 and 35-49 as compared to ~~women~~mothers in the age group15-24 [AOR=1.98, 95% CI=(1.3, 3.1)] and [AOR=3.7, 95% CI=(2.2, 6.1)] respectively. The odds of ~~cesarean section~~CS delivery ~~was~~were 1.9 times ~~more~~higher among participants who ~~are~~lived in female ~~household~~head households as compared to male ~~household~~head households [AOR=1.9, 95% CI=(1.2, 3.1)]. The odds of ~~cesarean section~~CS delivery ~~was~~were 60% less likely among participants who are Muslim religion followers as compared to Christian religion followers [AOR=0.4, 95% CI~~=(~~= (0.2, 0.7)]. ~~Women~~Mothers who ~~were attending~~attended primary education, secondary education, and higher education were 1.6, 2.4, and 2.06 times more ~~-~~likely to have ~~cesarean section~~CS delivery ~~when~~as compared with those not ~~attend~~attended school [AOR=1.6, 95% CI=(01.07, 2.7)], [AOR=2.4, 95%

Formatted: Header
Formatted: Space After: 0 pt, Line spacing: 1.5 lines
Formatted: Space After: 0 pt, Line spacing: 1.5 lines
Formatted: Line spacing: 1.5 lines
Formatted: Space After: 0 pt, Line spacing: 1.5 lines
Formatted: Footer

CI=(1.3, 4.25)], and [AOR=2.06, 95% CI (1.03, 4.2)] respectively. The odds of cesarean sectionCS delivery waswere 8.1 times morehigher for multiple birth births as compared with never single birthbirths [AOR=8.1, 95% CI=(3.4, 19.74)]. WomenMothers who were living in Addis Ababa and Dire DawaDewa were 3.6 and7and 7 times more likely -to have cesarean section delivery when compareas compared to womenmothers who live in the Tigray region [AOR=3.4 95% CI=(1.1, 11.9)], and [AOR=7, 95%CI=(1.23, 40.7)] respectively(Table 4). (Table 3).

Random effects (measures of variation)

Table 3: The multilevel analysis of associated factors of cesarean section delivery among reproductive age mothers who gave birth 5 years before the survey in Ethiopia, 2019 mini EDHS (weighted).

Variable	Category	CS delivery		Bivariable		Model 0	Model 1	Model 2	Model 3
		No	Yes	P-value	95%COR	ICC=4 4.1%	95%AOR	95%AOR	95%AOR
Age category	15-24	124	51		Reference		Reference		Reference
	25-34	2512	165	0.022	1.4(1.05-2.00)		1.9(1.3-3.07)		1.98(1.3-3.1)*
	35-49	1199	85	0.003	1.73(1.21-2.47)		3.6(2.2-6.0)		3.7(2.2-6.1)*
Sex of household	Male	4538	237		Reference		Reference		Referenc[illegible]
	Female	688	63	0.00	1.73(1.5-2.3)		1.8(1.1-2.9)		1.9(1.2-3[illegible]
Place of residence	Urban	1229	138		Reference			Reference	Referenc[illegible]
	Rural	3998	162	0.000	0.36(0.3-0.5)			0.3(0.14-0.5)	0.6(0.3-1[illegible]
Religion	Christian	3106	232		Reference				Referenc[illegible]
	Muslim	2120	69	0.000	0.4(0.3-0.57)		0.4(0.23-0.69)		0.4(0.2-0[illegible]
Region	Tigray	346	25		Reference		Reference	Reference	Referenc[illegible]
	Afar	84	3	0.153	0.36(0.1-1.5)			0.4(0.07-2.3)	2.4(0.3-2[illegible]
	Amhara	972	78	0.74	1.07(0.8-1.7)			1.4(0.5-3.7)	2.4(0.8-6[illegible]
	Oromia	2120	90	0.016	0.6(0.36-0.9)			0.5(0.2-1.4)	1.8(0.6-5.2)
	Somali	404	4	0.00	0.14(0.1-0.4)			0.13(0.03-0.6	0.6(0.05-4.3)
	Benshngul	63	4	0.838	0.9(0.3-2.5)			0.9(0.2-4.2)	2.0(0.4-10.1)
	SNNP	1054	51	0.087	0.7(0.4-1.0)			1.0(0.4-2.7)	1.9(0.7-5.5)

Formatted: Header

Formatted: Font: 11 pt
Formatted: Line spacing: 1.5 lines
Formatted Table
Formatted: Font: 11 pt
Formatted: Line spacing: 1.5 lines
Formatted Table
Formatted: Font: 11 pt
Formatted: Line spacing: 1.5 lines
Formatted: Font: 11 pt, Not Bold
Formatted: Font: 11 pt
Formatted: Line spacing: 1.5 lines

Formatted: Footer

		Gambella	24	1	0.556	0.5(0.05-4.3)			0.4(0.03-5)	0.3(0.02-6.6)
		Hareri	15	2	0.83	1.2(0.2-7.36)			1(0.1-9.6)	2.9(0.2-38.4)
		Addis Abeba	118	38	0.000	4.3(2.4-7.36)			2.9(0.9-8.9)	3.6(1.1-11.9)*
		Dredewa	25	5	0.093	2.4(0.9-7.2)			2.5(0.6-11.02	7.0(1.2-40.7)*
Marital status		Union	2951	274		Reference		Reference		Referenc[illegible]
		Not union	276	27	0.01	1.7(1.1-2.6)		1.37(0.72-2.6)		1.3(0.7-2[illegible]
Educational status		Cannot read and write	2875	86		Reference		Reference		Referenc[illegible]
		Primary education	1875	125	0.000	2.27(1.7-3.0)		1.68(1.07-2.6)		1.6(1.07-2.7)*
		secondary education	1832	52	0.000	4.8(3.3-6.9)		2.3(1.3-4.12)		2.4(1.3-4.25)*
		Higher education	362	38	0.000	7.9(5.2-12.6		2.2(1.07-4.4)		2.06(1.1-4.2)*
Wealth index		Poorest	1300	21		Reference		Reference		Referenc[illegible]
		Poorer	1154	44	0.002	2.33(1.4-3.9)		1.4(0.68-3.05)		1.3(0.6-2[illegible]
		Middle	997	47	0.000	2.89(1.7-4.8)		0.79(0.4-1.7)		0.7(0.3-1.6)
		Richer	915	45	0.000	3.05(1.8-5.2)		0.9(0.4-1.9)		0.8(0.4-1.7)
		Richest	861	144	0.000	10.4(6.5-16.5)		1.3(0.5-2.6)		0.8(0.3-1.9)
Last birth by CS		Yes	43	25	0.000	1528.6(870-2684)				
		No	4842	281		Reference				
ANC Visit		No	11	1033		Reference		Reference		Referenc[illegible]
		Yes	272	2663	0.000	8.17(4.4-14.9)		1.94(0.8-4.3)		1.9(0.8-4.2)
Types of birth		Single	325	5261		Reference		Reference		Referenc[illegible]
		Multiple	24	143	0.000	4.6(3.1-7.1)		8.01(3.4-19.6)		8.1(3.4-19.7)*
Sex of New Birth		Male	178	2791		Reference				Reference
		Female	171	2613	0.6	1.06(0.84-1.34)				

Place of delivery	Public health facility	283	2280		Reference		Reference		Reference
	Private hospital	66	243	0.013	1.68(1.2-2.5)		1.4(0.8-2.5)		1.3(0.6-2.4)

Key *= significant at 95% confidence interval, AOR= Adjusted odds ratio, COR= crude odds ratio, ICC= Intra-Class Correlation, CS= Cesarean Section, ANC= Antenatal care, SNNP= South Nation Nationalities and Peoples

Random effects (measures of variation)

Cesarean section delivery varies significantly across each cluster. ICC indicated that 44.1% of the variation in ~~cesarean section~~CS delivery among ~~women~~mothers was attributed to community-level factors. PCV ~~(proportional change of variability)~~ in the final model ~~shows~~showed that 29.1% of the variation in ~~cesarean section~~CS delivery across communities was explained.

The median odds ratio confirmed that the variation in CS delivery was influenced by community-level factors. In the initial model (model zero), the median odds ratio for CS delivery was 9.2, indicating significant variation between communities (9.2 times higher than the reference or MOR=1). However, when all factors were added to the final model, the unexplained community variation decreased to an MOR of 7.9. This suggests that even after considering all factors, the effects of clustering remain statistically significant in the full models (Table 4).

Table 4 Model fitness and selection

Model	ICC	Variance	PCV	MOR	LLR	AIC	BIC
Model 0	44.1%	2.3	Reference	9.2	-1202	2408	2421
Model 1		1.67	27.39%	8.01	-639	1423	1514
Model 2	32.07%	1.7	26.09%	8.08	-983	1993	2079
Model 3	31.2%	1.63	29.1%	7.9	-632	1320	1479

Discussion

This study used the 2019 ~~MEDHS~~mini EDHS data to analyze the ~~spatial distribution, prevalence of CS~~ geospatial pattern and ~~determine the~~ individual and community-level factors ~~that associated with~~of CS delivery in Ethiopia. The finding of this study indicates that CS prevalence was 5.44% in Ethiopia. The finding is ~~almost~~ similar to study conducted in Mozambique (4.7~~%)(22),~~%) [9],

Formatted: Header

Formatted: Space After: 0 pt, Line spacing: 1.5 lines

Formatted: Font: 12 pt, Bold

Formatted: Line spacing: 1.5 lines

Formatted Table

Formatted: Line spacing: 1.5 lines

Formatted: Footer

and Uganda (5.22~~%)(30).~~%) [31]. However, this prevalence is less than the research conducted in Nepal (9%) ~~(31),~~[32], Egypt (40.1~~%)(32),~~%) [33], Bangladesh (63%) ~~(33),~~[34], and China (37%) ~~(34).~~[35]. This is slightly increased from the previous EDHS ~~study result~~report in ~~Ethiopia(18, 19).~~2011 (0.9%) [36], and 2016 (1.9%). This indicates that CS availability has slightly improved, however, it is still below the WHO recommendation which indicates unmet need as per the WHO statement ~~(4, 6)~~[4, 5]. The unmeet rate of CS delivery and geographical disparities in the region of Ethiopia in the current study could be attributed to ~~a number of~~many factors, including inadequate and/or sparse availability of ~~emergence~~emergency obstetrics services across the nation, inadequate equipment and medication in obstetric emergency centers, a shortage of skilled birth attendants and services, long travel distances, and unsuitable terrain that could provide a substantial geographic barrier to emergency obstetric treatment~~(35).~~ [37]. Related to factors, maternal age, sex of the household, religion, educational status, types of birth from individual level variable and, region from community-level variable had a statistical association with ~~cesarean section~~CS delivery. The probability of having cesarean section delivery was high among participants who are in the age group 25-34 and 35-49 as compared to ~~women~~mothers in the age group. This finding is supported the by study in Vietnam ~~(36),~~[38], Egypt~~(32),~~ [33], and Mozambique~~(22).~~ [9]. The reason might be ~~since~~that the risk of delivery problems as well as the chance of premature birth and infant death increased as ~~women~~mothers got older, excessive bleeding during labor, prolonged labor lasting more than 20 hours, and dysfunctional labor that does not progress to the next stage. Furthermore, diabetes and hypertension during pregnancy were more common in older pregnant ~~women~~mothers; this may lead to increased delivery by CS ~~(37).~~[39]. The odds of ~~women~~mothers who live with a female household head ~~had~~were more likely ~~delivered~~to deliver by ~~cesarean section~~ CS than ~~women~~mothers who live with ~~female~~a male household head. ~~Theirs~~There is no evidence to support this finding ~~(14-16, 34, 38)~~[19-21, 35, 40]. Muslim religion~~-~~-follower ~~women~~mothers were less likely ~~delivered~~to deliver by ~~cesarean section~~ CS delivery than Orthodox religion~~-~~-follower ~~women~~mothers. This finding is supported by the study conducted in Nigeria~~(21) . Women~~ [8]. Mothers who were living in Addis Ababa and Dire ~~Dawa~~Dewa were; more likely to have ~~cesarean section~~CS delivery as compared to ~~women~~mothers who ~~live~~lived in the Tigray region~~(39).~~ [41]. The odds of ~~cesarean section~~CS delivery among ~~women~~mothers who had completed primary, secondary, or higher education ~~had~~were more likely than ~~women~~mothers who had not ~~attend~~attended education. This finding is supported by the study

Formatted: Header

Formatted: Font color: Auto

Formatted: Font color: Auto

Formatted: Footer

conducted ~~at~~by global, ~~regional~~ and national ~~representative~~representatives of 150 countries using secondary data ~~from 1990–2014, Ethiopia, Vietnam, Tanzania (2, 23, 34, 36, 40, 41)~~[2, 35, 38, 42-44]~~,~~. The possible reason for a higher degree of education enhances the possibility of CS delivery might be ~~women~~mothers who ~~had~~have received education aware of the costs and benefits of using maternity services and also have more confidence and self-reliance for any decision provided by a ~~health care~~healthcare provider, which in turn, may raise the chance that could get CS service, besides, evidence from health literature demonstrates the fact that health literacy is linked to educational achievement could lead to believe that highly educated ~~women~~mothers are more likely to seek health services such as CS delivery than their no educated counterparts ~~(42).~~[45]. In this finding, multiple births (twin pregnancies) were significant factors for CS delivery. ~~Few studies support this finding,~~This study is supported by a study conducted in Egypt ~~(32) [9], a study in~~[33] and Nigeria~~(21).~~ [8]. The possible reason for this could be multiple births (twin pregnancies) have been linked to obstetric complications like preterm labor, premature rupture of membranes, malposition, and male presentation of the fetus. It also increased risks of complications to the mother like gestational diabetes, gestational hypertension, preeclampsia, and intrahepatic cholestasis, all of which may increase the likelihood of giving birth via CS delivery. The strength of research was using nationally representative datasets, which gives sufficient statistical power and the study estimates completed after the data had been weighted to allow for probability sampling and nonresponse to make it representative at national and regional levels. The limitation of this study was some factors that affecting CS delivery, like birth weight, maternal weight and height to generate BMI were incomplete in the 2019 mini EDHS data.

Conclusion

~~The prevalence of CS is slightly increased in~~In Ethiopia, ~~and it is~~CS deliveries remain below ~~WHO recommendation. The spatial distribution of cesarean section delivery in Ethiopia reveals notable variations across different regions. Hotspot areas, particularly~~the recommended World Health Organization rates with distinct geospatial variation. Geographically, CS prevalence is concentrated in urban ~~regions~~areas like Addis Ababa and Diredewa, ~~Harerie, and~~as well as in parts of the Somali and Oromia~~, indicate specific areas with higher prevalence.~~ regions. Maternal age, ~~sex of the~~female household head, Muslim religion, higher educational status, ~~types of birth from~~and multiple births at the individual level ~~factor~~, and~~,~~ region ~~from community-level factor had~~

Formatted: Header
Formatted: Font: +Body (Calibri), 11 pt
Formatted: Font: +Headings (Cambria), 16 pt
Formatted: Heading 1, Left, Line spacing: 1.5 lines
Formatted: Font: +Headings (Cambria), 16 pt, Not Bold
Formatted: Line spacing: 1.5 lines
Formatted: Footer

~~a statistical association with cesarean section delivery. Therefore the implementation of cesarean section delivery should be continuing to ideal rate~~at the community level were factors of CS delivery ~~as per WHO recommendation~~in Ethiopia. Therefore, efforts should be geared towards tailoring strategies and interventions to ensure fair access to CS delivery lines with WHO recommendations, especially in the region where CS delivery rates are low in Tigray, Amhara, Afar, and Benishangul Gumze regions.

Acknowledgments: The authors are sincerely grateful to the Demographic Health Survey (DHS) program for ~~providing~~allowing us to use the Ethiopian mini DHS dataset through their archives (archive@dhsprogram.com).

~~**Author Contributions:** AE contributed soft wear and data analysis. All author contributed in conceptualization, methodology writing, writing the original draft, AE: do formal analysis using software, AE, MA,YT, NK: do writing – review & editing:~~

List of Abbreviation: AIC: Akakian information Criteria, AOR: Adjusted odds ratio, ANC: Antenatal care, BIC: Bayesian information Criteria, COR~~-~~: Crud odds ratio, EA: Enumeration Area~~-~~, ICC: Intra-Class Correlation, MEDHS: Mini Demographic and Health Survey, MOR: Median Odds Ratio, PCV: Proportional Changes of Variance, SNNP: South Nation

Reference

1. Jauniaux, E~~.~~, and W. Grobman~~ W.~~, *Textbook of caesarean section*. 2016: Oxford University Press~~; 2016~~.
2. Boerma, T~~, Ronsmans C, Melesse DY, Barros AJ, Barros FC, Juan L,~~, et al~~.~~, *Global epidemiology of use of and disparities in caesarean sections.* The Lancet~~.~~, 2018~~;~~. **392**(10155): p. 1341-~~8~~1348.
3. Batieha, A~~, Al-Daradkah S, Khader Y, Basha A, Sabet F, Athamneh T,~~, et al~~.~~, *Cesarean section: incidence, causes, associated factors and outcomes: a national prospective study from Jordan.* Gynecol Obstet Case Rep~~.~~, 2017~~;~~. **3**(3): p. 55.
4. WHO~~.~~, *WHO statement on caesarean section rates.* Sexual and Reproductive Health and Research~~.~~, 2015.

Formatted: Header
Formatted: Font: Bold
Formatted: Line spacing: 1.5 lines
Formatted: Font: +Headings (Cambria), 16 pt
Formatted: Heading 1, Left, Line spacing: 1.5 lines
Formatted: Font: +Headings (Cambria), 16 pt, Not Bold
Formatted: EndNote Bibliography, Left, Indent: Left: 0", Hanging: 0.5", Line spacing: 1.5 lines
Formatted: Font: Calibri
Formatted: Font: Calibri
Formatted: Font: Calibri
Formatted: Font: Calibri
Formatted: Font: Calibri, Italic
Formatted: Font: Calibri
Formatted: Font: Calibri
Formatted: Font: Calibri
Formatted: Font: Calibri
Formatted: Font: Calibri
Formatted: Font: Calibri, Italic
Formatted: Font: Calibri
Formatted: Font: Calibri
Formatted: Font: Calibri, Bold
Formatted: Font: Calibri
Formatted: Font: Calibri
Formatted: Font: Calibri
Formatted: Font: Calibri
Formatted: Font: Calibri
Formatted: Font: Calibri
Formatted: Font: Calibri, Italic
Formatted: Font: Calibri
Formatted: Font: Calibri
Formatted: Font: Calibri, Bold
Formatted: Font: Calibri
Formatted: Font: Calibri
Formatted: Font: Calibri
Formatted: Font: Calibri, Italic
Formatted: Font: Calibri
Formatted: Font: Calibri
Formatted: Footer

~~5. Betrán AP, Ye J, Moller A-B, Zhang J, Gülmezoglu AM, Torloni MR.~~5. Betrán, A.P., et al., *WHO statement on caesarean section rates.* Bjog, 2016. **123**(5): p. 667.

6. Betrán, A.P., et al., *The increasing trend in caesarean section rates: global, regional and national estimates: 1990-2014.* PloS one~~.~~, 2016~~;~~. **11**(2): p. e0148343.

~~6. Betrán AP, Torloni MR, Zhang J J, Gülmezoglu A, Aleem H, Althabe F,~~7. Rockville, M., USA: GSS, GHS, and ICF International, *Ghana demographic and health survey 2014.* Ghana Statistical Service GHS, ICF International (2015), 2014.

8. Adewuyi, E.O., et al~~. WHO statement on caesarean.~~, *Cesarean delivery in Nigeria: prevalence and associated factors—a population-based cross-sectional study.* BMJ open, 2019. **9**(6): p. e027273.

9. Long, Q., et al., *Caesarean section rates~~. Bjog.~~ in Mozambique.* BMC pregnancy and childbirth, 2015. **15**(1): p. 1-9.

10. E, E., *demographic and health survey 2000: key indicators report. .* The DHS Program ICF:, 2000. **206.**

11. E, E., *demographic and health survey 2016~~;123(5):667~~: key indicators report.* The DHS Program ICF, 2016. **363: 364.**

~~7. Ye J, Zhang J, Mikolajczyk R, Torloni MR, Gülmezoglu A, Betran A.~~12. Ye, J., et al., *Association between rates of caesarean section and maternal and neonatal mortality in the 21st century: a worldwide population-based ecological study with longitudinal data.* BJOG: An International Journal of Obstetrics & Gynaecology~~.~~, 2016~~;~~. **123**(5): p. 745-~~53~~753.

~~8~~13. Gjonej, R~~, Poloska A, Keta M, Delija Z, Zyberaj F, Bezhani V,~~., et al~~.~~., *The reasons of rising trend of cesarean section rate year after year. A retrospective study.* International Journal of Nursing and midwifery~~.~~, 2015~~;~~. **7**(1): p. 9-15.

~~9~~14. Harrison ~~MS,~~ M.S. and R.L. Goldenberg ~~RL.~~, *Cesarean section in sub-Saharan Africa.* Maternal health, neonatology and perinatology~~.~~, 2016~~;~~. **2**(1): p. 1-10.

~~10. Gebremedhin S. Trend and socio-demographic differentials of Caesarean section rate in Addis Ababa, Ethiopia: analysis based on Ethiopia demographic and health surveys data. Reproductive health. 2014;11(1):1-6.~~

Formatted: Header

Formatted: Font: Calibri

Formatted: Font: Calibri, Italic

Formatted: EndNote Bibliography, Left, Indent: Left: 0", Hanging: 0.5", Line spacing: 1.5 lines

Formatted: Font: Calibri

Formatted: Font: Calibri

Formatted: Font: Calibri, Bold

Formatted: Font: Calibri

Formatted: Font: Calibri

Formatted: Font: Calibri

Formatted: Font: Calibri, Italic

Formatted: EndNote Bibliography, Left, Indent: Left: 0", Hanging: 0.5", Line spacing: 1.5 lines

Formatted: Font: Calibri, Italic

Formatted: Font: Calibri, Bold

Formatted: Font: Calibri

Formatted: Font: Calibri, Italic

Formatted: Font: Calibri

Formatted: Font: Calibri

Formatted: Font: Calibri, Bold

Formatted: Font: Calibri

Formatted: Font: Calibri

Formatted: Font: Calibri

Formatted: Font: Calibri

Formatted: Font: Calibri

Formatted: Font: Calibri

Formatted: Font: Calibri

Formatted: Font: Calibri, Italic

Formatted: Font: Calibri

Formatted: Font: Calibri

Formatted: Font: Calibri, Bold

Formatted: Font: Calibri

Formatted: Font: Calibri

Formatted: Font: Calibri

Formatted: Font: Calibri

Formatted: Font: Calibri

Formatted: Font: Calibri, Italic

Formatted: Font: Calibri

Formatted: Font: Calibri

Formatted: Font: Calibri, Bold

Formatted: Font: Calibri

Formatted: Font: Calibri

Formatted: Footer

~~11. Yisma E, Smithers LG, Lynch JW, Mol BW. Cesarean section in Ethiopia: prevalence and sociodemographic characteristics. The Journal of Maternal-Fetal & Neonatal Medicine. 2019;32(7):1130-5.~~

~~12~~15. Ayalew~~ M, Mengistie B, Dheressa~~, M~~, Demis A.~~., et al., *Magnitude of cesarean section delivery and its associated factors among mothers who gave birth at public hospitals in Northern Ethiopia: Institution-based cross-sectional study*. Journal of multidisciplinary healthcare~~.~~, 2020: p. 1563-~~71~~1571.

~~13~~16. Gutema, H~~.~~. and A. Shimye~~ A.~~, *cesarean section and associated factors at mizan aman general hospital, southwest Ethiopia*. J Gynecol Obstet~~.~~, 2014~~;~~. **2**(3): p. 37-41.

~~14~~17. Gebremedhin, S., *Trend and socio-demographic differentials of Caesarean section rate in Addis Ababa, Ethiopia: analysis based on Ethiopia demographic and health surveys data.* Reproductive health, 2014. **11**(1): p. 1-6.

18. Yisma, E., et al., *Cesarean section in Ethiopia: prevalence and sociodemographic characteristics.* The Journal of Maternal-Fetal & Neonatal Medicine, 2019. **32**(7): p. 1130-1135.

19. Sakae~~ TM,~~, T.M., P.F. Freitas~~ PF,~~, and E. d'Orsi~~ E.~~, *Factors associated with cesarean section rates in a university hospital*. Revista de saude publica~~.~~, 2009~~;~~. **43**: p. 472-~~80~~480.

~~15~~20. Taye~~ MG, Nega F, Belay MH, Kibret S, Fentie Y, Addis WD,~~, M.G., et al~~.~~., *Prevalence and factors associated with caesarean section in a comprehensive specialized hospital of Ethiopia: a cross-sectional study; 2020.* Annals of Medicine and Surgery~~.~~, 2021~~;~~. **67**: p. 102520.

~~16~~21. Tsega, F~~, Mengistie B, Dessie Y, Mengesha M.~~., et al., *Prevalence of cesarean section in urban health facilities and associated factors in Eastern Ethiopia: hospital based cross sectional study*. J Preg Child Health~~.~~, 2015~~;~~. **2**(3): p. 169-73.

~~17. Hernández-Vásquez A, Chacón-Torrico H, Bendezu-Quispe G. Differences in the prevalence of cesarean section and associated factors in private and public healthcare systems in Peru. Sexual & Reproductive Healthcare. 2020;26:100570.~~

Formatted: Header

Formatted ...

Formatted: EndNote Bibliography, Left, Indent: Left: 0", Hanging: 0.5", Line spacing: 1.5 lines

Formatted ...

Formatted: EndNote Bibliography, Left, Indent: Left: 0", Hanging: 0.5", Line spacing: 1.5 lines

Formatted ...

Formatted ...

Formatted ...

Formatted: Footer

18. E E. demographic and health survey 2000: key indicators report. . The DHS Program ICF:. 2000;206.

19. E E. demographic and health survey 2016: key indicators report. The DHS Program ICF. 2016;363: 364.

20. Rockville M, USA: GSS, GHS, and ICF International. Ghana demographic and health survey 2014. Ghana Statistical Service GHS, ICF International (2015). 2014.

21. Adewuyi EO, Auta A, Khanal V, Tapshak SJ, Zhao Y. Cesarean delivery in Nigeria: prevalence and associated factors—a population-based cross-sectional study. BMJ open. 2019;9(6):e027273.

22. Long Q, Kempas T, Madede T, Klemetti R, Hemminki E. Caesarean section rates in Mozambique. BMC pregnancy and childbirth. 2015;15(1):1-9.

23. Tsegaye H, Desalegne B, Wassihun B, Bante A, Debalkie M, Yeheyis T. Prevalence and associated factors of caesarean section in Addis Ababa hospitals, Ethiopia. Pan African Medical Journal. 2019;34(1).

24. tw, b.., *1. bank tw (Jul 15, 2021) The world bank Ethiopia.* 2021.

23. Tsai, P.-J., et al., *Spatial autocorrelation analysis of health care hotspots in Taiwan in 2006.* BMC Public Health, 2009. **9**: p. 1-13.

24. 25. Kulldorff, M.., *A spatial scan statistic.* Communications in Statistics-Theory and methods., 1997;., **26**(6): p. 1481-961496.

25. 26. Jung, I., M. Kulldorff M., and A.C. Klassen AC., *A spatial scan statistic for ordinal data.* Statistics in medicine., 2007;., **26**(7): p. 1594-6071607.

26. 27. Pezzulo, C, Nilsen K, Carioli A, Tejedor-Garavito N, Hanspal SE, Hilber T,., et al.., *Geographical distribution of fertility rates in 70 low-income, lower-middle-income, and upper-middle-income countries, 2010–16: a subnational analysis of cross-sectional surveys.* The Lancet Global Health., 2021;., **9**(6): p. e802-e12e812.

Formatted: Header

Formatted: Font: +Body (Calibri)

Formatted: EndNote Bibliography, Left, Indent: Left: 0", Hanging: 0.5", Line spacing: 1.5 lines

Formatted: Font: Calibri

Formatted: Font: Calibri

Formatted: Font: Calibri

Formatted: Font: Calibri, Italic

Formatted: Font: Calibri

Formatted: EndNote Bibliography, Left, Indent: Left: 0", Hanging: 0.5", Line spacing: 1.5 lines

Formatted: Font: Calibri

Formatted: Font: Calibri

Formatted: Font: Calibri

Formatted: Font: Calibri, Italic

Formatted: Font: Calibri

Formatted: Font: Calibri

Formatted: Font: Calibri, Bold

Formatted: Font: Calibri

Formatted: Font: Calibri

Formatted: Font: Calibri

Formatted: Font: Calibri

Formatted: Font: Calibri

Formatted: Font: Calibri

Formatted: Font: Calibri

Formatted: Font: Calibri, Italic

Formatted: Font: Calibri

Formatted: Font: Calibri

Formatted: Font: Calibri, Bold

Formatted: Font: Calibri

Formatted: Font: Calibri

Formatted: Font: Calibri

Formatted: Font: Calibri

Formatted: Font: Calibri

Formatted: Font: Calibri

Formatted: Font: Calibri

Formatted: Font: Calibri, Italic

Formatted: Font: Calibri

Formatted: Font: Calibri

Formatted: Font: Calibri, Bold

Formatted: Font: Calibri

Formatted: Font: Calibri

Formatted: Font: Calibri

Formatted: Footer

~~28~~27. Pezzulo, C~~, Nilsen K, Carioli A, Tejedor Garavito N, Hanspal SE, Hilber T~~., et al~~.~~., *The geography of fertility rates in low and middle-income countries: a subnational analysis of cross-sectional surveys from 70 countries, 2010-16.* The Lancet Global Health~~.~~, 2021~~;~~. **9**(6): p. E802-~~E12~~E812.

~~29. Kakujaha-Matundua O.~~28. Gribov, A. and K. Krivoruchko, *Empirical Bayesian kriging implementation and usage.* Science of the Total Environment, 2020. **722**: p. 137290.

29. Austin, P.C. and J. Merlo, *Intermediate and advanced topics in multilevel logistic regression analysis.* Statistics in medicine, 2017. **36**(20): p. 3257-3277.

30. Kakujaha-Matundua, O., *Towards Identifying and estimating public expenditure on gender-based violence in Namibia.* SOCIAL SCIENCES DIVISION (SSD~~).~~), 2015~~;~~. **110**.

~~30.~~ 31. Statistics ~~UBO,~~, U.B.O. and I. MACRO ~~I.~~, *Uganda Demographic and Health Survey.* Kampala: Uganda Bureau of Statistics~~.~~, 2011.

32. ~~31.~~ Acharya, K~~.~~. and Y.R. Paudel ~~YR.~~, *Trend and sociodemographic correlates of cesarean section utilization in Nepal: evidence from demographic and health surveys 2006-2016.* BioMed research international~~.~~, 2021~~;~~. **2021**.

~~32~~33. Al Rifai ~~RH.~~, R.H., *Trend of caesarean deliveries in Egypt and its associated factors: evidence from national surveys, 2005–2014.* BMC pregnancy and childbirth~~.~~, 2017~~;~~. **17**(1): p. 1-14.

~~33.~~ 34. Haider ~~MR, Rahman MM, Moinuddin~~, M~~, Rahman AE, Ahmed S, Khan MM.~~.R., et al., *Ever-increasing Caesarean section and its economic burden in Bangladesh.* PloS one~~.~~, 2018~~;~~. **13**(12): p. e0208623.

35. ~~34.~~ Zhang, J~~, Zhang Y, Ma Y, Ke Y, Huo S, He L,~~., et al~~.~~., *The associated factors of cesarean section during COVID-19 pandemic: a cross-sectional study in nine cities of China.* Environmental health and preventive medicine~~.~~, 2020~~;~~. **25**(1): p. 1-7.

~~35~~36. *Demographic EE (2011) Health Survey 2011. Central Statistical Agency. Adis Abab, Ethiopia.*

37. Fesseha, N~~, Getachew A, Hiluf M, Gebrehiwot Y, Bailey P.~~., et al., *A national review of cesarean delivery in Ethiopia.* International Journal of Gynecology & Obstetrics~~.~~, 2011~~;~~. **115**(1): p. 106-~~11~~111.

Formatted: Header

Formatted ...

Formatted: EndNote Bibliography, Left, Indent: Left: 0", Hanging: 0.5", Line spacing: 1.5 lines

Formatted ...

Formatted: Font: Calibri

Formatted ...

Formatted ...

Formatted ...

Formatted: Font: Calibri

Formatted ...

Formatted ...

Formatted: EndNote Bibliography, Left, Indent: Left: 0", Hanging: 0.5", Line spacing: 1.5 lines

Formatted ...

Formatted: Footer

Formatted: Header

3638. de Loenzien, M, Schantz C, Luu BN, Dumont A., et al., *Magnitude and correlates of caesarean section in urban and rural areas: A multivariate study in Vietnam.* PloS one., 2019;. **14**(7): p. e0213129.

3739. Rydahl, E, Declercq E, Juhl M, Maimburg RD., et al., *Cesarean section on a rise—Does advanced maternal age explain the increase? A population register-based study.* PloS one., 2019;. **14**(1): p. e0210655.

3840. Yaya, S, Uthman OA, Amouzou A, Bishwajit G., et al., *Disparities in caesarean section prevalence and determinants across sub-Saharan Africa countries.* Global health research and policy., 2018;. **3**: p. 1-9.

3941. Mekonnen ZA, Lerebo WT, Gebrehiwot TG, Abadura SA., Z.A., et al., *Multilevel analysis of individual and community level factors associated with institutional delivery in Ethiopia.* BMC research notes., 2015;. **8**(1): p. 1-9.

40. Beyene MG, Zemedu TG, Gebregiorgis AH, Ruano AL, Bailey PE.42. Beyene, M.G., et al., *Cesarean delivery rates, hospital readiness and quality of clinical management in Ethiopia: national results from two cross-sectional emergency obstetric and newborn care assessments.* BMC pregnancy and childbirth., 2021;. **21**: p. 1-11.

4143. Tsegaye, H., et al., *Prevalence and associated factors of caesarean section in Addis Ababa hospitals, Ethiopia.* Pan African Medical Journal, 2019. **34**(1).

44. Shibre, G, Zegeye B, Ahinkorah BO, Keetile M, Yaya S., et al., *Magnitude and trends in socio-economic and geographic inequality in access to birth by cesarean section in Tanzania: evidence from five rounds of Tanzania demographic and health surveys (1996–2015).* Archives of Public Health., 2020;. **78**: p. 1-10.

4245. Logan RA, Wong WF, Villaire M, Daus G, Parnell TA, Willis E, R.A., et al., *Health literacy: A necessary element for achieving health equity.* NAM perspectives., 2015.

Table 2 **Socio-demographic character's of mother's who gave birth by cesarean section in Ethiopia Using 2019 MEDHS.**

Formatted: EndNote Bibliography, Left, Indent: Left: 0", Hanging: 0.5", Line spacing: 1.5 lines

Formatted: Footer

Variable	Category	Cesarean section delivery			
		No	%	Yes	%
Age category	15-24	124	52	51	48
	25-34	2512	93.8	165	6.2
	35-49	1199	6.6	85	93.4
Sex of household	Male	4538	95	237	5
	Female	688	91.6	63	8.4
Place of residence	Urban	1229	89.9	138	11.1
	Rural	3998	96.1	162	3.9
Religion	Christian	3106	93.1	232	6.9
	Muslim	2120	96.8	69	3.2
Region	Tigray	346	93.3	25	6.7
	Afar	84	96.5	3	3.5
	Amhara	972	92.57	78	7.43
	Oromia	2120	95.9	90	4.1
	Somali	404	99.1	4	0.98
	Benshngul gumz	63	94.03	4	5.97
	SNNP	1054	95.38	51	4.62
	Gambella	24	96	1	4
	Hareri	15	88.24	2	11.76
	Addis Abeba	118	75.64	38	24.36
	Dredewa	25	83.3	5	16.7
Martital status	Union	2951	91.5	274	8.5
	Not union	276	91.1	27	8.9
Educational status	Cannot read and write	2875	97.1	86	2.9
	Primary education	1875	93.75	125	6.25
	secondary education	1832	97.23	52	2.77

Formatted: Header

Formatted: Footer

	Higher education	362	90.5	38	9.5
Wealth index	Poorest	1300	98.4	21	1.6
	Poorer	1154	96.33	44	3.67
	Middle	997	95.5	47	4.5
	Richer	915	95.3	45	4.7
	Richest	861	85.8	144	14.3

Table 4 The multilevel analysis of associated factors of cesarean section in Ethiopia Using 2019 MEDHS.

Variable	Category	CS delivery		Bivariable		Model (null) 0	Model 1	Model 2	Model 3
		No	Yes	P value	95%COR	ICC=4 4.1%	95%AOR	95%AOR	95%AOR
Age category	15-24	124	51		Reference		Reference		Reference
	25-34	2512	165	0.022	1.4(1.05-2.00)		1.9(1.3-3.07)		**1.98(1.3-3.1)**
	35-49	1199	85	0.003	1.73(1.21-2.47)		3.6(2.2-6.0)		**3.7(2.2-6.1)**
Sex of household	Male	4538	237		Reference		Reference		Reference
	Female	688	63	0.00	1.73(1.5-2.3)		1.8(1.1-2.9)		**1.9(1.2-3.1)**
Place of residence	Urban	1229	138		Reference			Reference	Reference
	Rural	3998	162	0.000	0.36(0.3-0.5)			0.3(0.14-0.5)	0.6(0.3-1.[illegible]
Religion	Christian	3106	232		Reference				Reference

Formatted: Header

Formatted Table

Formatted: Font: 11 pt

Formatted: Line spacing: 1.5 lines

Formatted: Font: 11 pt

Formatted: Line spacing: 1.5 lines

Formatted Table

Formatted: Font: 11 pt

Formatted: Line spacing: 1.5 lines

Formatted: Footer

	Muslim	2120	69	0.000	0.4(0.3-0.57)		0.4(0.23-0.69)		0.4(0.2-0.[illegible]
Region	Tigray	346	25		Reference		Reference	Reference	Reference
	Afar	84	3	0.153	0.36(0.1-1.5)			0.4(0.07-2.3)	2.4(0.3-2[illegible]
	Amhara	972	78	0.74	1.07(0.8-1.7)			1.4(0.5-3.7)	2.4(0.8-6.6)
	Oromia	2120	90	0.016	0.6(0.36-0.9)			0.5(0.2-1.4)	1.8(0.6-5.2)
	Somali	404	4	0.00	0.14(0.1-0.4)			0.13(0.03-0.6	0.6(0.05-4.33)
	Benshngul gumz	63	4	0.838	0.9(0.3-2.5)			0.9(0.2-4.2)	2.0(0.4-10.09)
	SNNP	1054	51	0.087	0.7(0.4-1.0)			1.0(0.4-2.7)	1.9(0.7-5.[illegible]
	Gambella	24	1	0.556	0.5(0.05-4.3)			0.4(0.03-5.03)	0.3(0.02-6.6[illegible]
	Hareri	15	2	0.83	1.2(0.2-7.36)			1.0(0.1-9.6)	2.9(0.2-38.4)
	Addis Abeba	118	38	0.000	4.3(2.4-7.36)			2.9(0.9-8.9)	**3.6(1.1-11.9)**
	Dredewa	25	5	0.093	2.4(0.9-7.2)			2.5(0.6-11.02	**7.0(1.23-40.7)**
Marital status	Union	2951	274		Reference		Reference		Reference
	Not union	276	27	0.01	1.7(1.1-2.6)		1.37(0.72-2.6)		1.3(0.7-2.[illegible]
Educational status	Cannot read and write	2875	86		Reference		Reference		Reference
	Primary education	1875	125	0.000	2.27(1.7-3.0)		1.68(1.07-2.6)		**1.6(1.07-2.7)**
	secondary education	1832	52	0.000	4.8(3.3-6.9)		2.3(1.3-4.12)		**2.4(1.3-4.25)**
	Higher education	362	38	0.000	7.9(5.2-12.6		2.2(1.07-4.4)		**2.06(1.03-4.2)**
	Poorest	1300	21		Reference		Reference		Reference

Formatted: Header
Formatted: Font: 11 pt, Not Bold
Formatted: Font: 11 pt
Formatted: Line spacing: 1.5 lines
Formatted: Font: 11 pt
Formatted: Line spacing: 1.5 lines
Formatted Table
Formatted: Font: 11 pt
Formatted: Line spacing: 1.5 lines
Formatted Table
Formatted: Font: 11 pt
Formatted: Line spacing: 1.5 lines
Formatted: Footer

Wealth index	Poorer	1154	44	0.002	2.33(1.4-3.9)		1.4(0.68-3.05)		1.3(0.6-2.[illegible]
	Middle	997	47	0.000	2.89(1.7-4.8)		0.79(0.4-1.7)		0.7(0.3-1.6)
	Richer	915	45	0.000	3.05(1.8-5.2)		0.9(0.4-1.9)		0.8(0.4-1.7)
	Richest	861	144	0.000	10.4(6.5-16.5)		1.3(0.5-2.6)		0.8(0.3-1.9)
Last birth by CS	Yes	43	25	0.000	1528.6(870-2684)				
	No	4842	281		Reference				
ANC Visit	No	11	1033		Reference		Reference		Reference
	Yes	272	2663	0.000	8.17(4.4-14.9		1.94(0.8-4.3)		1.9(0.8-4.2)
Types of birth	Single	325	5261		Reference		Reference		Reference
	Multiple	24	143	0.000	4.6(3.1-7.1)		8.01(3.35-19.6)		**8.1(3.4-19.74)**
Sex of New birth	Male	178	2791		Reference				Reference
	Female	171	2613	0.6	1.06(0.84-1.34)				
Place of delivery	Public health facility	283	2280		Reference		Reference		Reference
	Private hospital	66	243	0.013	1.68(1.2-2.5)		1.4(0.8-2.5)		1.3(0.6-2.4)

Key AOR= Adjusted odds ratio, COR crud odds ratio, ICC= Intra Class Correlation, CS= Cesarean Section, ANC Antenatal care, SNNP South Nation Nationalities and Peoples,

Formatted: Header

Formatted: Font: 11 pt

Formatted: Line spacing: 1.5 lines

Formatted Table

Formatted: Font: 11 pt

Formatted: Line spacing: 1.5 lines

Formatted: Font: 11 pt

Formatted: Line spacing: 1.5 lines

Formatted: Font: 11 pt

Formatted: Line spacing: 1.5 lines

Formatted Table

Formatted: Font: 12 pt

Formatted: Left, Line spacing: 1.5 lines

Formatted: Footer

Authors' Responses to Editor and Reviewers Comments

"Geospatial Patterns, Prevalence, and Individual and Community Levels Factors of Cesarean Section Deliveries in Ethiopia: A Spatial and Multilevel Analysis"

Dear Plos One Editors and Reviewers;

We are thankful for your constructive comments. We have looked at the comments and have revised our paper accordingly. We hope our paper improved as a result of incorporating the reviewer's and academic editor's comments and suggestions. Here are the authors' responses to the comments.

Please find for your kind consideration the following:

1. A revised manuscript with track changes.
2. A revised paper without tracked changes
3. A rebuttal letter that responds to each point raised by the academic editor and reviewer.

The point by point responses of authors are written by hoping these changes would meet with your favorable consideration, we are happy to hear if there are more comments and suggestions. Please do not hesitate to let us know if you have any questions.

Yours Sincerely

Mr. Abel Endawkie Correspondence Author

Department of Epidemiology and Biostatistics School of Public Health College of Medicine and Health Science Wollo University Dessie Ethiopia

Tel. 251935459310 Email address abelendawkie@gmail.com

We have tried our best to improve it accordingly:

Please revise the manuscript.

Journal requirements:

When submitting your revision, we need you to address these additional requirements.

1. Please ensure that your manuscript meets PLOS ONE's style requirements, including those for file naming. The PLOS ONE style templates can be found at

https://journals.plos.org/plosone/s/file?id=wjVg/PLOSOne_formatting_sample_main_body.pdf and

https://journals.plos.org/plosone/s/file?id=ba62/PLOSOne_formatting_sample_title_authors_affiliations.pdf

Response 1: We revised our manuscript based on Plos one submission guidelines (manuscript, title, table, figure and reference format).

2. We noticed you have some minor occurrence of overlapping text with the following previous publication(s), which needs to be addressed:

- https://www.researchsquare.com/article/rs-1386764/v1

- https://doi.org/10.1371/journal.pone.0282951

- https://bmcwomenshealth.biomedcentral.com/articles/10.1186/s12905-021-01484-1

Response 2: We would like to thank you kindly. After careful review, we made the necessary revisions and corrections.

3. We suggest you thoroughly copyedit your manuscript for language usage, spelling, and grammar. If you do not know anyone who can help you do this, you may wish to consider employing a professional scientific editing service.

Response 3: We are grateful to thank you for your kindness and response. We have tried to correct your the languge thoroughly.

3. Your ethics statement should only appear in the Methods section of your manuscript. If your ethics statement is written in any section besides the Methods, please delete it from any other section.

Response 2: We have prepared it.

Point-by-point response of Authors for editors' and reviewers' comment

Editor's Comments to the Authors

Additional Editor Comments:

The authors are requested to address the reviewers' comments carefully and in detail

Response: We are grateful to thank you for your kindness and response. We tried to correct it thoroughly.

Dear Editors: We would like to notify you that, for more appropriate understanding of the paper we have revised the title to **"Geospatial Patterns, and Individual and Community Levels Factors of Cesarean Section Deliveries in Ethiopia: A Spatial and Multilevel Analysis** "

Response: We are grateful to thank you for your kindness and response. We have tried to correct your concern and suggestion thoroughly.

Reviewer Comment to the Authors

<u>Abstract section</u>

Comment 1 Abstract result section: this is presented differently, better to use commen words.

Response 1: We sincerely appreciate your thoughtful feedback. After careful review, we made the necessary revisions and corrections.

Comment 2 Abstract conclusion section**:** The previous result no include the prevalence over time? So increase aspect is not clear please review.

Response 2: We extend our sincere gratitude for your insightful comment. We found it particularly valuable during the writing process of our work. We would like to sorry for making confuse in understanding. After careful review, we made the necessary revisions and corrections.

Comment 3: Abstract conclusion section**:** This sentence is not complete

Response 3: We sincerely appreciate your thoughtful comment. In response to your concern, since we found it particularly valuable during the writing process of our work and we would like to sorry for making confuse in understanding. After careful review, we made the necessary revisions and corrections.

<u>Introduction section</u>

Comment 4. Lesthan 5% people . It is not clear (people or pregnant women)

Response 4: We would like to thank you kindly for your comment. We had revised and corrected it thoroughly. Our interest is to say, according to the WHO statement, CS prevalence less than 5% among reproductive age mothers in the country or specific community indicates unmet need, whereas a prevalence of CS greater than 15% among reproductive age mothers might put the mother and new birth at risk.

Comment 5: population studies it is not clear?

Response 5: We would like to thank you kindly. We found it particularly valuable during the writing process of our work and we would like to sorry for making confuse in understanding. After careful review, we made the necessary revisions and corrections.

Comment 6: what is negative if CS rate lower infant death? It is not clear

Response 6: We express our gratitude for your insightful comment. We found it particularly valuable scientific plausibility of the paper and we would like to sorry for making confuse in understanding. After careful review, we made the necessary revisions and corrections.

Comment 7: Which WHO survey?

Response 7: First and foremost, we express our sincere gratitude for your intriguing idea and thoughtful questions. We have gained valuable insights from it. After careful review, we made the necessary revisions and corrections.

Comment 8: line 70 Explaine more.

Response 8: We sincerely appreciate your thoughtful comment. After careful review, we made the necessary revisions and corrections.

Comment 9: line 75 Start this in new paragraph given that is different topic.

Response 9: We express our sincere gratitude for your insightful comment. We have gained valuable insights from it. After careful review, we made the necessary revisions and corrections.

Comment 10. Line 77 Does it mean the rate of CS decreasing?

Response 10: We express our sincere appreciation for your insightful comment. We would like to sorry for making confuse in understanding. The rate of CS delivery in Ethiopia is increasing from 0.7 to 1.9 in 2000 to 2016 EDHS.

Commentm 11: line 80 write the numper in parenthesis after country name.

Response 11: We extend our sincere appreciation for your thoughtful comment. In response to your query, we made the necessary revisions and corrections..

Comment 12: introduce the mini EDHS data.

Response 12: We express our sincere appreciation for your insightful comment. Following your feedback, we have made revisions and incorporated your comment in manuscript.

<u>Method section</u>

Comment13: you have to explaine it first MEDHS?

Response 13: First and foremost, we express our sincere gratitude for your intriguing idea and thoughtful questions. In response to your query, we have made revisions and incorporated your comment in manuscript.

Comment 14: please explain more your sampling and sample size determination.

Response 14: We would like to thank you kindly for your deep comment. In response to your query, we have made revisions and incorporated your comment in manuscript. Please look the sampling procedure depiceted in figure 1 in detail

Comment 15. Line 120: no need of parenthesis

Response 15: We sincerely appreciate your thoughtful comment. The issue you raised is indeed intriguing. we have made revisions and incorporated your comment in manuscript.

Comment 16. Line 61: In which table please spacify

Response 16: We sincerely appreciate your thoughtful comment. We provided in table 2 and 3 Please look at table a 2 and 3

Comment 17: Line 131 please explaine more types of spatial statistics

Response 17: We sincerely appreciate your thoughtful comment. We have carefully reviewed and made the necessary corrections.

Comment 18. Please write the equation in new line

Response 18: We sincerely appreciate your thoughtful comment. We have carefully reviewed and made the necessary corrections.

Comment 19. Standar error never more than ±2

Response 19: We sincerely appreciate your thoughtful comment. Multicolinarity in logistic analysis is not estimated using variance inflaction factor (VIF). In this we consider the standard error of the variable. If the standard is below -2 and above 2 it indicates multiconiarity but it is does not mean that standard error never less than -2 or greater 2.

Result Section

Comment 20. Please edit more and please specify the delivery year before the DHS survey.

Response 20: We sincerely appreciate your thoughtful comment. We have carefully reviewed and made the necessary corrections.

Discusion Section

Comment 21. What was the previous CS delivery in Ethiopia please write

Response 21: We would like to thank you kindly for your deep comment. We have carefully reviewed and made the necessary corrections.

We feel all of you may understand our feelings and interests.

We are thankful for all!!
