# Supplementary material for: Geospatial patterns, and individual and community levels factors of cesarean section deliveries in Ethiopia: A spatial and multilevel analysis
Source: PLoS One. 2024 Aug 27;19(8):e0306052. doi: 10.1371/journal.pone.0306052 (PMC11349178; doi:10.1371/journal.pone.0306052)
Supplement: Attachment — Submitted filename: Authors Response to Reviewers.docx [file pone.0306052.s002.docx]

**Authors’ Responses to Editor and Reviewers Comments**

“**Geospatial Patterns, Prevalence, and Individual and Community Levels Factors of Cesarean Section Deliveries in Ethiopia: A Spatial and Multilevel Analysis”**

Dear Plos One Editors and Reviewers;

We are thankful for your constructive comments. We have looked at the comments and have revised our paper accordingly. We hope our paper improved as a result of incorporating the reviewer’s and academic editor’s comments and suggestions. Here are the authors’ responses to the comments.

Please find for your kind consideration the following:

1. A revised manuscript with track changes.
2. A revised paper without tracked changes
3. A rebuttal letter that responds to each point raised by the academic editor and reviewer.

The point by point responses of authors are written by hoping these changes would meet with your favorable consideration, we are happy to hear if there are more comments and suggestions. Please do not hesitate to let us know if you have any questions.

Yours Sincerely

Mr. Abel Endawkie Correspondence Author

Department of Epidemiology and Biostatistics School of Public Health College of Medicine and Health Science Wollo University Dessie Ethiopia

Tel. 251935459310	Email address abelendawkie@gmail.com

We have tried our best to improve it accordingly:

Please revise the manuscript.

Journal requirements:

When submitting your revision, we need you to address these additional requirements.

1. Please ensure that your manuscript meets PLOS ONE's style requirements, including those for file naming. The PLOS ONE style templates can be found at

<https://journals.plos.org/plosone/s/file?id=wjVg/PLOSOne_formatting_sample_main_body.pdf> and

<https://journals.plos.org/plosone/s/file?id=ba62/PLOSOne_formatting_sample_title_authors_affiliations.pdf>

**Response 1:** We revised our manuscript based on Plos one submission guidelines (manuscript, title, table, figure and reference format).

2. We noticed you have some minor occurrence of overlapping text with the following previous publication(s), which needs to be addressed:

- https://www.researchsquare.com/article/rs-1386764/v1

- https://doi.org/10.1371/journal.pone.0282951

- <https://bmcwomenshealth.biomedcentral.com/articles/10.1186/s12905-021-01484-1>

**Response 2:** We would like to thank you kindly. After careful review, we made the necessary revisions and corrections.

3. We suggest you thoroughly copyedit your manuscript for language usage, spelling, and grammar. If you do not know anyone who can help you do this, you may wish to consider employing a professional scientific editing service.

**Response 3:** We are grateful to thank you for your kindness and response. We have tried to correct your the languge thoroughly.

3. Your ethics statement should only appear in the Methods section of your manuscript. If your ethics statement is written in any section besides the Methods, please delete it from any other section.

**Response 2:** We have prepared it.

**Point-by-point response of Authors for editors' and reviewers' comment**
**Editor's Comments to the Authors**

Additional Editor Comments:

The authors are requested to address the reviewers' comments carefully and in detail

**Response:** We are grateful to thank you for your kindness and response. We tried to correct it thoroughly.

Dear Editors: We would like to notify you that, for more appropriate understanding of the paper we have revised the title to **“Geospatial Patterns, and Individual and Community Levels Factors of Cesarean Section Deliveries in Ethiopia: A Spatial and Multilevel Analysis** ”

**Response:** We are grateful to thank you for your kindness and response. We have tried to correct your concern and suggestion thoroughly.

**Reviewer Comment to the Authors**

**Abstract section**

**Comment 1** Abstract result section: this is presented differently, better to use commen words.

**Response 1:** We sincerely appreciate your thoughtful feedback. After careful review, we made the necessary revisions and corrections.

**Comment 2** Abstract conclusion section**:** The previous result no include the prevalence over time? So increase aspect is not clear please review.

**Response 2:** We extend our sincere gratitude for your insightful comment. We found it particularly valuable during the writing process of our work. We would like to sorry for making confuse in understanding. After careful review, we made the necessary revisions and corrections.

**Comment 3:** Abstract conclusion section**:** This sentence is not complete

**Response 3:** We sincerely appreciate your thoughtful comment. In response to your concern, since we found it particularly valuable during the writing process of our work and we would like to sorry for making confuse in understanding. After careful review, we made the necessary revisions and corrections.

**Introduction section**

**Comment 4.** Lesthan 5% people . It is not clear (people or pregnant women)

**Response 4:** We would like to thank you kindly for your comment. We had revised and corrected it thoroughly. Our interest is to say, according to the WHO statement, CS prevalence less than 5% among reproductive age mothers in the country or specific community indicates unmet need, whereas a prevalence of CS greater than 15% among reproductive age mothers might put the mother and new birth at risk.

**Comment 5:** population studies it is not clear?

**Response 5:** We would like to thank you kindly. We found it particularly valuable during the writing process of our work and we would like to sorry for making confuse in understanding. After careful review, we made the necessary revisions and corrections.

**Comment 6:** what is negative if CS rate lower infant death? It is not clear

**Response 6:** We express our gratitude for your insightful comment. We found it particularly valuable scientific plausibility of the paper and we would like to sorry for making confuse in understanding. After careful review, we made the necessary revisions and corrections.

**Comment 7:** Which WHO survey?

**Response 7:** First and foremost, we express our sincere gratitude for your intriguing idea and thoughtful questions. We have gained valuable insights from it. After careful review, we made the necessary revisions and corrections.

**Comment 8:** line 70 Explaine more.

**Response 8:** We sincerely appreciate your thoughtful comment. After careful review, we made the necessary revisions and corrections.

**Comment 9: line 75** Start this in new paragraph given that is different topic.

**Response 9:** We express our sincere gratitude for your insightful comment. We have gained valuable insights from it. After careful review, we made the necessary revisions and corrections.

**Comment 10. Line 77** Does it mean the rate of CS decreasing?

**Response 10:** We express our sincere appreciation for your insightful comment. We would like to sorry for making confuse in understanding. The rate of CS delivery in Ethiopia is increasing from 0.7 to 1.9 in 2000 to 2016 EDHS.

**Commentm 11: line 80 write the numper in parenthesis after country name.**

**Response 11:** We extend our sincere appreciation for your thoughtful comment. In response to your query, we made the necessary revisions and corrections..

**Comment 12: introduce the mini EDHS data.**

**Response 12:** We express our sincere appreciation for your insightful comment. Following your feedback, we have made revisions and incorporated your comment in manuscript.

**Method section**

**Comment13:** you have to explaine it first MEDHS?

**Response 13:** First and foremost, we express our sincere gratitude for your intriguing idea and thoughtful questions. In response to your query, we have made revisions and incorporated your comment in manuscript.

**Comment 14:** please explain more your sampling and sample size determination.

**Response 14:** We would like to thank you kindly for your deep comment. In response to your query, we have made revisions and incorporated your comment in manuscript. Please look the sampling procedure depiceted in figure 1 in detail

**Comment 15. Line 120:** no need of parenthesis

**Response 15:** We sincerely appreciate your thoughtful comment. The issue you raised is indeed intriguing. we have made revisions and incorporated your comment in manuscript.

**Comment 16.** Line 61: In which table please spacify

**Response 16:** We sincerely appreciate your thoughtful comment. We provided in table 2 and 3 Please look at table a 2 and 3

**Comment 17:** Line 131 please explaine more types of spatial statistics

**Response 17:** We sincerely appreciate your thoughtful comment. We have carefully reviewed and made the necessary corrections.

**Comment 18.** Please write the equation in new line

**Response 18:** We sincerely appreciate your thoughtful comment. We have carefully reviewed and made the necessary corrections.

**Comment 19.** Standar error never more than ±2

**Response 19:** We sincerely appreciate your thoughtful comment. Multicolinarity in logistic analysis is not estimated using variance inflaction factor (VIF). In this we consider the standard error of the variable. If the standard is below -2 and above 2 it indicates multiconiarity but it is does not mean that standard error never less than -2 or greater 2.

**Result Section**

**Comment 20.** Please edit more and please specify the delivery year before the DHS survey.

**Response 20:** We sincerely appreciate your thoughtful comment. We have carefully reviewed and made the necessary corrections.

**Discusion Section**

**Comment 21.** What was the previous CS delivery in Ethiopia please write

**Response 21:** We would like to thank you kindly for your deep comment. We have carefully reviewed and made the necessary corrections.

**We feel all of you may understand our feelings and interests.**

**We are thankful for all!!**
