# Supplementary material for: Geospatial patterns, and individual and community levels factors of cesarean section deliveries in Ethiopia: A spatial and multilevel analysis
Source: PLoS One. 2024 Aug 27;19(8):e0306052. doi: 10.1371/journal.pone.0306052 (PMC11349178; doi:10.1371/journal.pone.0306052)
Supplement: Attachment — Submitted filename: Author response.docx [file pone.0306052.s004.docx]

**Authors’ Responses to Editor and Reviewers Comments**

“**Geospatial Patterns, and Individual and Community Levels Factors of Cesarean Section Deliveries in Ethiopia: A Spatial and Multilevel Analysis”**

Dear Plos One Editors and Reviewers;

We are thankful for your constructive comments. We have looked at the comments and have revised our paper accordingly. We hope our paper improved as a result of incorporating the reviewer’s and academic editor’s comments and suggestions. Here are the authors’ responses to the comments.

Please find for your kind consideration the following:

1. A revised manuscript without track changes.
2. A revised paper with tracked changes
3. A rebuttal letter that responds to each point raised by the academic editor and reviewer.

The point-by-point responses of authors are written by hoping these changes would meet with your favorable consideration, we are happy to hear if there are more comments and suggestions. Please do not hesitate to let us know if you have any questions.

Yours Sincerely

Mr. Abel Endawkie Correspondence Author

Department of Epidemiology and Biostatistics School of Public Health College of Medicine and Health Science Wollo University Dessie Ethiopia

Tel. 251935459310	Email address abelendawkie@gmail.com

We have tried our best to improve it accordingly:

**Point-by-point response of Authors for editor's and reviewer's comment**
**Editors**

Thank you for submitting your manuscript to PLOS ONE. After careful consideration, we feel that it has merit but does not fully meet PLOS ONE’s publication criteria as it currently stands. Therefore, we invite you to submit a revised version of the manuscript that addresses the points raised during the review process (some comments you have already addressed).

**Editor comment:** Please check in particular for a comment about how CS has increased way beyond the WHO estimates, in particular in urban areas and in some parts of the world where there is more access to Operating Theaters and Obstetricians who can perform the operation. It is known that both the medical practitioners as well as the clients themselves decide to perform it for financial advantage, convenience, etc., and other factors that are not medically justified. Please review the literature and include this aspect. Please also see a few minor comments in the file attached.

**Response:** We are grateful to thank you for your kindness, response, insight, and suggestion. Let’s delve into why CS has increased way beyond the WHO estimates, in particular in urban areas and in some parts of the world where there is more access to Operating Theaters and Obstetricians who can operate. The cesarean sections (CS) have increased significantly beyond the WHO estimates, especially in urban areas and regions with greater access to Operating Theaters and Obstetricians capable of performing the procedure could be attributed to various factors, including financial incentives for medical practitioners and convenience for both healthcare providers and clients. Sometimes may be due to non-medical justifications such as financial gain, convenience, and other factors that do not align with medical necessity. Dear editor we incorporate this literature in our manuscript and specialy we cite in the discussion sec tion of our finding. Thank you a lot.

Reference:

1. Boerma, T., Ronsmans, C., Melesse, D. Y., Barros, A. J., Barros, F. C., Juan, L., ... & Temmerman, M. (2018). Global epidemiology of use of and disparities in caesarean sections. The Lancet, 392(10155), 1341-1348.

2. Betrán, A. P., Ye, J., Moller, A. B., Zhang, J., Gülmezoglu, A. M., & Torloni, M. R. (2016). The increasing trend in caesarean section rates: global, regional and national estimates: 1990-2014. PloS One, 11(2), e0148343.

3. Vogel, J. P., Betrán, A. P., Vindevoghel, N., Souza, J. P., Torloni, M. R., Zhang, J., ... & Temmerman, M. (2015). Use of the Robson classification to assess caesarean section trends in 21 countries: a secondary analysis of two WHO multicountry surveys. The Lancet Global Health, 3(5), e260-e270.

4. Molina, G., Weiser, T. G., Lipsitz, S. R., Esquivel, M. M., Uribe-Leitz, T., Azad, T., ... & Gawande, A. A. (2015). Relationship between cesarean delivery rate and maternal and neonatal mortality. JAMA, 314(21), 2263-2270.

**Reviewers comment**

**Reviewer #1 comment**

**General comment**: Overall I appreciate your work that you try to assess the geospatial, individual and community factors that affect CS deliveries in Ethiopia using the recent (2019) mini EDHS. It is very interested. But I have some comments and suggestions below.

**Response:** We are grateful to thank you for your kindness and response. We have tried to correct your insightful comment thoroughly.

**Comment** 1: In line 13, there is numeric number 4, what does that mean?? if it is an editing problem remove it

**Response 1:** We are grateful to thank you for your kindness and response. This is improper placement to show author affiliation order. After careful review, we made the necessary revisions and corrections.

**comment** 2: In your abstract of background section(from line 22-25) , the paragraph is not well written, as the abstract is the blue print of the whole document, it should describe document shortly and contain the introduction, the statement of problem specially the magnitude and severity of problem and significance of the study clearly in one paragraph. please rewrite it again

**Response 2:** We extend our sincere gratitude for your insightful comment. After careful review, we made the necessary revisions and corrections. We rewrite the as” Cesarean Section (CS) is the most popular surgery worldwide in obstetric care to save a mother's or the fetus's life. The prevalence of CS delivery in Ethiopia was 0.7% and 1.9% in 2000 and 2016 respectively and its spatial distribution and variation in Ethiopia are limited. This study provides evidence for healthcare providers and pregnant women on the national CS geospatial distribution and variation to promote evidence-based decision-making and improve maternal and neonatal outcomes**.** Therefore, this study aimed to determine geospatial patterns and individual and community-level factors of CS deliveries in Ethiopia”.

**Comment** 3: In abstract of methods section from line 28-29, which model did you use to identify the hotspot and cold spot areas and explore the spatial distribution of CS in Ethiopia? And which types of multilevel analysis model did you use to identify factors affecting CS in Ethiopia?

**Response 3:** We extend our sincere gratitude for your insightful comment. We used the spatial hotspot analysis using Getis-Ord Gi* hot spot analysis of ArcGIS version 10.7.1 to examine where spatial clusters of CS delivery occur on the landscape by identifying areas with statistically significant clustering of relatively high values (hot spots) or relatively low values (cold spots).

**Comment** 4: Line 31, you try to figure the prevalence of CS in Ethiopia, but in your title I didn’t see the prevalence of CS in Ethiopia?? Why?

**Response 4:** We extend our sincere gratitude for your insightful comment. We understand and value your comment. To make short our title and the usual work, we believe the spatial pattern indicates the descriptive spatial distribution which introduces the prevalence of CS and spatial variation of CS delivery in Ethiopia. We appreciate your concern and suggestion.

**Comment** 5: Line 53, not start with abbreviation after(.) if you want to use it write it as full phrases

**Response 5:** We extend our sincere gratitude for your insightful comment. After careful review, we made the necessary revisions and corrections.

**Comment** 6: Line 53-55 you sated that CS is done for maternal and fetal reasons and it is more frequent than before and the trend is expected to continue in the next ten years, WHY??

**Response 6:** We extend our sincere gratitude for your insightful comment. Even though there is still a significant lack of safe CS in resource-constrained nations, the possible reason is particularly in urban areas and in some parts of the world, cesarean sections (CS) will be increased significantly beyond the WHO estimates, especially in urban areas and regions with greater access to Operating Theaters and Obstetricians capable of performing the procedure(1). The rise in CS rates will be attributed to various factors, including financial incentives for medical practitioners and convenience for both healthcare providers and clients. In some parts of the world, CS rates have surged due to non-medical justifications such as financial gain, convenience, and other factors that do not align with medical necessity.

Beyond this there number of reasons listed in the literature.

1. Increasing maternal age: As more women are delaying childbirth to later in life, there is a higher likelihood of complications during pregnancy and delivery, leading to a higher rate of cesarean sections for maternal and fetal reasons.
2. Rising rates of obesity: The global increase in obesity rates has been associated with a higher risk of pregnancy complications, such as gestational diabetes and hypertension, which may necessitate the use of cesarean sections for the health and safety of both the mother and the baby.
3. Medical advancements: Advances in medical technology and techniques have made cesarean sections safer and more accessible, leading to an increase in the frequency of the procedure for both maternal and fetal indications.
4. Changing attitudes towards childbirth: There is a shift in societal norms and preferences towards elective cesarean sections for reasons such as convenience, fear of labor pain, or perceived control over the birthing process, contributing to the overall increase in cesarean section rates
5. Legal considerations: Concerns over potential litigation in cases of adverse outcomes during vaginal delivery may lead healthcare providers to opt for cesarean sections more frequently, particularly when there are perceived risks to the health of the mother or the baby.

**Comment** 7: Line 55, reference?

**Response 7:** We extend our sincere gratitude for your insightful comment. After careful review, we made the necessary revisions and corrections.

**Comment** 8: There is the redundancy e.g from line 62-65 there is the prevalence is used three times in one paragraph , please paraphrase it

**Response 8:** We extend our sincere gratitude for your insightful comment. After careful review, we made the necessary revisions and corrections.

**Comment** 9: Line 68, where this population based study was conducted??

**Response 9:** We extend our sincere gratitude for your insightful comment. Dear reviewer stating the specific area is more informative for the reader. However, the reason why we were not writing was due to we believed there was a reference. Since we believe it is a valuable and reasonable suggestion we made the necessary revisions and corrections. A worldwide population‐based ecological study………

**Comment** 10: Line 71, if you use the abbreviation CS omit the word section next to it

**Response 10:** We extend our sincere gratitude for your insightful comment. After careful review, we made the necessary revisions and corrections.

**Comment** 11. Line 74 either maternal health or health of mother is enough, so remove one

**Response 11**: We extend our sincere gratitude for your insightful comment. After careful review, we made the necessary revisions and corrections.

**Comment** 12. Once you abbreviate using the full phrase, abbreviating it using same fashion is not necessary, means, in line 66 you abbreviate the EDHS with its full phrase and repeate it in line 85-86.

**Response 12**: We extend our sincere gratitude for your insightful comment. After careful review, we made the necessary revisions and corrections

**Comment** 13: Generally, your introduction part is not consistent or well written, meaning in some paragraphs you talk about the necessity of CS and its importance, in other paragraphs you talk about its complication. This two ideas will contradicted.

**Response 13:** We extend our sincere gratitude for your insightful comment. After careful review, we made the necessary revisions and corrections. Generally, CS is important to save life of the infant and mother. However, if it is surged due to non-medical justifications such as financial gain, convenience, and other factors that do not align with medical necessity will end up with maternal health problems. Therefore this study emphasizes the need for prodding evidence for healthcare providers and pregnant women on the national CS geospatial patterns, and individual and community level factors to promote evidence-based decision-making and improve maternal and neonatal outcomes. We would like to provide evidence for the police maker.

The higher CS delivery beyond the WHO estimates, especially in urban areas and regions with greater access to Operating Theaters and Obstetricians capable of performing the procedure could be attributed to various factors, including financial incentives for medical practitioners and convenience for both healthcare providers and clients and surged due to non-medical justifications such as financial gain, convenience, and other factors that do not align with medical necessity [4, 38]. However, lower CS delivery” below the WHO estimates”, especially in rural and remote areas may be due to inadequate and/or sparse availability of emergency obstetrics services, inadequate equipment and medication in obstetric emergency centers, a shortage of skilled birth attendants and services, long travel distances, and unsuitable terrain that could provide a substantial geographic barrier to emergency obstetric treatment [4, 39, 40]”. Therefore, efforts should be geared towards strategies and interventions to ensure fair access to CS delivery in line with WHO recommendations, especially in the regions where the CS delivery rate is below WHO estimates in Tigray, Amhara, Afar, and Benishangul Gumze regions

**Comment** 14. In your result section use table format

**Response 14:** We extend our sincere gratitude for your insightful comment. After careful review, we made the necessary revisions and corrections.

**Reviewer #2 comment:**

Dear Author, I commend you for your work, which is substantial. However, the manuscript is not well organized, and it looks as if the version submitted is a draft:

**Response:** We extend our sincere gratitude for your insightful comment. After careful review, we made the necessary revisions and corrections.

**Comment** 1. There are unnecessary line numbering that need to be removed across the manuscript.

**Response 1:** We extend our sincere gratitude for your insightful comment. After careful review, we made the necessary revisions and corrections.

**Comment** 2. The headlines and titles are not numbered in a logical sequence.

**Response 2:** We extend our sincere gratitude for your insightful comment. After careful review, we made the necessary revisions and corrections.

**Comment** 3. There are crossed sentences (in red) from previous reviewers with corrections not applied(I have previously reminded you to look into the literature because in urban areas, with more educated mothers, they often request to be delivered by CS, plus there is an issue of more non-medically indicated CS by doctors in areas of higher socioeconomic conditions)

**Response 3:** We extend our sincere gratitude for your insightful comment. After careful review, we made the necessary revisions and corrections. I cited the literature indicating your query. CS delivery also may be associated especially in urban areas and regions with greater access to Operating Theaters and Obstetricians capable of performing the procedure, CS rates can be attributed to various factors, including financial incentives for medical practitioners and convenience for both healthcare providers and clients and non-medical justifications such as financial gain, convenience, and other factors that do not align with medical necessity [46, 47].

**We appreciate your valuable time and effort in reviewing our work!!**
